# Whole-Genome Sequencing of *Acer catalpifolium* Reveals Evolutionary History of Endangered Species

**DOI:** 10.1093/gbe/evab271

**Published:** 2021-12-08

**Authors:** Tao Yu, Yiheng Hu, Yuyang Zhang, Ran Zhao, Xueqing Yan, Buddhi Dayananda, Jinpeng Wang, Yuannian Jiao, Junqing Li, Xin Yi

**Affiliations:** 1 Beijing Key Laboratory for Forest Resources and Ecosystem Processes, Beijing Forestry University, China; 2 State Key Laboratory of Systematic and Evolutionary Botany, Institute of Botany, The Chinese Academy of Sciences, Beijing, China; 3 University of Chinese Academy of Sciences, Beijing, China; 4 The National-Local Joint Engineering Laboratory of High Efficiency and Superior-Quality Cultivation and Fruit Deep Processing Technology on Characteristic Fruit Trees, College of Plant Science, Tarim University, Alear, China; 5 School of Agriculture and Food Sciences, The University of Queensland, Brisbane, Queensland, Australia

**Keywords:** *Acer catalpifolium*, small population size, positive selection, gene family evolution, comparative genomics

## Abstract

*Acer catalpifolium* is an endangered species restricted to remote localities of West China. Understanding the genomic content and evolution of *A. catalpifolium* is essential to conservation efforts of this rare and ecologically valuable plant. Here, we report a high-quality genome of *A. catalpifolium* consisting of ∼654 Mbp and ∼35,132 protein-coding genes. We detected 969 positively selected genes in two *Acer* genomes compared with four other eudicots, 65 of which were transcription factors. We hypothesize that these positively selected mutations in transcription factors might affect their function and thus contribute to *A. catalpifolium’*s decline-type population. We also identified 179 significantly expanded gene families compared with 12 other eudicots, some of which are involved in stress responses, such as the FRS–FRF family. We inferred that *A. catalpifolium* has experienced gene family expansions to cope with environmental stress in its evolutionary history. Finally, 109 candidate genes encoding key enzymes in the lignin biosynthesis pathway were identified in *A. catalpifoliu*m; of particular note were the large range and high copy number of cinnamyl alcohol dehydrogenase genes. The chromosome-level genome of *A. catalpifolium* presented here may serve as a fundamental genomic resource for better understanding endangered *Acer* species, informing future conservation efforts.

Significance

The population of the endangered species *Acer catalpifolium* is extremely small, and conservation efforts have been limited to traditional methods because genomic information has been unavailable. In this study, we sequenced and assembled a high-quality genome of *A. catalpifolium*, detected selection pressure signals in the domains of some important transcription factors that may contribute to functional deficiency in *A. catalpifolium*, and found a far-red light-responsive gene family that has been significantly expanded. With genome data about *A. catalpifolium* now available, we can combine omics analyses with transgenic/CRISPR approaches in the future to save this plant from extinction and recover the population, which is important for the overarching goal of biodiversity conservation.

## Introduction

The genus *Acer* (maple) belongs to the family *Sapindaceae* and consists of nearly 200 species (Piao et al. 2020). Maples form some of the largest broad-leaved deciduous tree population in eastern Asia, North America, and Europe (Wolfe and Tanai 1987; van Gelderen et al. 1994). Maple trees are key resources for commercial products; their bark is used for sugar and dye, ashes for soap, and trunks for pulpwood and lumber (Gelderen and Gelderen 1999). Species in the *Acer* genus have a diverse range of leaf morphologies, as well as complex reproductive systems with unisexual, polygamodioecious, or androdioecious flowers (Shang et al. 2012; Rosado et al. 2018). Thus, these species are ideal specimens for the study of variation in leaf morphology and plant sexual diversity. *Populus trichocarpa* (black cottonwood), a well-known deciduous broadleaf tree, is usually used to make crates, pallets, and paper; comparatively, some maples (like bigleaf maple and silver maple) have different mechanical properties, including larger values for modulus of elasticity and modulus of rupture, which means that they perform better than *P. trichocarpa* in terms of both wood strength and stiffness (Ross 2010). According to the U.S. Department of Agriculture, a large proportion of maple lumber is further manufactured into a variety of products, including flooring and furniture (Betts 1959), making *Acer* an ideal genus to study properties of wood.

**Fig. 1.**
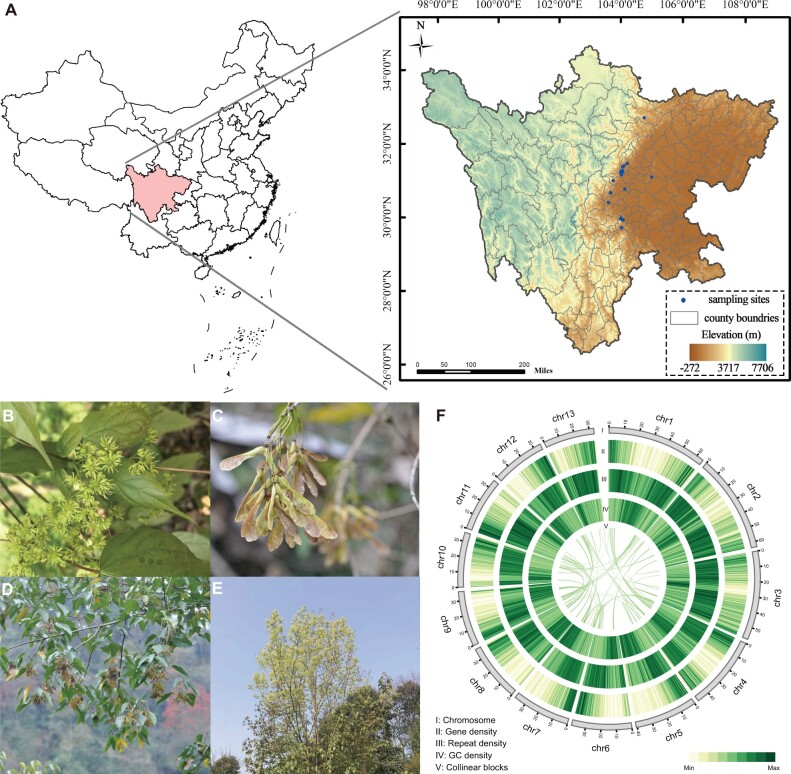
Geographical distribution, morphology, and characteristics of the assembled *A. catalpifolium* genome. (*A*) Geographic distribution of *A. catalpifolium* in Sichuan province. Blue points indicate regions of *A. catalpifolium* populations. Pictures of four different tissues of *A. catalpifolium*: flowers (*B*), samara (*C*), branches with samara (*D*), and flowering tree (*E*). (*F*) The genomic features of *A. catalpifolium*. Tracks I through V represent the name and size of the 13 chromosomes, gene density, repeat density, GC content density, and collinear blocks, respectively, which were calculated in 500-kb windows.

Maple trees mainly grow in temperate and subtropical areas of the Northern Hemisphere, and there are some species that are native to Asia. For example, in China, some *Acer* species are widely distributed (*A. amplum*, *A. longipes*, *A. mono*, and *A. truncatum*), whereas others are endangered (*A. catalpifolium*, *A. miaotaiense*, and *A. yangjuechi*) (Bi et al. 2016). *Acercatalpifolium* is a deciduous tree that is under threat of extinction (Yu et al. 2020) and is narrowly distributed in the rainy zone of western China (28°30′–33°N, 102°30′–104°E). *Acercatalpifolium* has a straight tree trunk and umbrella-shaped crown and usually grows up to 25 m tall (fig. 1*A*). It has papery leaves with ovate or ovate-oblong leaf blades, yellow-green flowers, yellowish, and glabrous samaras with spreading wings (fig. 1*B*–*E*) (Xu et al. 2008). Because of *A. catalpifolium’*s beautiful appearance, local people in the western Sichuan province sometimes plant them along roadsides (FOC, http://www.iplant.cn/, last accessed December 13, 2021).

The population size of *A. catalpifolium* has decreased steadily. In 2009, it was listed in the Wild Plants with Extremely Small Populations (WPESP) Rescue and Protection Plan (The State Forestry Administration of The People's Republic of China 2009). Scientists have carried out many studies on *A. catalpifolium* to understand this population decline. The possible negative effects of decreased seed germination, shade from neighboring dominant species, and severe human disturbance on *A. catalpifolium* growth and biomass accumulation have been researched (Zhang et al. 2019; Song and Liu 2020). Moreover, population structure analysis shows that *A. catalpifolium* has a tendency to convert from a stable population to a decline-type population (Zhang et al. 2018; Xu and Liu 2019). Therefore, it is imperative to understand why the population of *A. catalpifolium* continues to decrease and how we might be able to intervene, interrupt, or even reverse this population decline. Traditional studies on plant phenotypes and the surrounding environment have begun to tackle these questions, and to continue this research it is essential to assemble a high-quality genome. This will allow us to investigate the possible genetic causes of population decline in *A. catalpifolium* and inform future research to recover the population.

Here we report a high-quality, chromosome-level genome assembly of *A. catalpifolium* using long- and short-read sequencing methods. The genome data allowed us to understand the molecular evolutionary history of *A. catalpifolium*, especially genome structure variation and gene family evolution. Comparisons between the genome of *A. catalpifolium* and the previously published *A. yangbiense* genome (Yang et al. 2019) provided us an opportunity to directly identify genomic differences between the two endangered species and hypothesize about the impact of these differences on population size. Some positively selected genes in *A. catalpifolium* contained mutations in functional domains, which may cause gene functional deficiency. Because a high-quality genome of *A. catalpifolium* is now available, additional omics analyses can be performed to help improve the survival rate of the tree and save this endangered plant from extinction, which is important for the conservation of biodiversity on Earth.

## Results

### Genome Assembly and Annotation of *A. catalpifolium*

By integrating PacBio Sequel long reads (∼34.39 Gb), Illumina short reads (∼34.40 Gb), and Hi-C paired-end reads (∼45.55 Gb), we assembled a chromosome-level reference genome of *A. catalpifolium* (supplementary tables S1 and S2, Supplementary Material online). The genome size of *A. catalpifolium* was estimated to be about 650.47 Mb based on 19-mer frequency distribution (supplementary fig. S1, Supplementary Material online). PacBio long reads were assembled de novo, and assembled contigs were polished with Illumina short reads (supplementary fig. S2). The resulting genome assembly comprised 3,112 contigs with a contig N50 value of ∼0.66 Mb. The sequences were further scaffolded with Hi-C reads. Ultimately, a genome assembly of 654.51 Mb in length was produced, consisting of 1,978 scaffolds (with a scaffold N50 of 37.58 Mb) (table 1), 86% of which were anchored into 13 pseudo-chromosomes. Benchmarking Universal Single-Copy Orthologs (BUSCO) assessment estimated that the final assembled genome was ∼93.3% complete (supplementary table S3, Supplementary Material online).

**Table 1:** Statistical Results of *A. catalpifolium* Genome Assembly

	Contigs	Scaffolds
Total number of sequences	3,112	1,978
Assembly size (bp)	654,441,387	654,514,841
N50-length (bp)	657,354	37,579,358
N90-length (bp)	107,679	164,478
Maximal length (bp)	6,300,028	64,842,224
GC content (%)	35.25	35.25
Total gap length (bp)	–	73,454

In total, we annotated 35,132 protein-coding genes using a combination of ab initio, homology-based, and transcriptome-based gene prediction (supplementary table S4, Supplementary Material online). Of the predicted protein-coding genes, 93.04% were functionally annotated using at least one of the following databases: TrEMBL (Apweiler et al. 2004), SwissProt (Apweiler et al. 2004), Pfam (Mistry et al. 2021), NR/NT (Pruitt et al. 2007), KOG/KEGG (Kanehisa et al. 2016), and GO (Ashburner et al. 2000) (supplementary table S5, Supplementary Material online). Furthermore, 59% of the *A. catalpifolium* genome consists of repetitive elements, which is a slightly lower percentage than that of *A. yangbiense* (68%) (fig. 1*F*;supplementary table S6, Supplementary Material online). The majority of these repeats were long-terminal repeat (LTR) retrotransposons (39%) and Penelope-like elements (10.49%). Of the identified LTRs, 62.7% belonged to *Copia*, whereas 36.4% belonged to *Gypsy* (supplementary table S6, Supplementary Material online).

### Comparative Genomics Between *A. catalpifolium* and *A. yangbiense*

**Fig. 2.**
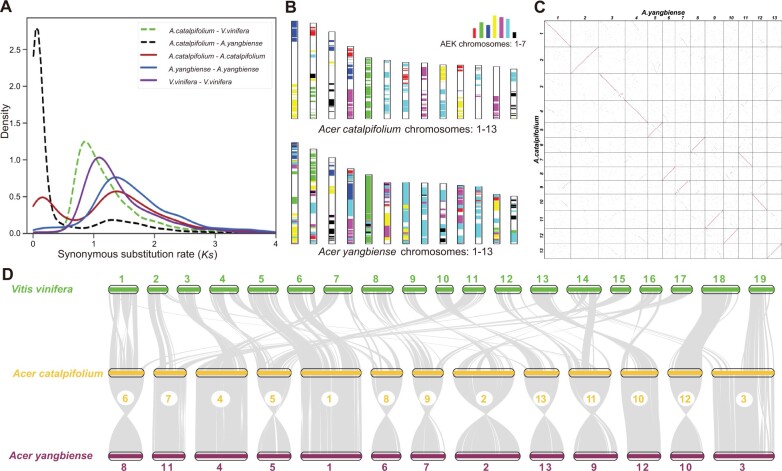
Collinear survey to understand the evolutionary history of *A. catalpifolium*. (*A*) *Ks* distributions for paralogs found in collinear regions of *A. catalpifolium*, *A. yangbiense*, and *V. vinifera*, and for orthologs between *A. catalpifolium* and the other two plants. (*B*) Comparison with AEK chromosomes revealed syntenic blocks in *A. catalpifolium* and *A. yangbiense*. (*C*) A dot plot of pairwise orthologs showed a 1:1 chromosomal relationship between *A. catalpifolium* and *A. yangbiense*. (*D*) Genome-wide macrosynteny among *A. catalpifolium*, *A. yangbiense*, and *V. vinifera*. Gray lines represent major syntenic blocks.

To understand the evolutionary history of *Acer*, we performed inter- and intragenomic comparisons among *A. catalpifolium*, *A. yangbiense*, and *Vitis vinifera*. The synonymous substitutions per synonymous site (*Ks*) distributions of the orthologs and paralogs were investigated. With intragenomic comparisons, we detected 3,311, 2,445, and 2,288 syntenic paralogs in *A. catalpifolium*, *A. yangbiense*, and *V. vinifera*, respectively. The *Ks* distribution plot showed significant peaks at approximately 1.4 for paralogous gene pairs in both *A. catalpifolium* and *A. yangbiense*, and at 1.1 for *V. vinifera* paralogs. The peak for *Ks* distribution of *A. catalpifolium* and *V. vinifera* orthologs was at approximately 0.9, and the peak for *A. catalpifolium* and *A. yangbiense* orthologs was at approximately 0.05 (fig. 2*A*). The *Ks* analyses showed that the most recent whole-genome duplication (WGD) event in *A. catalpifolium*, as well as in *A. yangbiense*, was the well-studied gamma event shared by core eudicots (Jaillon et al. 2007), providing no evidence for further lineage-specific WGD in *Acer*. To further investigate this, we also performed intergenomic syntenic comparisons using MCScanX with default parameters (Wang et al. 2012). We found a 1:1 syntenic depth ratio between *V. vinifera* to *A. catalpifolium* and *A. yangbiense*, which supported the findings from the *Ks* analyses; these results indicated that there was not a lineage-specific WGD after the divergence of *A. catalpifolium*, *A. yangbiense*, and *V. vinifera* (fig. 2*C* and *D*). Therefore, the genome of *A. catalpifolium* that lacks further WGD events since the origin of core eudicots may be representative of the ancestral genome of eudicots, facilitating investigation of their subsequent genome evolution.

Furthermore, *A. catalpifolium* and *A. yangbiense* genomes were compared with the previously reconstructed ancestral eudicot karyotype (AEK) genome (Murat et al. 2017). In *A. catalpifolium*, 5,153 genes had a syntenic relationship with AEK, whereas in *A. yangbiense*, 6,869 syntenic genes were identified. Although there were fewer *A. catalpifolium* genes in syntenic regions compared with *A. yangbiense*, a clearer 3:1 syntenic depth ratio was inferred in *A. catalpifolium*-AEK compared with *A. yangbiense*-AEK (fig. 2*B*). Then, 20,505 orthologous gene pairs were plotted, indicating a high similarity of genome structures between *A. yangbiense* and *A. catalpifolium*. Chromosome-level macrosynteny further suggests a high level of conserved genome structure between the two species and few small-scale genomic rearrangements (fig. 2*C* and *D*). This is likely due to the relatively short time of divergence between the two *Acer* species, discussed below. We also identified 3,299 and 3,653 tandem duplications in *A. catalpifolium* and *A. yangbiense*, respectively. When compared with *V. vinifera*, *A. catalpifolium* shared 25,855 syntenic gene pairs, and *A. yangbiense* shared 23,242 syntenic gene pairs. After filtering, 9,631, 9,200, and 15,529 best “one-to-one” syntenic matchings were obtained in *V. vinifera*–*A. catalpifolium*, *V. vinifera*–*A. yangbiense*, and *A. catalpifolium*–*A. yangbiense*, respectively. We integrated “one-to-one” reciprocal best BLASTP hits between *A. catalpifolium* and *A. yangbiense* with the previous results, and found that 395 genes were lost specifically in *A. catalpifolium*, whereas 848 genes were lost specifically in *A. yangbiense*. The majority of the genes that were well conserved amongst the three species provide an estimation of the ancestral eudicot gene content. The different numbers of lost genes between the two *Acer* species indicated different histories of genomic sequence evolution.

### Gene Family Evolution and Gene Expansion

To better understand the gene content and gene family evolution in *A. catalpifolium*, we used OrthoMCL v14.137 with default parameters (Li et al. 2003) to reconstruct the gene families of 13 angiosperms, including nine eudicots (*A. catalpifolium*, *A. yangbiense*, *Arabidopsis thaliana*, *Aquilegia coerulea*, *Citrus sinensis*, *Dimocarpus longan*, *P. trichocarpa*, *Solanum lycopersicum*, *V. vinifera*), three monocots (*Ananas comosus*, *Musa acuminata*, *Oryza sativa*), and an outgroup of one basal angiosperm, *Amborella trichopoda*. A total of 29,299 orthologous groups (orthogroups) were identified, 666 of which were specific to *A. catalpifolium* and 377 of which were specific to *A. yangbiense*. In addition, we found 781 gene families specific to *Acer* and 158 specific to Sapindales.

**Fig. 3.**
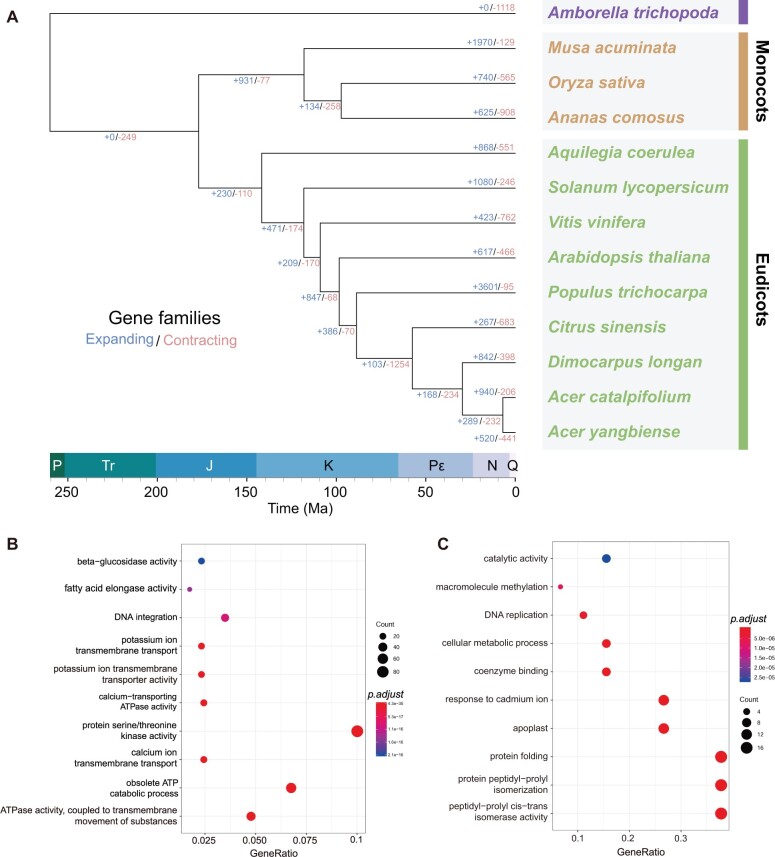
Phylogenetic and GO analysis of *A. catalpifolium* genome. (*A*) A phylogenetic species tree was constructed with 873 single-copy orthologs from 13 plant species. Gene family expansion and contraction are displayed as numbers at each node of the tree, with blue representing expansion and red representing contraction. The time scale at the bottom of the tree shows the timing of divergences in the plant lineages. P, Permian; Tr, Triassic; J, Jurassic; K, Cretaceous; Pε, Palaeogene; N, Neogene; Q, Quaternary. (*B*) Visualization of results from GO enrichment analysis of 179 significantly expanded gene families in *A. catalpifolium*. The top ten GO terms were selected for display after using Benjamini–Hochberg multiple test correction for *P* value adjustment (adjusted *P* value <0.05). (*C*) Visualization of the results from the GO enrichment analysis of 159 *Acer* genus-specific significantly expanded genes. The top ten GO terms were selected for display after using Benjamini–Hochberg multiple test correction for *P* value adjustment (adjusted *P* value <0.05).

To understand the patterns of gene family gains, losses, expansions, and contractions following the diversification of angiosperms, we first constructed a phylogenetic tree using 873 single-copy gene families from the 29,299 aforementioned orthogroups (fig. 3*A*). The single-copy genes were gathered to generate a supermatrix and put into RAxML (Stamatakis et al. 2005) for phylogeny analysis. Next, we estimated the divergence time using r8s (Sanderson 2003) with four fossil calibrations (see Materials and Methods for details). We found the divergence time between *A. catalpifolium* and *A. yangbiense* to be approximately 6.9 million years ago (Ma) and the time of divergence between the *Acer* genus and *D. longan* to be approximately 29.5 Ma (fig. 3*A*), which was consistent with previous research (Yang et al. 2019). We also extrapolated the divergence of the two *Acer* species to be approximately 5.2–5.9 Ma using two significant peaks of *Ks* distribution for *A. catalpifolium*–*A. yangbiense* and *V. vinifera*–*V. vinifera* (*Ks* peaks at ∼0.0497 and ∼1.092, respectively; see Materials and Methods for details), with the gamma event estimated to have occurred ∼115–130 Ma (Jiao et al. 2012; Wang et al. 2018).

Comparisons of the genomes of *A. catalpifolium* and 12 other species (*A. yangbiense*, *A. thaliana*, *A. coerulea*, *C. sinensis*, *D. longan*, *P. trichocarpa*, *S. lycopersicum*, *V. vinifera*, *A. comosus*, *M. acuminata*, *O. sativa*, and *A. trichopoda*) identified a total of 220 rapidly evolving orthogroups in *A. catalpifolium* (supplementary fig. S3, Supplementary Material online) using CAFÉ (Han et al. 2013). Of these 220 orthogroups, 179 were significantly expanded and 41 were significantly contracted (*P* value <0.01). Based on GO enrichment analysis using clusterProfiler (Yu et al. 2012), 2,156 *A. catalpifolium* genes in the 179 expanded orthogroups were highly enriched in “ATPase activity, coupled to transmembrane movement of substances” (*P* value = 4.34E−35) and “calcium ion transmembrane transport” (*P* value = 6.53E−23) (fig. 3*B*; supplementary figures S4, S5 and table S7, Supplementary Material online). It is important to note that ATPase and calcium ion transport are functionally related to phototropin-related light signal transduction pathways (Dodd et al. 2010; Inoue and Kinoshita 2017). Moreover, 159 *Acer*-specific *A. catalpifolium* genes in the significantly expanded orthogroups were highly enriched in “response to cadmium ion” (*P* value = 4.87E−11) and “response to stimulus” (*P* value = 4.08E−05) (fig. 3*C*;supplementary fig. S6 and table S8, Supplementary Material online). The specific gene family expansion might indicate certain environmental pressures during the evolutionary history of *A. catalpifolium*.

### Evidence of Positive Selection Across the *Acer* Genus at the Whole-Genome Level

**Fig. 4.**
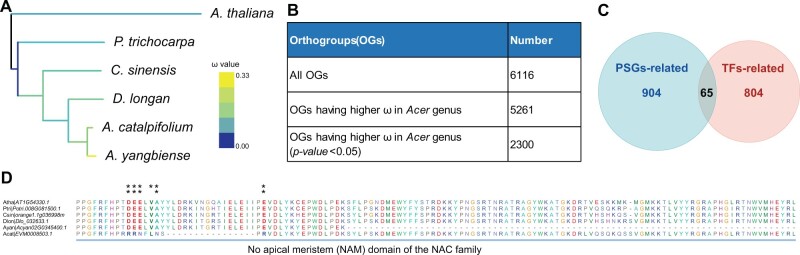
The *Ka*/*Ks* ratios were used to identify PSGs using single-copy orthogroups in six eudicots. (*A*) The *Ka*/*Ks* ratios (*ω* values) were estimated at each branch of the phylogenetic tree using six representative plants including two *Acer* species. The branches are colored according to the *ω* value in each branch. Blue represents the minimum value and yellow represents the maximum value. (*B*) A large proportion (5,261/6,116) of orthogroups had higher *Ka*/*Ks* ratios in the *Acer* genus than the remaining branches of the tree shown in figure 4*A*. All *Ka*/*Ks* ratios were calculated independently using 6,116 orthogroups. (*C*) A Venn diagram shows the overlap of PSGs-related orthogroups in the 6,116 orthogroups and TF-related orthogroups. The overlap number was obtained by comparing 969 orthogroups containing PSGs and 869 TF-related orthogroups. (*D*) Protein sequence alignments of the conserved domains in the PSGs of *A. catalpifolium* (*EVM0008503.1*) and orthologs in the five other species. Six genes belonged to one orthogroup annotated as the NAC gene family. One asterisk above the alignment means that the site was possibly under positive selection in *A. catalpifolium* (*P* value <0.05). Two asterisks above the alignment means that the site showed highly significant evidence of positive selection in *A. catalpifolium* (*P* value <0.01).

In domesticated species, it has been shown that decreased effective population size has led to an increase in the frequency of slightly deleterious variants near sites of positive selection (Liu et al. 2017). Given the declining trend of *A. catalpifolium’*s population size (Xu and Liu 2019), we investigated whether this association between positive selection and deleterious mutations exists in *A. catalpifolium*. The number of nonsynonymous substitutions per nonsynonymous site (*Ka*) was divided by the number of synonymous substitutions per synonymous site (*Ks*) to calculate *ω*. A super matrix was generated from single-copy orthogroups in the 13 selected species; the super matrix was then used to estimate *ω* using the “free-ratio” model of codeml in PAML with default parameters (Yang 2007). Our results clearly showed that the *ω* values of *Acer* species were generally larger than 0.23, whereas the *ω* values of other eudicot species were generally smaller than 0.20 (supplementary fig. S7, Supplementary Material online). In addition, we performed the same *ω* analysis with six additional eudicots (*A. thaliana*, *P. trichocarpa*, *C. sinensis*, *D. longan*, *A. catalpifolium*, and *A. yangbiense*) using *Arabidopsis* as an outgroup (Xu et al. 2017). The results revealed a similar pattern in which *Acer* species, in particular *A. yangbiense*, had larger *ω* values than other species (fig. 4*A*). Lastly, we estimated the *ω* values for each of the 6,116 modified single-copy orthogroups (see Materials and Methods for details) in the same six eudicots mentioned above using the “branch” model in PAML. Notably, 5,261 of the 6,116 orthogroups showed a larger *ω* ratio in the *Acer* lineages, 2,300 of which were statistically significant (*P* value < 0.05, χ^2^ test) (fig. 4*B*). Our results showed that *ω* is elevated in the *Acer* clade relative to the other examined species.

Both positive selection and relaxation of negative selection can lead to a higher *ω* ratio (Xu et al. 2017). To test if *A. catalpifolium* or *A. yangbiense* has experienced positive selection, the 6,116 aforementioned orthogroups in six eudicots were used to perform “branch-site” model analysis. In total, 969 orthogroups were identified that harbor codons with *ω *> 1 in the *Acer* clade (*P* value <0.05), 683 of which showed statistically significant evidence of positive selection (*P* value <0.01) (supplementary table S9, Supplementary Material online), 181 contained positively selected genes (PSGs) that were specifically identified in *A. catalpifolium* (supplementary table S10, Supplementary Material online), and 548 contained PSGs that were specifically identified in *A. yangbiense* (supplementary table S11, Supplementary Material online). Notably, there was only one gene, *EVM0028239.1*, that was lost specifically in *A. yangbiense* (supplementary fig. S8, Supplementary Material online) and was also positively selected in *A. catalpifolium* (supplementary fig. S9, Supplementary Material online). This gene has the predicted core ARD domain, but already shows evidence of mutation in the C-terminal of the domain, indicating that functional evolution of this gene might be ongoing in *A. catalpifolium*.

Based on TF annotation of *Arabidopsis* in PlantTFDB 5.0, 869 of the 29,299 aforementioned orthogroups were annotated as putative TFs. Among the 969 orthogroups having PSGs in *Acer*, we found that 65 were putative TFs (fig. 4*C*;supplementary table S12, Supplementary Material online). We investigated PSGs in those 65 putative TFs because they contained positively selected sites in the protein domain, representing different amino acids in *A. catalpifolium* or *A. yangbiense* compared with the other five species. The NAC (also named NAM, “No Apical Meristem”) family is one of the largest plant-specific TF families and is involved in embryonic, floral, and vegetative development, as well as defense and abiotic stress (Olsen et al. 2005). In the NAC gene family, the *Arabidopsis* gene *NAC DOMAIN CONTAINING PROTEIN 20* (*NAC020*, *AT1G54330.1*) has been proposed to be an early regulator of phloem sieve element (SE) differentiation (Kondo et al. 2016). The *NAC020* orthologous gene in *A. catalpifolium* (*EVM0008503.1*) was predicted to have six positively selected sites in the N-terminal of the NAM domain (fig. 4*D*). We compared the predicted 3D protein structure of these two genes using the trRosetta webserver (Du et al. 2021) and found a missing helix structure in *EVM0008503.1* (supplementary fig. S10, Supplementary Material online), which may cause protein function deficiency. For the MADS-box gene family, the *Arabidopsis* gene *AGAMOUS-LIKE 12* (*AGL12*, *AT1G71692.1*) plays an important role in root development and flowering transition (Tapia-Lopez et al. 2008). In the corresponding orthologs of *AGL12*, namely *Acyan10G0161700.1*, eight positively selected sites in the N-terminal of the K-box domain were identified. In addition, many conserved amino acids were absent in the K-box domain of *Acyan10G0161700.1* (supplementary fig. S11, Supplementary Material online), leading to a shorter helix structure in *A. yangbiense* (supplementary fig. S12, Supplementary Material online). Mutations found in the protein domain of some PSGs in both *Acer* species have influenced the 3D structure of the proteins, and may further affect their biological functions.

### Evolutionary History of FRS–FRF Gene Family

Considering that the natural habitat of *A. catalpifolium* has high canopy coverage and limited access to sunlight (Zhang et al. 2018), we specifically examined the gene families that function in shade avoidance syndromes. It has been reported that the FAR1-RELATED SEQUENCE (FRS) and FRS-RELATED FACTOR (FRF) gene families are involved in multiple shade-avoidance responses(Liu et al. 2019; Xie et al. 2020). In *Arabidopsis*, FRS–FRF genes are reported to be regulated by far-red light (Lin and Wang 2004; Lin et al. 2007). We identified FRS–FRF family members in *A. catalpifolium* and *A. yangbiense* based on sequence similarity and protein domain conservation using the 18 FRS–FRF genes in *Arabidopsis* as references (Ma and Li 2018). In total, we identified 93 and 68 members of the FRS–FRF gene family in the *A. catalpifolium* and *A. yangbiense* genomes, respectively.

**Fig. 5.**
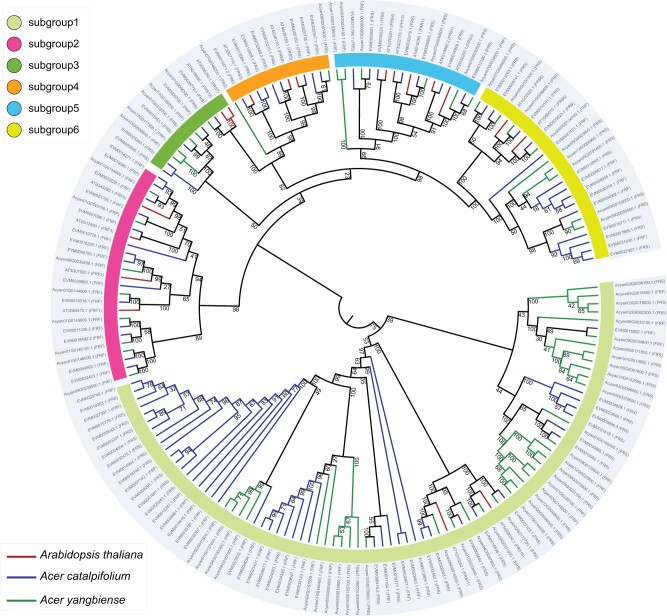
Phylogenetic tree of the 179 FRS–FRF family genes in *Arabidopsis*, *A. catalpifolium*, and *A. yangbiense*. The phylogenetic tree was constructed using predicted FRS–FRF genes in *A. catalpifolium* and *A. yangbiense*, as well as confirmed FRS–FRF genes in *Arabidopsis*, using the GTRGAMMA model of RAxML (Stamatakis et al. 2005). The six subgroups are indicated by six different colors, consistent with previous subdivisions of the FRS–FRF gene family in *Arabidopsis* (Ma and Li 2018).

Amino acid sequences of the FRS–FRF family members in *Arabidopsis*, *A. catalpifolium*, and *A. yangbiense* (supplementary fig. S13, Supplementary Material online) were aligned and used to construct a phylogenetic tree. According to the phylogenetic organization of the 18 FRS–FRF *Arabidopsis* genes (Ma and Li 2018), we divided the FRS–FRF genes in this study into six subgroups (fig. 5). Compared with *A. yangbiense* and *Arabidopsis*, the FRS–FRF gene family was expanded in *A. catalpifolium*, particularly in subgroup 4 (fig. 5). We further investigated the types of gene duplication evident in the FRS–FRF family. Duplication through repetitive sequence was the main mechanism in both *Acer* trees, followed by tandem duplication (supplementary fig. S14, Supplementary Material online). This expansion of the FRS–FRF family might facilitate gene expression divergence or gene function diversification, which have contributed to environmental adaptation in the evolutionary history of *A. catalpifolium*. Further functional validation experiments are needed to investigate this.

### Lignin Biosynthesis

**Fig. 6.**
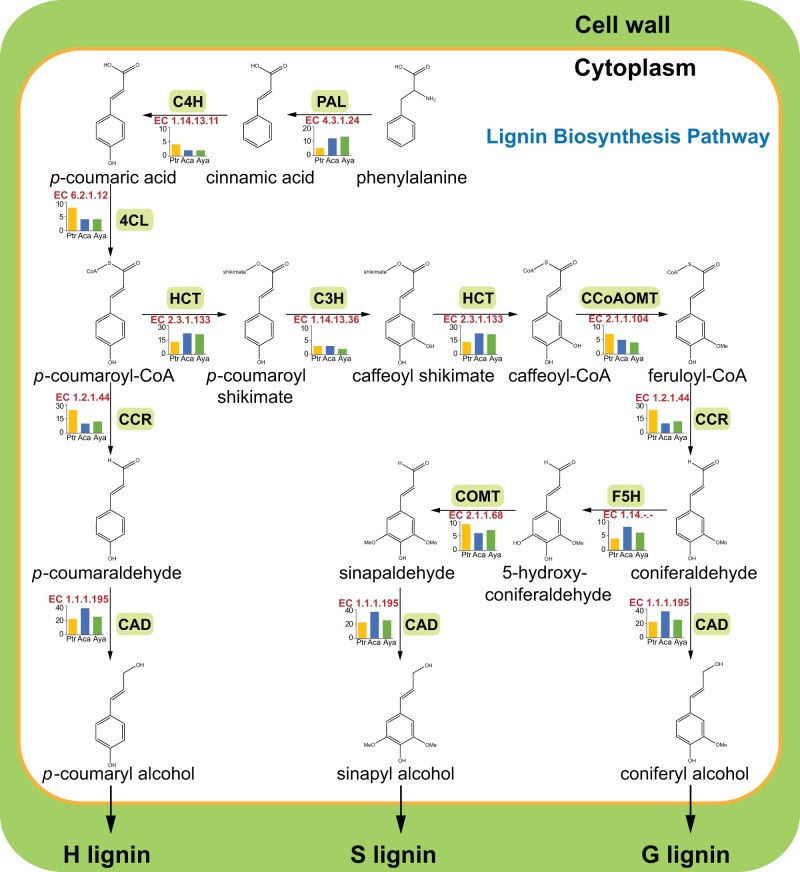
Comparison of the copy number of genes encoding ten key enzymes involved in lignin biosynthesis. In every reaction depicted, the name above the arrow represents the short name of the enzyme, and the histogram beneath the arrow represents the copy number of genes encoding the enzyme.

A previous study in poplar showed that low-lignin wood has reduced strength and stiffness (Voelker et al. 2011). Therefore, we investigated the genetic basis of lignin biosynthesis in *P. trichocarpa* and *A. catalpifolium*, with the aim of understanding why these two trees have different wood traits. Following a previous study on lignin biosynthesis (Vanholme et al. 2019), we used the genome sequence of *A. catalpifolium* together with *P. trichocarpa* and *A. yangbiense* to investigate the genes encoding ten key enzymes in the lignin biosynthesis pathway (fig. 6). The Ensemble Enzyme Prediction Pipeline (E2P2) was used to predict lignin biosynthesis pathway genes in the three plants (Schlapfer et al. 2017). A total of 109 genes were identified in the *A. catalpifolium* genome as participating directly in lignin biosynthesis (supplementary fig. S15 and table S13, Supplementary Material online), including 12 phenylalanine ammonia-lyase (PAL) genes, two cinnamate 4-hydroxylase (C4H) genes, four 4-coumarate: CoA ligase (4CL) genes, ten cinnamoyl-CoA reductase (CCR) genes, 37 cinnamyl alcohol dehydrogenase (CAD) genes, 22 p-hydroxycinnamoyl-CoA: quinate/shikimate (HCT) genes, three p-coumarate 3-hydroxylase (C3H) genes, five caffeoyl-CoA o-methyltransferase (CCoAOMT) genes, eight ferulate 5-hydroxylase (F5H) genes, and six caffeic acid o-methyltransferase (COMT) genes. There were 99 such genes in *P. trichocarpa* and 96 in *A. yangbiense* (supplementary tables S14 and S15, Supplementary Material online).

Notably, eight CAD genes in *A. catalpifolium* were found in the significantly expanded orthogroups, whereas only two 4CL genes in *P. trichocarpa* were found in the significantly expanded orthogroups, and no gene was found in *A. yangbiense*. The expansion of CAD family may contribute to the higher lignin concentration in *A. catalpifolium* compared with *P. trichocarpa*. Because lignin performs important roles in stem integrity, hydraulic conductivity, and biotic/abiotic defenses, altering both lignin content and syringyl/guaiacyl (S/G) composition are important for the adaptation of trees to the environment (Porth et al. 2013). The predicted genes encoding F5H and COMT enzymes in *A. catalpifolium* make the modulation of the S/G ratio possible (Boerjan et al. 2003), which may further improve the adaptation ability of *A. catalpifolium*. In addition, these findings provide an opportunity to improve wood traits with lignin engineering.

## Discussion

*A.* *catalpifolium* is endangered, but because genomic information was previously unavailable, conservation efforts for this tree have been limited to traditional methods such as natural interventions (Song and Liu 2020). In this study, we generated a high-quality genome of *A. catalpifolium*, thus accelerating future research and aiding in conservation of this tree. With the genomes of two endangered *Acer* species now available, we estimated their evolutionary divergence using two methods. One method was construction of a phylogenetic tree with estimated branch lengths and some fossil evidence for calibration. The other method was to utilize the formula λ = *Ks*/2T to estimate divergence time, supposing that *A. catalpifolium*, *A. yangbiense*, and *V. vinifera* shared the same mutation rate with their common ancestor, which experienced the gamma WGD event. In our study, the second method estimated a younger divergence time (5.2–5.9 Ma) than the first (6.9 Ma), which means these two methods were based on different rates of nucleotide substitution.

Previously, researchers found that a biodiversity hotspot in the Hengduan Mountains region has uplifted in the last eight million years (Xing and Ree 2017), which might have facilitated the divergence of *A. catalpifolium* and *A. yangbiense* and their speciation. Given that both *A. catalpifolium* and *A. yangbiense* are endangered, we reconstructed the demographic history of *A. catalpifolium* and *A. yangbiense* using a pairwise sequentially Markovian coalescent (PSMC) model (Li and Durbin 2011), and found that populations of *A. catalpifolium* and *A. yangbiense* show roughly the same trend: decreasing steadily after the Last Glacial Maximum (LGM) (supplementary fig. S16, Supplementary Material online). More information such as survival rate, climate change data, and plant–environment interactions should be included in a population fluctuation simulation. Furthermore, studies on population genomics and Genome-Wide Association Study (GWAS) are needed to investigate the genetic structure and specific genetic variations of *A. catalpifolium*, which may help to identify key factors that affect the effective population size.

The PSMC result is partially confirmed by our identification of PSGs in both *A. catalpifolium* and *A. yangbiense*, some of which had positively selected sites in the protein domain and led to altered 3D protein structure. Previous research has shown that abnormal expression of *AT1G54330.1* (orthologous gene of the PSG *EVM0008503.1*) caused severe root growth defects and discontinuous SE differentiation in roots (Kondo et al. 2016). We hypothesize that protein structural changes would affect biological functions of some PSGs in both trees. This might have adverse impacts on tree growth and development, affecting the survival of the endangered *A. catalpifolium* and *A. yangbiense*. We additionally performed GO enrichment analysis on genes in the 41 orthogroups that were significantly contracted in *A. catalpifolium*. Notably, they were highly enriched in catalytic activity and binding functions, such as “organic phosphonate transmembrane-transporting ATPase activity” (*P* value = 5.71E−08) and “transition metal ion binding” (*P* value = 2.31E−05) (supplementary fig. S17, Supplementary Material online). This suggests that some genes related to basic molecular functions were lost after the divergence of the two *Acer* trees, which might also contribute to the declining populations of *A. catalpifolium*. Further genetic and transgenic approaches are needed to experimentally test these hypotheses.

*A.* *catalpifolium* is located in the rainy zone of western China (Yu et al. 2020). As part of the Hengduan Mountains, this region has a special climate and unique vegetation types, with perennial rain and insufficient sunshine duration (Zhuang and Gao 2002). Previous studies have shown that low illumination intensity has adverse effects on *A. catalpifolium* sapling growth (Zhang et al. 2018). Because FRS genes play an important role in shade-avoidance responses, the significant expansion of the FRS–FRF gene family in *A. catalpifolium* might have helped the tree to adapt to the rainy zone of western China. Therefore, experiments such as overexpressing specific FRS genes are needed to test whether these genes have the potential to raise the survival rate of *A. catalpifolium*, which could save the tree from extinction. Future studies could combine mutant phenotype analysis with transcriptomic analysis to identify additional genes that are involved in important biological processes such as seed germination, photosynthetic efficiency, lignin content engineering, and biotic/abiotic stress responses. With the genome of *A. catalpifolium* now available, biological methods such as CRISPR-Cas9 genome editing (Chen et al. 2019) can be used to transplant novel genes into *A. catalpifolium* that may improve the tree’s capacity for adaptation.

*A.* *catalpifolium* exhibits no further lineage-specific WGD events after the well-acknowledged gamma event shared by core eudicots (Jaillon et al. 2007). WGD events may serve as important factors in improving adaptive evolution by creating a large amount of raw genetic material (Van De Peer et al. 2017). Therefore, we hypothesized that the extremely small population of *A. catalpifolium* could be, at least in part, associated with the lack of recent WGDs. Previous studies have shown that synthesized allohexaploid wheat displays polyploid growth vigor and adaptation from cytological, genetic, and epigenetic perspectives (Li et al. 2015). Other synthesized allopolyploids also go through rapid genomic reorganization after WGD, like *Brassica* and *Tragopogon* (Song and Chen 2015). Because polyploidy is a major force in shaping plant biodiversity, it is possible to acquire genetic novelty for environmental adaptation by synthesizing allopolyploids or autopolyploids using *A. catalpifolium* as the progenitor (Leitch and Leitch 2008).

## Materials and Methods

### Plant Materials and DNA Extraction

*Acer* *catalpifolium* seeds were collected from a 20-year-old tree grown in the West China Subalpine Botanical Garden, Dujiangyan, Sichuan Province (31°00′33″N, 103°37′00″E). In 2017, seeds were germinated in pots filled with organic substrate and silica sand. Seedlings were transplanted in the Beijing Forestry University climate chamber with constant 60% moisture, 20 °C air temperature, and 12 h light supply. Total genomic DNA was extracted from fresh leaves using the CTAB method (Doyle and Doyle 1987).

### Genome Sequencing

We used three platforms for DNA sequencing: Illumina, PacBio, and Hi-C. The Illumina Hiseq X Ten platform was used for 150 bp paired-end sequencing with average library insert sizes of 350 bp, generating 34.40 Gb reads. The library preparation was performed using ultrasonication to fragment DNA sequences, then DNA polymerase was added to repair fragmented DNA into dsDNA at the flat terminal. T4 polynucleotide kinase phosphorylated the 5′ terminal, and a poly-A tail was added to the 3′ end of dsDNA. SMRT libraries were constructed following the Quail protocol (Quail et al. 2012). Libraries were sequenced using the PacBio Sequel platform and 34.39 Gb reads were generated. Hi-C libraries were prepared according to the following steps: 1) formaldehyde was used to fix samples, cross-linking intracellular proteins, and DNA to preserve their interaction relationships. 2) Restriction enzymes were used to cut DNA to produce sticky ends on both sides of cross-linking. 3) Biotin-labeled bases were introduced to facilitate subsequent DNA purification. 4) DNA fragments cross-linked with proteins were amplified by PCR. 5) DNA was cross-linked, purified, and broken into 300–700 bp fragments. Strand affinity magnetic beads were used to capture and remove DNA fragments cross-linked with proteins from downstream sequencing.

### RNA Extraction and Sequencing

Total RNA was extracted from the leaf, stem, and root tissues of *A. catalpifolium* using the modified CTAB method (Yang et al. 2008). RNA quality was determined by estimation of the ratio of absorbance at 260 nm/280 nm. Sequencing libraries were prepared according to the following steps: 1) mRNA was enriched with VAHTS mRNA-seq V3 Library Prep Kit for Illumina (Vazyme). 2) mRNA was randomly interrupted using fragmentation buffer. 3) Using mRNA as the template, the first cDNA strand was synthesized with random hexamers. The buffer, dNTPs, RNase H, and DNA Polymerase I were then added to synthesize the second cDNA strand. 4) AHTS DNA Clean Beads (Vazyme) were used to purify cDNA. 5) The cDNA library was obtained through PCR amplification. The paired-end sequencing libraries were sequenced on an Illumina HiSeq X Ten platform.

### De Novo Genome Assembly

After removing low quality reads, subreads from PacBio were assembled using a combination of Canu (v1.5, corOutCoverage = 50) (Koren et al. 2017), WTDBG (“–k 21 –S 1.02 –e 3”, https://github.com/ruanjue/wtdbg, last accessed December 13, 2021), Falcon (v0.3.0, length_cutoff_pr = 8000) (Chin et al. 2016), DBG2OLC (“KmerCovTh 2 MinOverlap 25 AdaptiveTh 0.008 k 17”) (Ye et al. 2016), and Quickmerge (“–hco 5.0 –c 1.5 –l 100000 –ml 5000”) (Chakraborty et al. 2016). The contigs of the assembly were corrected with Illumina reads using Pilon v1.22 (“–mindepth 10 –changes –threads 4 –fix bases”) (Walker et al. 2014). The polished contigs were reassembled to form scaffolds with the help of Hi-C reads using LACHESIS with default parameters (Burton et al. 2013). The gaps were then filled with PacBio long reads using PBjelly with default parameters (English et al. 2012). The final assembly was constructed after correcting with Illumina short reads using Pilon. Genome completeness was assessed and estimated with BUSCO v2.0 (Simao et al. 2015).

### Gene Predictions and Annotations

We used three different strategies to identify genes in *A. catalpifolium*. First, genes were predicted de novo from DNA sequences with ab initio approaches using four programs with default settings, namely Genscan v3.1 (Burge and Karlin 1997), Augustus v3.1 (Stanke and Waack 2003), GlimmerHMM v1.2 (Majoros et al. 2004), and SNAP v2006-07-28 (Korf 2004). GeMoMa v1.3.1 (Keilwagen et al. 2016) was used with default parameters to predict new genes based on sequence similarity with *Arabidopsis*, *Glycine max*, *P. trichocarpa*, *V. vinifera*, *Prunus persica*, and *S. lycopersicum*. RNA-seq data were processed with PASA v2.0.2 (Campbell et al. 2006), TransDecoder v2.0, and GeneMarkS-T v5.1 (Tang et al. 2015) separately to predict gene sequences. All of the processing programs were executed with default parameters. Finally, all assembled genes identified by any of the three methods described above were combined using EVM v1.1.1 (Haas et al. 2008) with the following parameters: “Mode: STANDARD S-ratio: 1.13 score > 1000.” Amino acid sequences of the predicted gene models were searched against different databases, namely TrEMBL (Apweiler et al. 2004), SwissProt (Apweiler et al. 2004), Pfam (Mistry et al. 2021), NR/NT (Pruitt et al. 2007), KOG/KEGG (Kanehisa et al. 2016), and GO (Ashburner et al. 2000) using BLASTP v2.2.31 (−E 1e−5) for functional annotation.

### Genome Synteny and WGD Analysis

The proteomes of *A. yangbiense* and *V. vinifera* were downloaded from public resources, namely GigaDB (Sneddon et al. 2012) and Phytozome v12 (Goodstein et al. 2012). Sequence similarity was analyzed between the three species (*A. catalpifolium*, *A. yangbiense*, and *V. vinifera*) using BLASTP (−E 1e−10). MCScanX (Wang et al. 2012) was then used with default settings to identify syntenic blocks and colinear gene pairs. To obtain the best “one-to-one” syntenic matchings, we eliminated colinear blocks with fewer than nine genes, then eliminated gene pairs without macrosynteny information support. Finally, the gene in a block containing the largest number of genes was retained. The proteins of those homologous gene pairs were first aligned using MUSCLE v3.8.31 (Edgar 2004) with default parameters, then converted to CDS alignment using PAL2NAL v14 (Suyama et al. 2006) with default parameters. The *Ks* values were calculated with custom scripts using the Yang–Nielsen (YN) model in PAML (Yang 2007).

### Orthogroup Inference

We selected 13 representative plants including *A. catalpifolium* to determine orthogroups. Protein sequences were downloaded from two sources: *A. yangbiense* from GigaDB (Sneddon et al. 2012) and the others from Phytozome v12 (Goodstein et al. 2012). We separately ran all-versus-all protein sequence alignments in these species using BLASTP (−E 1e−5). Orthogroups were generated by OrthoMCL v14.137 (Li et al. 2003) with default parameters using the BLASTP results.

### Phylogenetic Analysis and Divergence Time Estimation

We selected 873 single-copy orthogroups from 13 plant species, and amino acid sequences of each orthogroup were included in a multiple sequence alignment using MAFFT v7.427 (Katoh et al. 2002) with default parameters. The aligned protein sequences were transformed into codon alignments using PAL2NAL v14 with default parameters. The codon alignments were concatenated as a supermatrix and the tree was inferred using RAxML (v8.2.12; parameters: –f a –m GTRGAMMA) with 1,000 bootstrap replicates (Stamatakis et al. 2005). The resulting species tree was used as input to infer the divergence time of 13 plants using r8s v1.81 (Sanderson 2003) with default settings except for setting four fossil records at the key nodes of the tree: “mrca Origin_rosids Acat Vvin; mrca Crown_Eudicots Acoe Acat; mrca Crown_Monocots Macu Osat; mrca Crown_Angiosperms Atri Macu; constrain taxon=Origin_rosids max_age=125; constrain taxon=Crown_Eudicots min_age=125; constrain taxon=Crown_Monocots min_age=113; constrain taxon=Crown_Angiosperms min_age=132.9 max_age=260.” These fossil calibrations were set with reference to one published paper with minor modifications (Li et al. 2019). The other method to infer the timing of divergence between two *Acer* trees was based on the assumption of sharing the same λ (global mutation rate), meaning λ = *Ks*_1_/2T_1_ = *Ks*_2_/2T_2_.

### Calculation of *ω* Value (*K*a/*K*s Ratio)

The 13 aforementioned angiosperms were used to calculate *ω*, assuming that each branch of the phylogenetic tree has a different *ω* (Xu et al. 2017). The 873 single-copy orthogroups from the 13 species were aligned using MUSCLE (default parameters) and converted into codon alignments with PAL2NAL (default parameters). The alignments of the 873 single-copy orthogroups were then concatenated into a super matrix and the “free-ratio” model of CODEML in the PAML 4.9j package (Yang 2007) was used to calculate *ω* for each branch of the phylogenetic tree constructed above.

Next, we used six eudicots (*A. catalpifolium*, *A. yangbiense*, *Arabidopsis*, *C. sinensis*, *D. longan*, and *P. trichocarpa*) to calculate *ω*. Orthogroups from the six species were identified using OrthoMCL (Li et al. 2003) with default parameters. Orthogroups with a single copy in each of the six plants were identified, as were orthogroups with one single copy in *Arabidopsis* (the outgroup) but multiple copies in the other five species. We aligned single copy *Arabidopsis* genes (using BLASTP) against those multiple copies from other five species to generate best hits as representative orthologs of each plant. Ultimately, we found 6,116 orthogroups with one copy of one plant in each (Xu et al. 2017).

The 6,116 codon alignments were also concatenated into a supermatrix and the “free-ratio” model was used to calculate *ω* in each branch of the phylogenetic tree. Alignments of the 6,116 orthogroups were used as input to calculate *ω* separately using the “branch” model in PAML, hypothesizing that the *Acer* branch has a different *ω* from the remaining branches. The likelihood ratio test (LRT) (Anisimova et al. 2001) was used to remove false positive results (*P* value <0.05). Next, the alignments of the 6,116 orthogroups were analyzed using the “branch-site” model in CODEML to detect PSGs (“fix_omega = 1, omega = 1” for the null model, “fix_omega = 0, omega = 2” for the alternative model). Setting the branch of *Acer* genus as the foreground, the likelihood of the null model (no site in the *Acer* clade is positively selected) and alternative model (sites exist in the *Acer* clade that are positively selected) were calculated. The LRT was used to remove false findings and genes under positive selection (*ω *> 1, *P* value <0.05) using the Bayes Empirical Bayes method were further analyzed (Yang et al. 2005). Furthermore, setting the node of *A. catalpifolium* or *A. yangbiense* as the foreground, the likelihood of the null model (no site in *A. catalpifolium* or *A. yangbiense* is positively selected) and alternative model (sites exist in *A. catalpifolium* or *A. yangbiense* that are positively selected) were also calculated to identify differentially selected genes. Orthogroups were annotated to different TFs based on their annotations in *Arabidopsis* in PlantTFDB 5.0 (Tian et al. 2020).

### Gene Family Evolution

We used the ultrametric tree identified by r8s v1.81 and orthogroups inferred from 13 plant genomes as input to compute the expansion and contraction of gene families. The program Count (Csuros 2010) was implemented using a Wagner parsimony framework (gain penalty = 1.2, which produced the most reasonable number of expanded/contracted gene families after testing with other values) to understand the expanded/contracted gene families along each branch of the ultrametric tree. CAFE v4.2.1 (De Bie et al. 2006) with default parameters was used to identify rapidly evolving gene families.

### GO Enrichment Analysis

The R package clusterProfiler v3.12 (pvalueCutoff = 0.05, pAdjustMethod = “BH,” qvalueCutoff = 0.05) was used to perform GO enrichment analysis and identify statistically enriched GO terms (Yu et al. 2012). The genes in significantly expanded or *Acer* genus-specific orthogroups were set as the foreground and the genes annotated with GO terms at the whole-genome level were regarded as the background. The adjusted *P* values were calculated to obtain significantly enriched GO terms using the Benjamini and Hochberg method (adjusted *P* value <0.05).

### FRS and FRF Gene Family

FRS genes were identified using a combination of several methods. First, protein sequences of *A. catalpifolium* and *A. yangbiense* were searched against three Pfam protein families: FAR1 domain (PF03101), MULE domain (PF10551), and SWIM domain (PF04434) using HMMER (v3.3, parameters: –E 0.01 –domE 0.01) (Mistry et al. 2013). Then, extracted sequences containing none of those domains were filtered to remove any false-positive domains using InterproScan v5.39-77.0 (Quevillon et al. 2005). The genes that passed both HMMER and InterproScan were retained for downstream analyses, and the proteins containing all three domains were regarded as the most conserved FRS members. Next, BLASTP was used against the combined database of 18 FRS and FRF proteins in *Arabidopsis* and the most conserved FRS genes in *A. catalpifolium* and *A. yangbiense* to obtain additional putative FRS candidates (−E 1e−10). False positive candidate proteins were removed by manually inspecting multiple sequence alignments, and the proteins that had conserved amino acid residues in both MULE and SWIM domains were retained (Lin and Wang 2004). Proteins that had only the FAR1 domain were regarded as FRF genes. The protein sequences for FRS and FRF genes from three species (*Arabidopsis*, *A. catalpifolium*, and *A. yangbiense*) were aligned using MAFFT v7.427 (with default parameters) and put into RAxML (v8.2.12; parameters: –f a –m PROTGAMMAAUTO -# 1000) to construct a phylogenetic tree of the FRS and FRF gene family. FRS and FRF gene locations were integrated with MCScanX results and repetitive sequence annotation to determine the number of genes derived from different types of gene duplication.

### Lignin Biosynthesis

The lignin biosynthesis pathway was built based on information of PoplarCyc in Plant Metabolic Network (PMN) (Caspi et al. 2016) and a previous study (Vanholme et al. 2019). These key enzymes that participate in the pathway were predicted with the E2P2 algorithm v3.0 designed by PMN using proteomes of *A. catalpifolium* and *A. yangbiense* separately (with default parameters). The candidate genes in the two species were annotated based on the EC (Enzyme Commission) number of the enzyme.

### PSMC Analysis

We inferred a demographic history of *A. catalpifolium* and *A. yangbiense* by applying the PSMC model to the complete diploid genome sequences. This method reconstructs the history of dynamic changes in population size over time using the distribution of the most recent common ancestor between two alleles in one individual. Paired-end Illumina reads of *A. catalpifolium* and *A. yangbiense* were aligned to their reference genomes using BWA-MEM v0.7.10 (Peters et al. 2011). Then, the heterozygous biallelic SNPs were called using SAMtools v0.1.19 (Li et al. 2009), and reads of very low depth (less than a third of the average depth) or of very high depth (twice the average depth) were removed. Bcftools v1.1 (Li et al. 2009) was used to convert the BCF file into VCF format, and vcfutils.pl (https://github.com/lh3/samtools/blob/master/bcftools/vcfutils.pl, last accessed December 13, 2021) was used to convert the VCF file into the whole-genome diploid consensus sequence. All of the parameters utilized in the PSMC program were set to default with the exception of a per-generation mutation rate (μ) of 7.5 × 10^−9^ taken from *Arabidopsis* (Buschiazzo et al. 2012) and 30 years per *Acer* generation (g) taken from data for *A. mono* (Liu et al. 2014).

## Supplementary Material

Supplementary data are available at *Genome Biology and Evolution* online.

## Supplementary Material

evab271_Supplementary_Data
