# Supplementary material for: Whole-Genome Sequencing of Acer catalpifolium Reveals Evolutionary History of Endangered Species
Source: Genome Biol Evol. 2021 Dec 8;13(12):evab271. doi: 10.1093/gbe/evab271 (PMC8677443; doi:10.1093/gbe/evab271)
Supplement: evab271_Supplementary_Data [file evab271_supplementary_data.pdf]

Whole-genome sequencing of *Acer catalpifolium* reveals evolutionary history of endangered species

SUPPLEMENTARY FIGURES AND TABLES

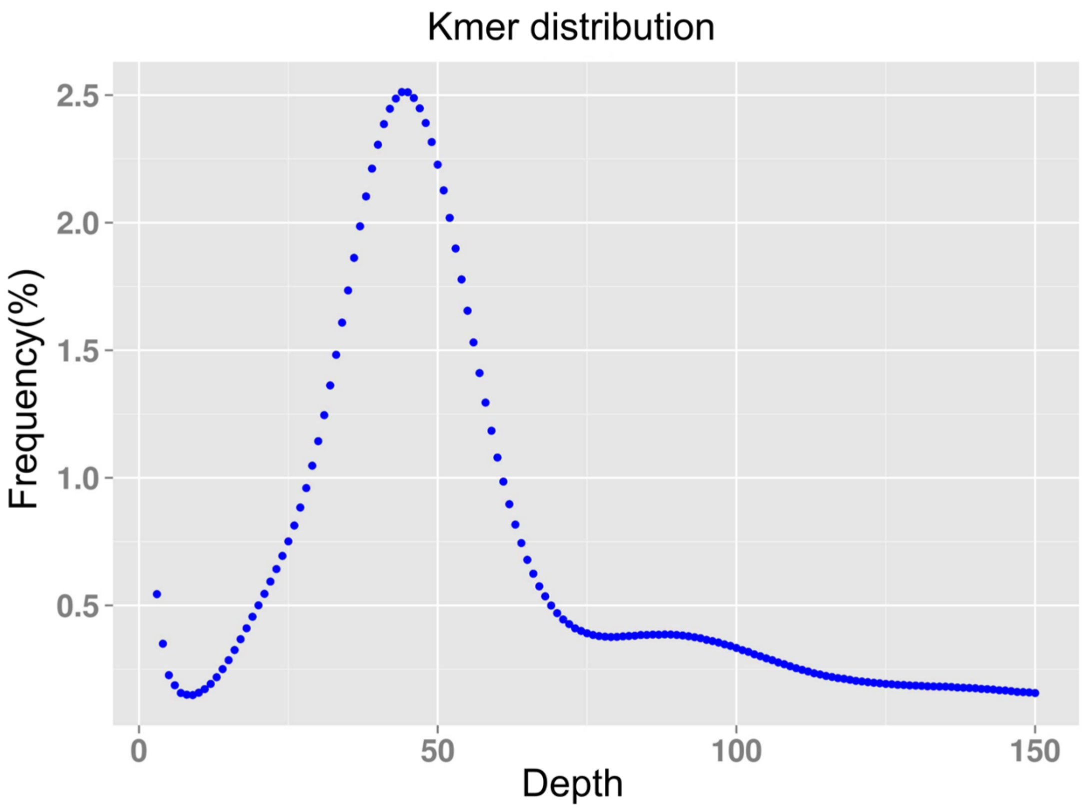

Figure S1. Distribution of k-mer frequencies in the *A. catalpifolium* genome.

The x-axis refers to the k-mer depth, while the y-axis refers to the frequency of the k-mer for a given depth.

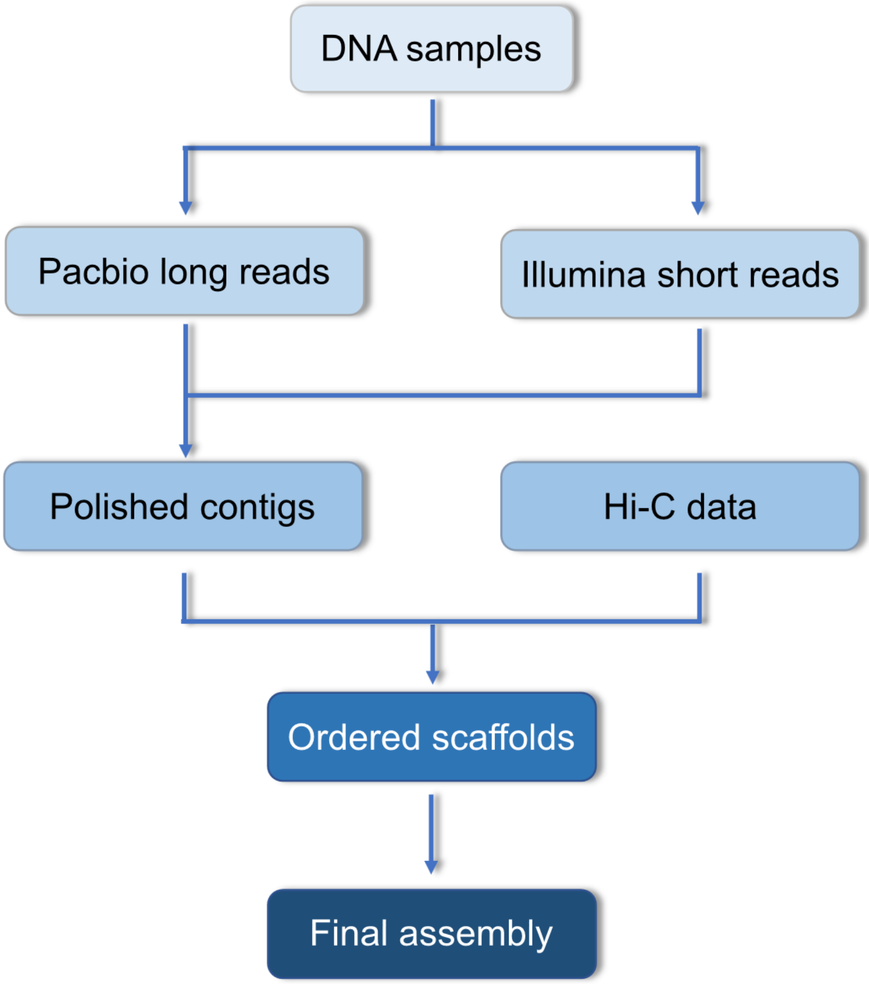

Figure S2. Annotation and assembly pipeline for the *A. catalpifolium* genome.

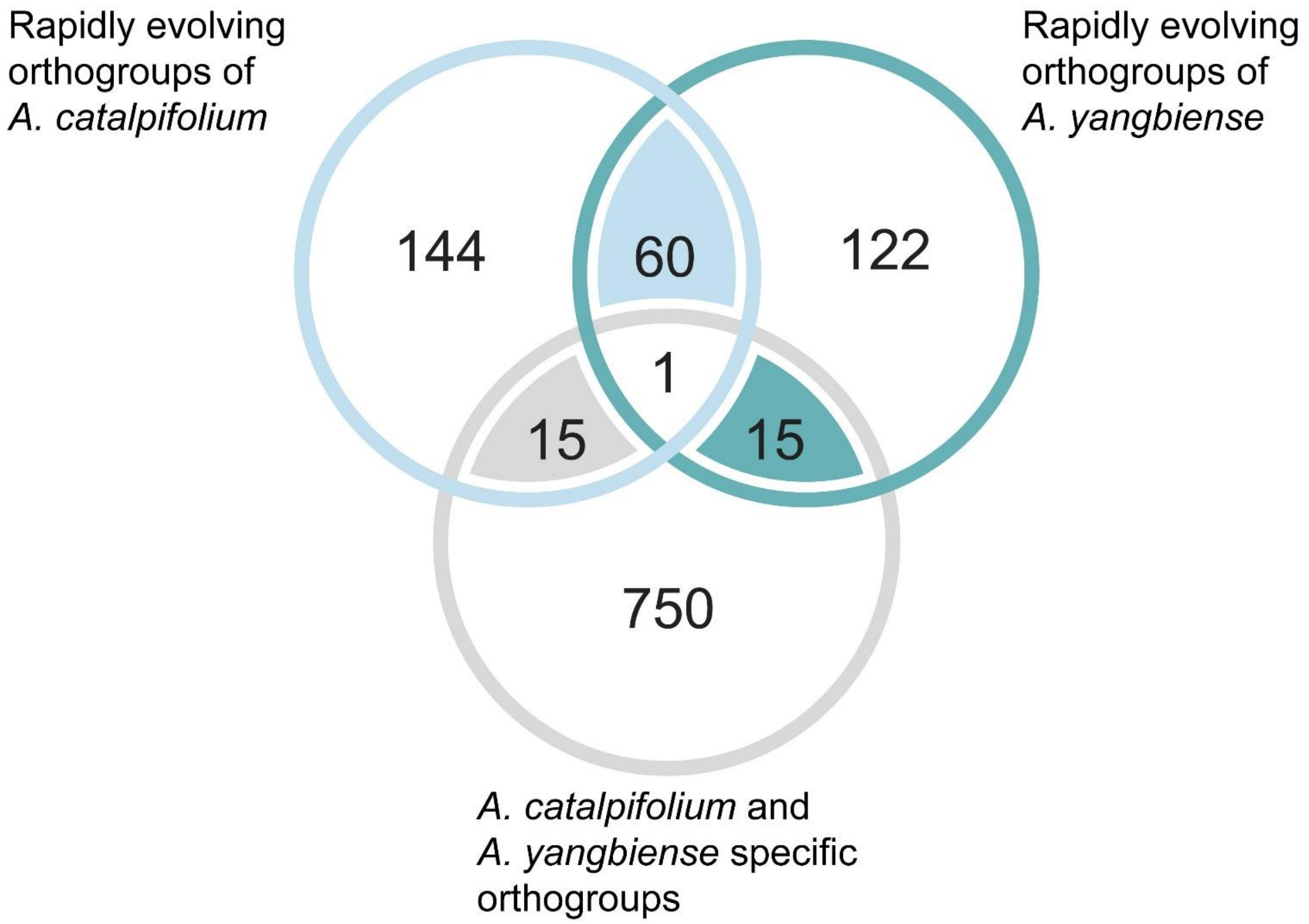

Figure S3. Overlap of rapidly evolving orthogroups in *A. catalpifolium* and *A. yangbiense* with *Acer* genus-specific orthogroups.

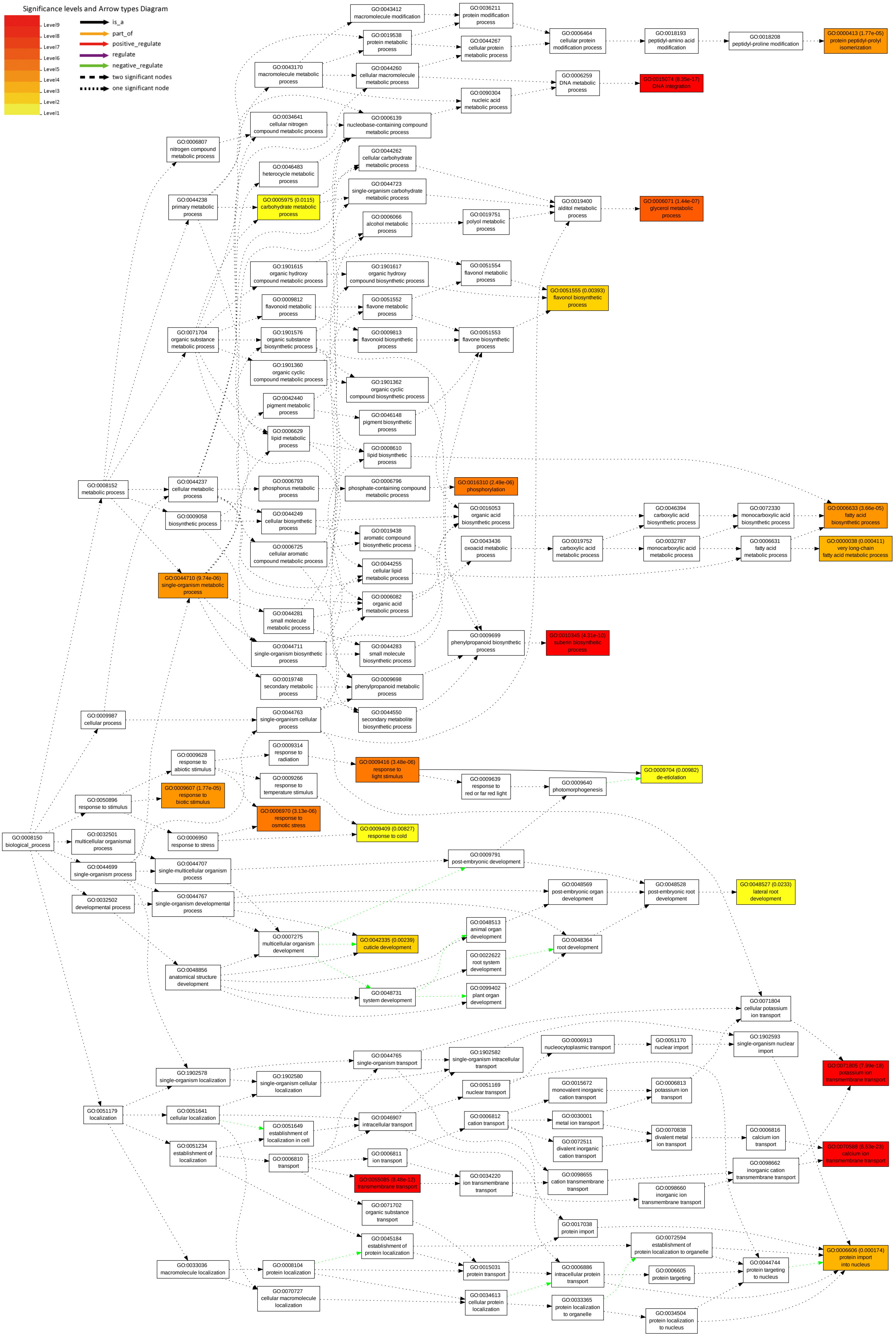

Significance levels and Arrow types Diagram
Level9
Level8
Level7
Level6
Level5
Level4
Level3
Level2
Level1
is_a
part_of
positive_regulate
regulate
negative_regulate
two significant nodes
one significant node
GO:0043412 macromolecule modification
GO:0036211 protein modification process
GO:0019538 protein metabolic process
GO:0044267 cellular protein metabolic process
GO:0006464 cellular protein modification process
GO:0018193 peptidyl-amino acid modification
GO:0018208 peptidyl-proline modification
GO:0000413 (1.77e-05) protein peptidyl-prolyl isomerization
GO:0043170 macromolecule metabolic process
GO:0044260 cellular macromolecule metabolic process
GO:0006259 DNA metabolic process
GO:0015074 (8.35e-17) DNA integration
GO:0090304 nucleic acid metabolic process
GO:0034641 cellular nitrogen compound metabolic process
GO:0006139 nucleobase-containing compound metabolic process
GO:0006807 nitrogen compound metabolic process
GO:0046483 heterocycle metabolic process
GO:0044262 cellular carbohydrate metabolic process
GO:0044238 primary metabolic process
GO:0005975 (0.0115) carbohydrate metabolic process
GO:0044723 single-organism carbohydrate metabolic process
GO:0019400 alditol metabolic process
GO:0006071 (1.44e-07) glycerol metabolic process
GO:0006066 alcohol metabolic process
GO:0019751 polyol metabolic process
GO:1901615 organic hydroxy compound metabolic process
GO:1901617 organic hydroxy compound biosynthetic process
GO:0051554 flavonol metabolic process
GO:0051555 (0.00393) flavonol biosynthetic process
GO:0009812 flavonoid metabolic process
GO:0051552 flavone metabolic process
GO:0071704 organic substance metabolic process
GO:1901576 organic substance biosynthetic process
GO:0009813 flavonoid biosynthetic process
GO:0051553 flavone biosynthetic process
GO:1901360 organic cyclic compound metabolic process
GO:1901362 organic cyclic compound biosynthetic process
GO:0042440 pigment metabolic process
GO:0046148 pigment biosynthetic process
GO:0006629 lipid metabolic process
GO:0008610 lipid biosynthetic process
GO:0008152 metabolic process
GO:0044237 cellular metabolic process
GO:0006793 phosphorus metabolic process
GO:0006796 phosphate-containing compound metabolic process
GO:0016310 (2.49e-06) phosphorylation
GO:0009058 biosynthetic process
GO:0044249 cellular biosynthetic process
GO:0016053 organic acid biosynthetic process
GO:0046394 carboxylic acid biosynthetic process
GO:0072330 monocarboxylic acid biosynthetic process
GO:0006633 (3.66e-05) fatty acid biosynthetic process
GO:0019438 aromatic compound biosynthetic process
GO:0043436 oxoacid metabolic process
GO:0019752 carboxylic acid metabolic process
GO:0032787 monocarboxylic acid metabolic process
GO:0006631 fatty acid metabolic process
GO:0000038 (0.000411) very long-chain fatty acid metabolic process
GO:0006725 cellular aromatic compound metabolic process
GO:0044255 cellular lipid metabolic process
GO:0044710 (9.74e-06) single-organism metabolic process
GO:0006082 organic acid metabolic process
GO:0044281 small molecule metabolic process
GO:0009699 phenylpropanoid biosynthetic process
GO:0010345 (4.31e-10) suberin biosynthetic process
GO:0044711 single-organism biosynthetic process
GO:0044283 small molecule biosynthetic process
GO:0019748 secondary metabolic process
GO:0009698 phenylpropanoid metabolic process
GO:0009987 cellular process
GO:0044763 single-organism cellular process
GO:0044550 secondary metabolite biosynthetic process
GO:0009314 response to radiation
GO:0009628 response to abiotic stimulus
GO:0009416 (3.48e-06) response to light stimulus
GO:0009704 (0.00982) de-etiolation
GO:0009266 response to temperature stimulus
GO:0009639 response to red or far red light
GO:0009640 photomorphogenesis
GO:0050896 response to stimulus
GO:0009607 (1.77e-05) response to biotic stimulus
GO:0006970 (3.13e-06) response to osmotic stress
GO:0008150 biological_process
GO:0032501 multicellular organismal process
GO:0006950 response to stress
GO:0009409 (0.00827) response to cold
GO:0009791 post-embryonic development
GO:0044699 single-organism process
GO:0044707 single-multicellular organism process
GO:0048569 post-embryonic organ development
GO:0048528 post-embryonic root development
GO:0048527 (0.0233) lateral root development
GO:0032502 developmental process
GO:0044767 single-organism developmental process
GO:0048513 animal organ development
GO:0007275 multicellular organism development
GO:0042335 (0.00239) cuticle development
GO:0048364 root development
GO:0022622 root system development
GO:0048856 anatomical structure development
GO:0048731 system development
GO:0099402 plant organ development
GO:0071804 cellular potassium ion transport
GO:1902593 single-organism nuclear import
GO:0006913 nucleocytoplasmic transport
GO:0051170 nuclear import
GO:0044765 single-organism transport
GO:1902582 single-organism intracellular transport
GO:0071805 (7.99e-18) potassium ion transmembrane transport
GO:1902578 single-organism localization
GO:1902580 single-organism cellular localization
GO:0051169 nuclear transport
GO:0015672 monovalent inorganic cation transport
GO:0006813 potassium ion transport
GO:0051179 localization
GO:0051641 cellular localization
GO:0046907 intracellular transport
GO:0006812 cation transport
GO:0030001 metal ion transport
GO:0051649 establishment of localization in cell
GO:0070838 divalent metal ion transport
GO:0006816 calcium ion transport
GO:0051234 establishment of localization
GO:0006811 ion transport
GO:0072511 divalent inorganic cation transport
GO:0070588 (6.53e-23) calcium ion transmembrane transport
GO:0006810 transport
GO:0098662 inorganic cation transmembrane transport
GO:0055085 (8.48e-12) transmembrane transport
GO:0034220 ion transmembrane transport
GO:0098655 cation transmembrane transport
GO:0098660 inorganic ion transmembrane transport
GO:0071702 organic substance transport
GO:0017038 protein import
GO:0045184 establishment of protein localization
GO:0072594 establishment of protein localization to organelle
GO:0006606 (0.000174) protein import into nucleus
GO:0033036 macromolecule localization
GO:0008104 protein localization
GO:0015031 protein transport
GO:0006886 intracellular protein transport
GO:0006605 protein targeting
GO:0044744 protein targeting to nucleus
GO:0070727 cellular macromolecule localization
GO:0034613 cellular protein localization
GO:0033365 protein localization to organelle
GO:0034504 protein localization to nucleus

Figure S4. A direct acyclic graph (DAG) tree of biological processes (BP) aspect of significantly enriched GO terms with 2156 genes of *A. catalpifolium* from the 179 significantly expanded orthogroups as input.

GO terms with background color from yellow to red were significantly enriched, while others with white background were automatically supplemented by the DAG drawer from agriGO v2, showing the inter-relationships between enriched terms.

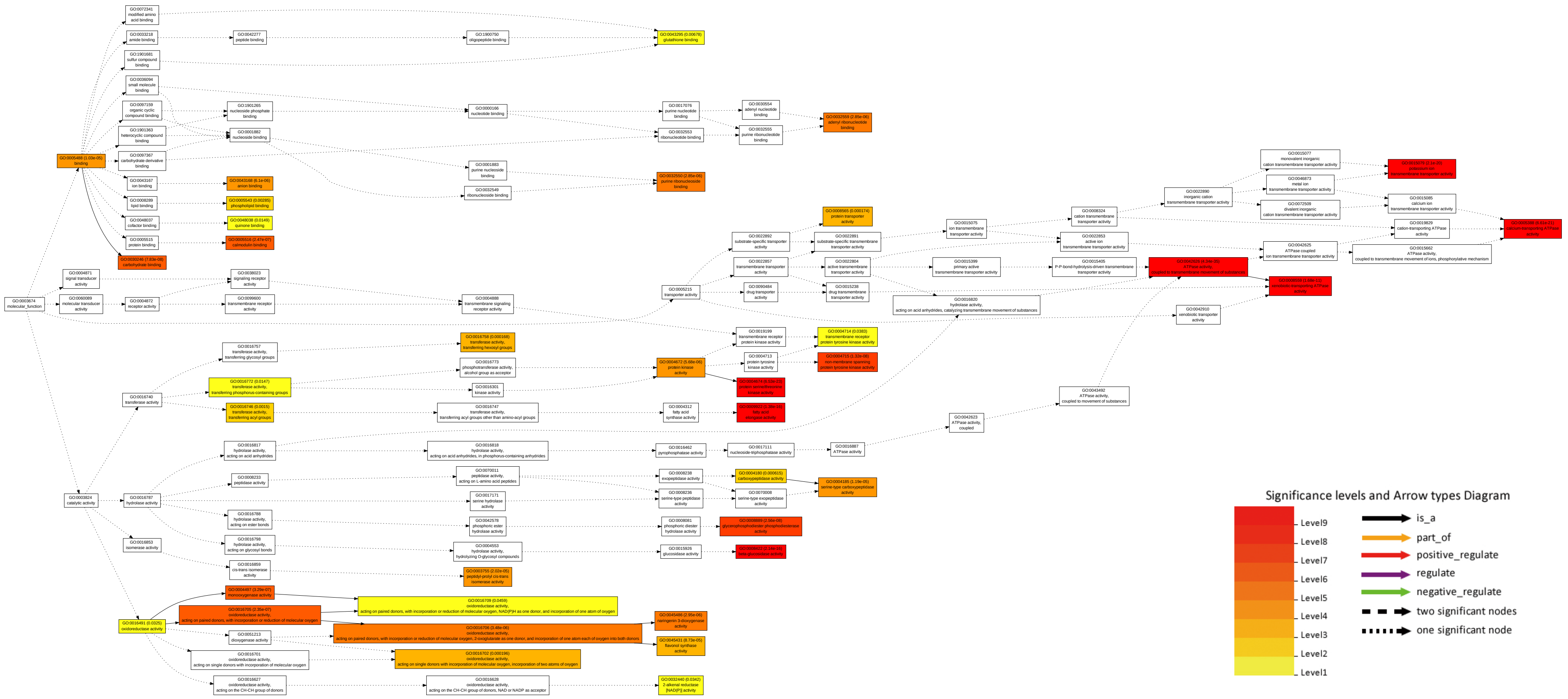

Figure S5. A direct acyclic graph (DAG) tree of molecular functions (MF) aspect of significantly enriched GO terms with 2156 genes of *A. catalpifolium* from the 179 significantly expanded orthogroups as input.

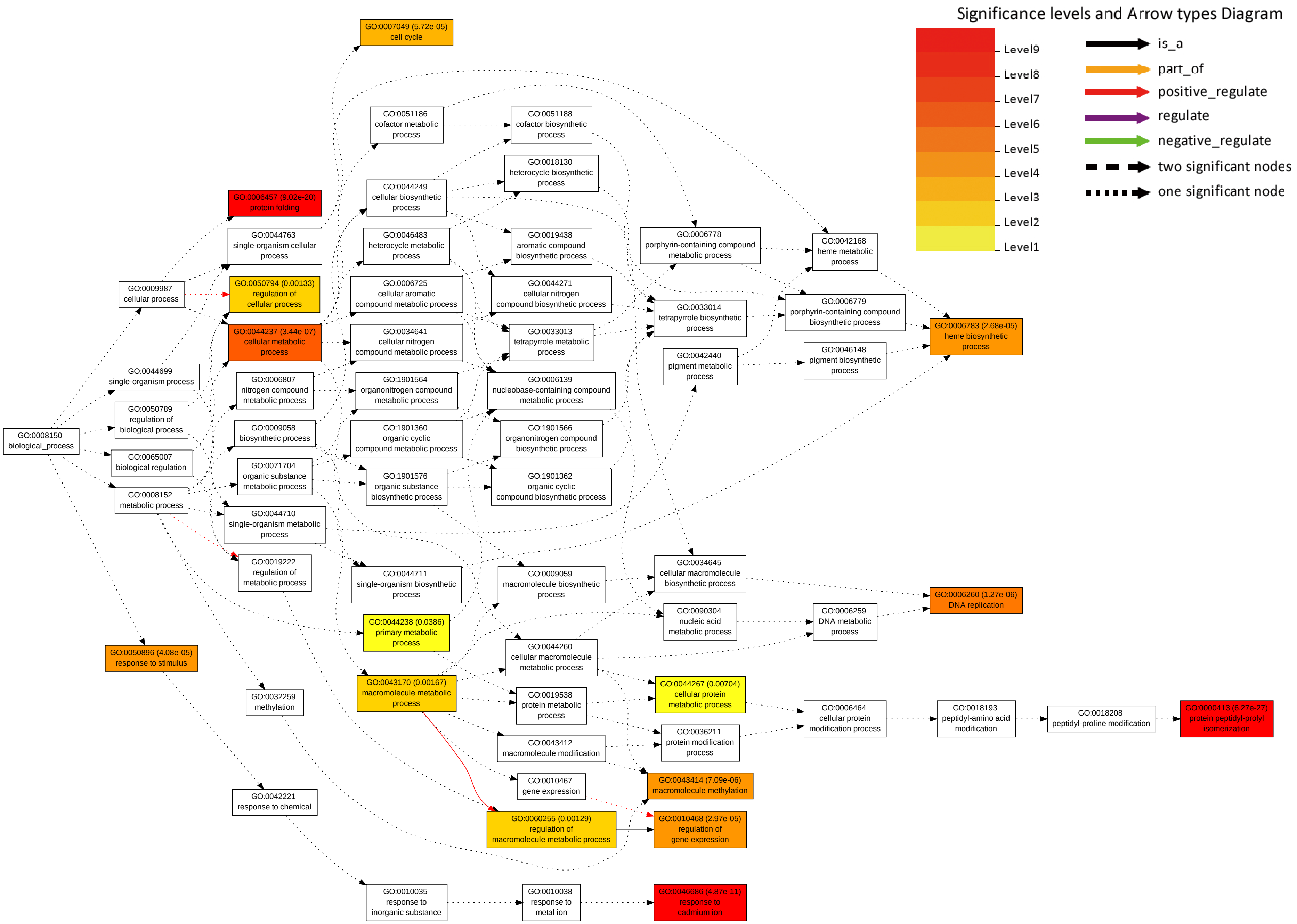
Significance levels and Arrow types Diagram
Level9
Level8
Level7
Level6
Level5
Level4
Level3
Level2
Level1
is_a
part_of
positive_regulate
regulate
negative_regulate
two significant nodes
one significant node
GO:0007049 (5.72e-05) cell cycle
GO:0051186 cofactor metabolic process
GO:0051188 cofactor biosynthetic process
GO:0018130 heterocycle biosynthetic process
GO:0006457 (9.02e-20) protein folding
GO:0044249 cellular biosynthetic process
GO:0044763 single-organism cellular process
GO:0046483 heterocycle metabolic process
GO:0019438 aromatic compound biosynthetic process
GO:0006778 porphyrin-containing compound metabolic process
GO:0042168 heme metabolic process
GO:0009987 cellular process
GO:0050794 (0.00133) regulation of cellular process
GO:0006725 cellular aromatic compound metabolic process
GO:0044271 cellular nitrogen compound biosynthetic process
GO:0033014 tetrapyrrole biosynthetic process
GO:0006779 porphyrin-containing compound biosynthetic process
GO:0006783 (2.68e-05) heme biosynthetic process
GO:0044237 (3.44e-07) cellular metabolic process
GO:0034641 cellular nitrogen compound metabolic process
GO:0033013 tetrapyrrole metabolic process
GO:0042440 pigment metabolic process
GO:0046148 pigment biosynthetic process
GO:0044699 single-organism process
GO:0006807 nitrogen compound metabolic process
GO:1901564 organonitrogen compound metabolic process
GO:0006139 nucleobase-containing compound metabolic process
GO:0050789 regulation of biological process
GO:0009058 biosynthetic process
GO:1901360 organic cyclic compound metabolic process
GO:1901566 organonitrogen compound biosynthetic process
GO:0008150 biological_process
GO:0065007 biological regulation
GO:0071704 organic substance metabolic process
GO:1901576 organic substance biosynthetic process
GO:1901362 organic cyclic compound biosynthetic process
GO:0008152 metabolic process
GO:0044710 single-organism metabolic process
GO:0019222 regulation of metabolic process
GO:0044711 single-organism biosynthetic process
GO:0009059 macromolecule biosynthetic process
GO:0034645 cellular macromolecule biosynthetic process
GO:0006260 (1.27e-06) DNA replication
GO:0090304 nucleic acid metabolic process
GO:0006259 DNA metabolic process
GO:0044238 (0.0386) primary metabolic process
GO:0050896 (4.08e-05) response to stimulus
GO:0044260 cellular macromolecule metabolic process
GO:0043170 (0.00167) macromolecule metabolic process
GO:0032259 methylation
GO:0019538 protein metabolic process
GO:0044267 (0.00704) cellular protein metabolic process
GO:0006464 cellular protein modification process
GO:0018193 peptidyl-amino acid modification
GO:0018208 peptidyl-proline modification
GO:0000413 (6.27e-27) protein peptidyl-prolyl isomerization
GO:0043412 macromolecule modification
GO:0036211 protein modification process
GO:0010467 gene expression
GO:0043414 (7.09e-06) macromolecule methylation
GO:0042221 response to chemical
GO:0060255 (0.00129) regulation of macromolecule metabolic process
GO:0010468 (2.97e-05) regulation of gene expression
GO:0010035 response to inorganic substance
GO:0010038 response to metal ion
GO:0046686 (4.87e-11) response to cadmium ion

Figure S6. A direct acyclic graph (DAG) tree of biological processes (BP) aspect of significantly enriched GO terms with 159 *Acer* genus-specific genes of *A. catalpifolium* from the significantly expanded orthogroups as input.

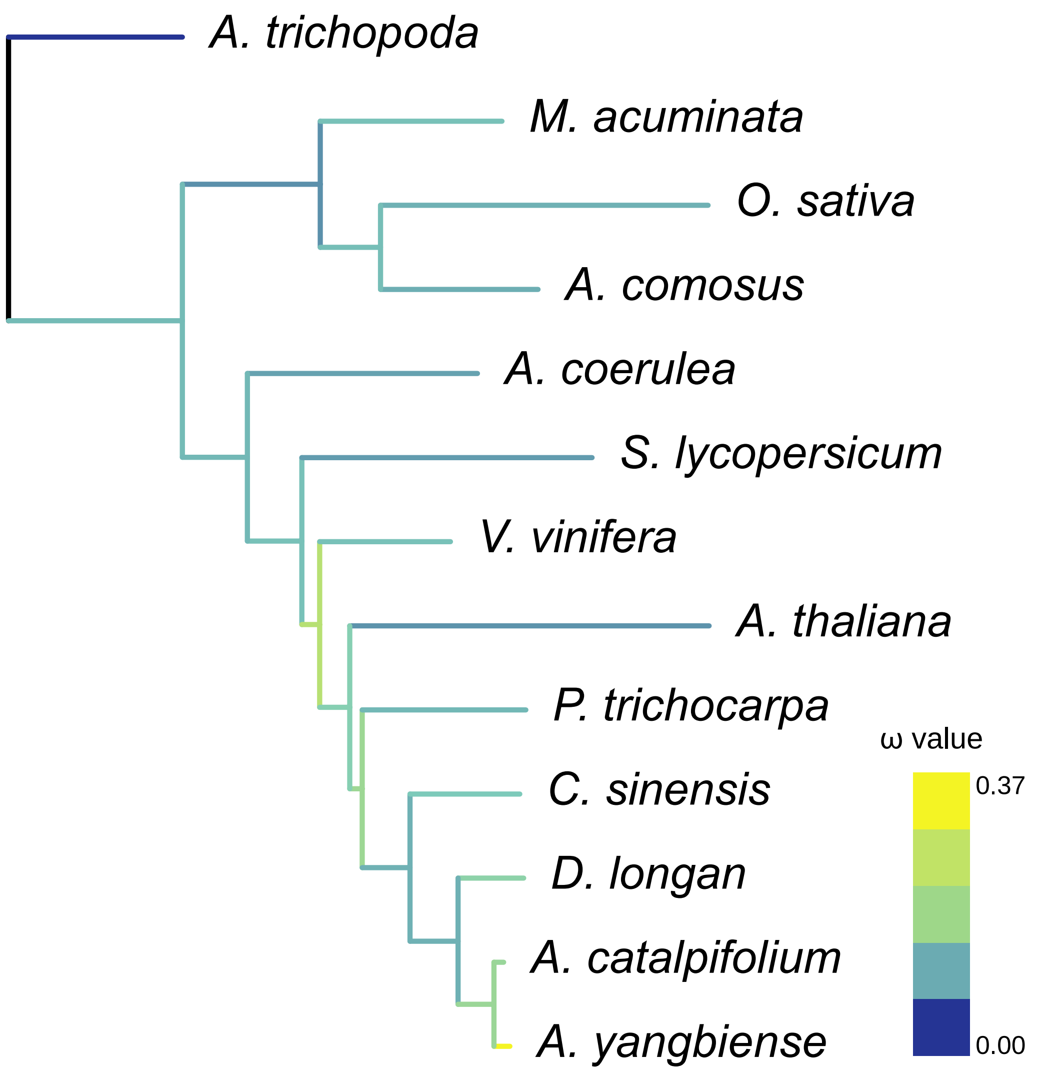

Figure S7. The ω ratios along each branch of the phylogenetic tree of 13 plants.

The *ω* ratios (Ka/Ks) were estimated at each branch of the phylogenetic tree using 13 representative plants including two *Acer* trees. The branches are colored according to the *ω* value in each branch. Blue represents the minimum *ω* value and yellow represents the maximum *ω* value.

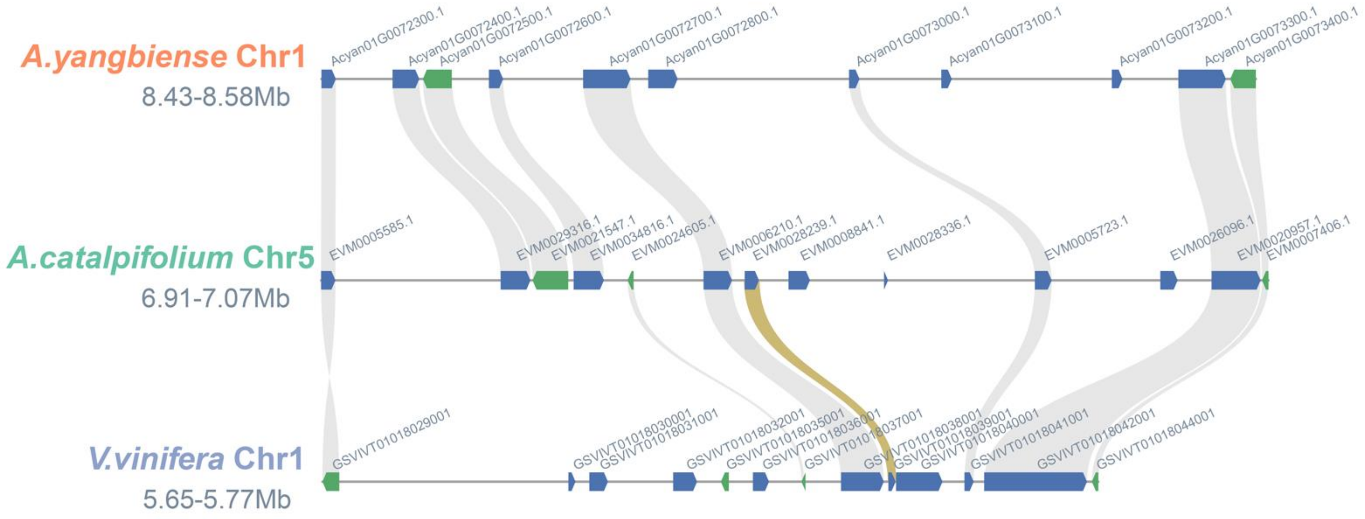

Figure S8. Microsynteny visualization of the matching regions along with the gene *EVM0028239.1* in *A. catalpifolium*, showing syntenic regions with *V. vinifera* and *A. yangbiense*.

The syntenic relationship between each two gene pairs were displayed in grey line, except for the target gene *EVM0028239.1*, which is highlighted in yellow.

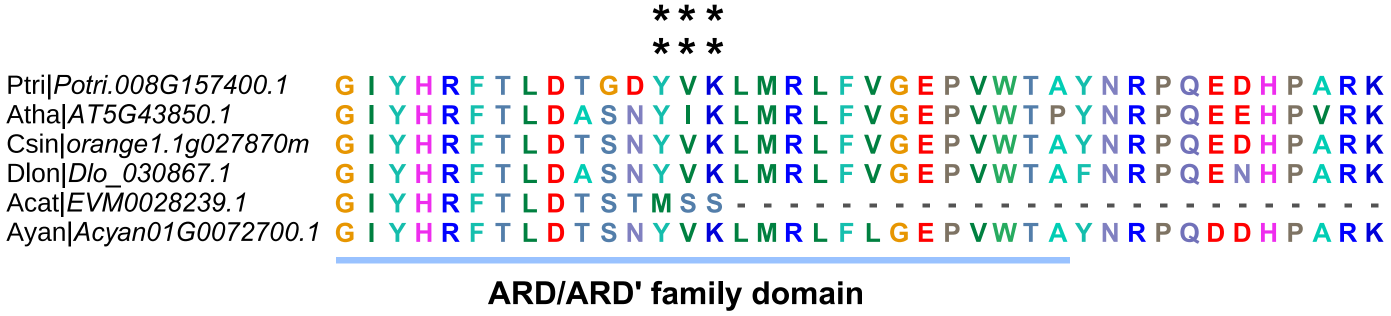

Figure S9. The protein sequence alignments of the C-terminal of the ARD/ARD' family domain in the PSGs of *A. catalpifolium* (*EVM0028239.1*) and orthologs in the other five species.

Six genes belonged to one orthogroup having the ARD/ARD' family domain. The C-terminal of the domain is marked with a straight line. Two asterisks above the alignment means that the site showed highly significant (*p-value* < 0.01) evidence of positive selection in *A. catalpifolium*.

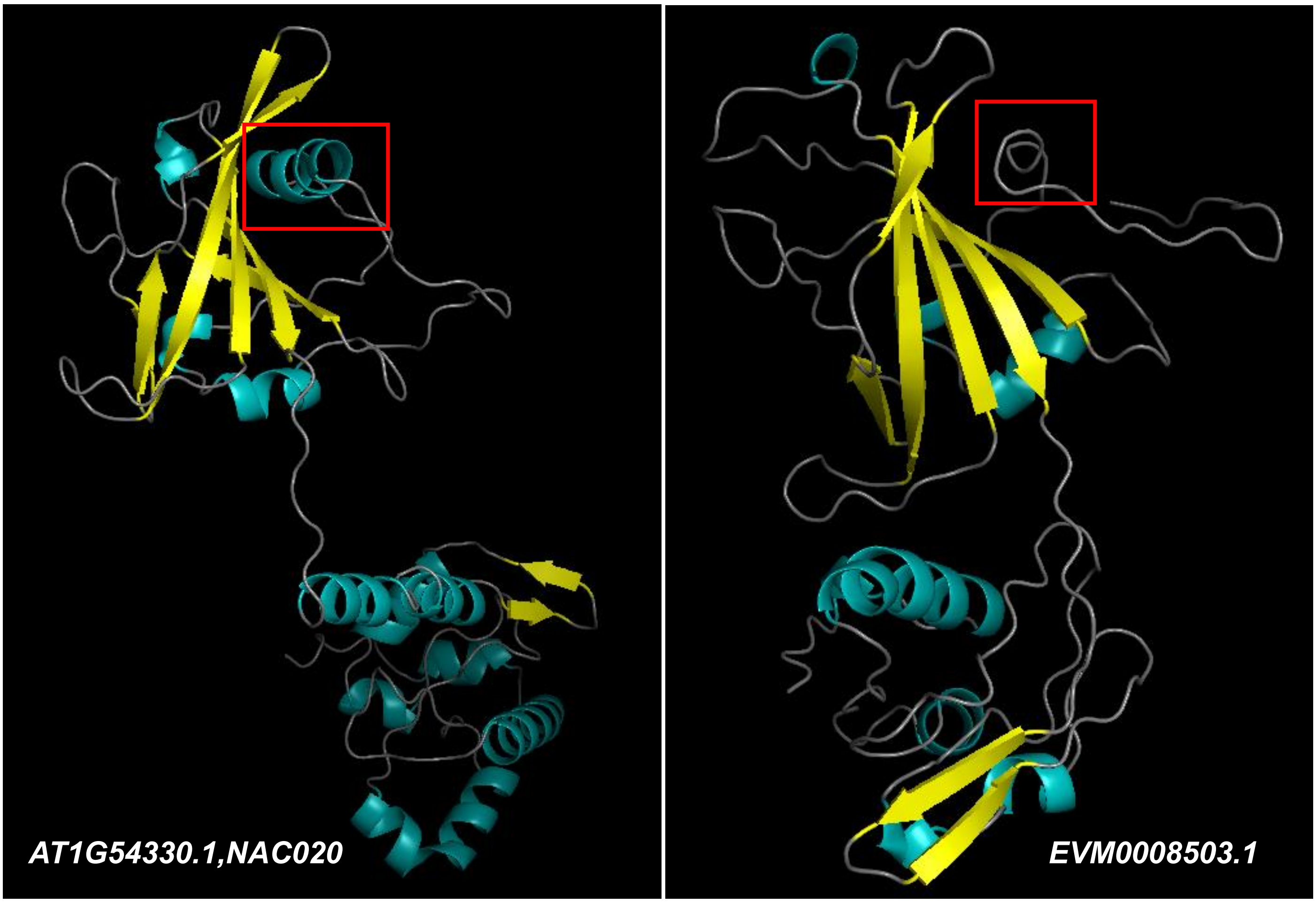

Figure S10. The predicted protein 3D structure of *AT1G54330.1* and its orthologous gene *EVM0008503.1* displayed in PyMOL. The protein domain containing positively selected sites was highlighted with red rectangle.

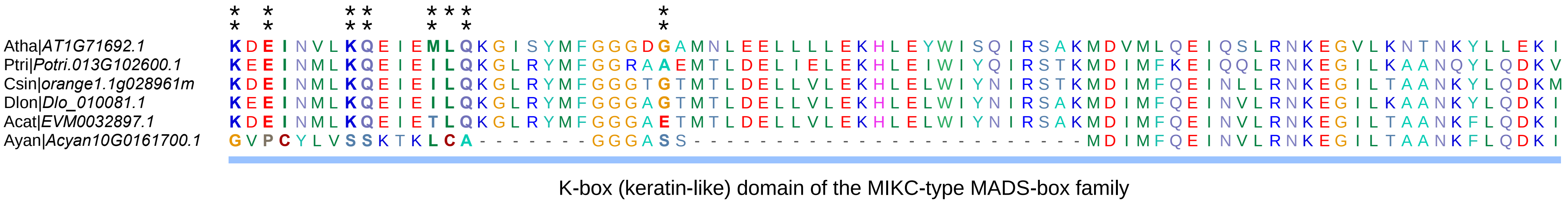

Figure S11. The protein sequence alignments of the conserved domains in the PSGs of *A. yangbiense* (*Acyan10G0161700.1*) and orthologs in five additional species.

The six genes belonged to one orthogroup annotated as the MIKC-type MADS-box gene family. One asterisk above the alignment means that the site was possibly (*p-value* < 0.05) under positive selection in *A. yangbiense*. Two asterisks above the alignment means that the site showed highly significant (*p-value* < 0.01) positive selection in *A. yangbiense*.

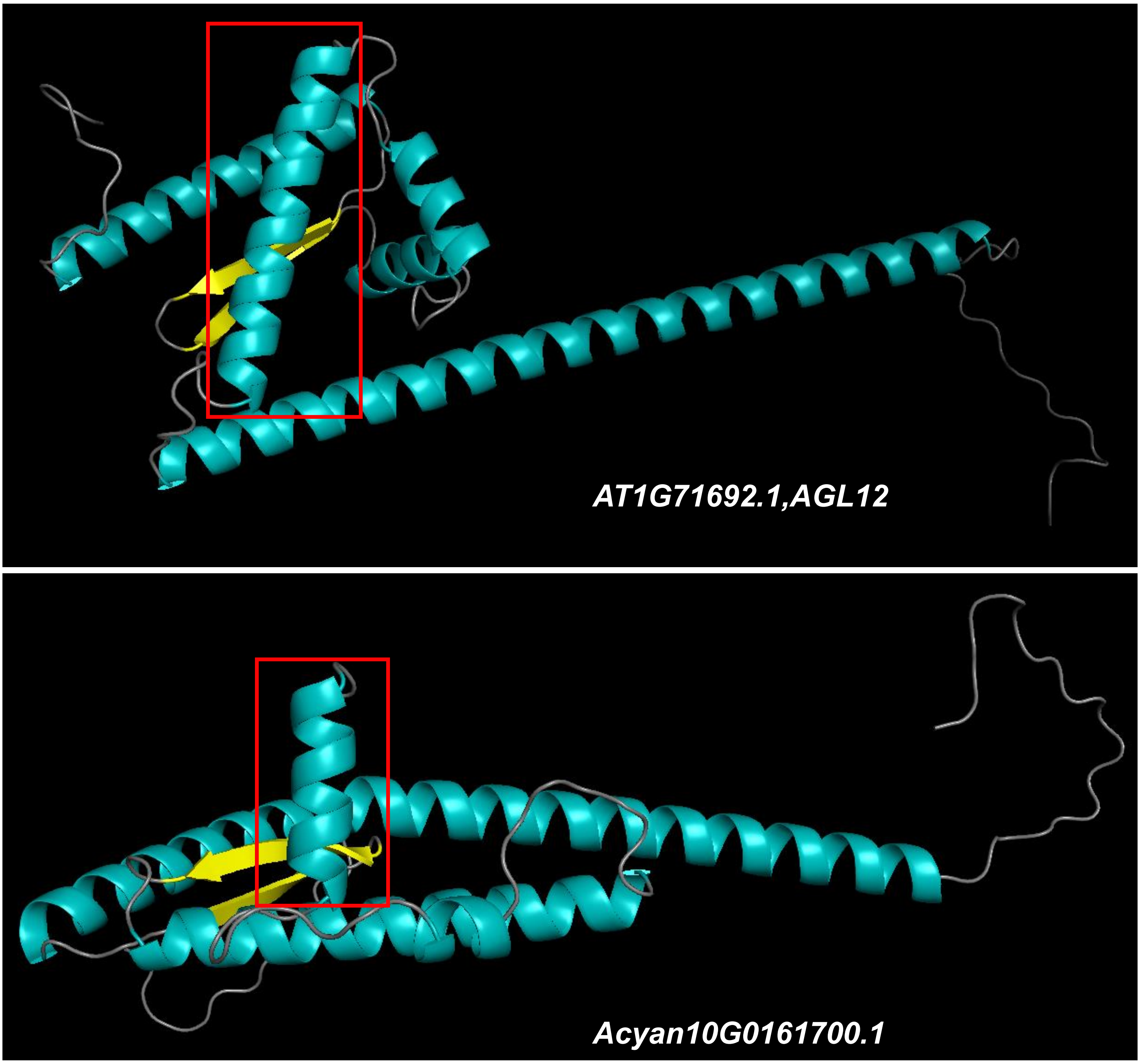

Figure S12. The predicted protein 3D structure of *AT1G71692.1* and its orthologous gene *Acyan10G0161700.1* displayed in PyMOL. The protein domain containing positively selected sites was highlighted with red rectangle.

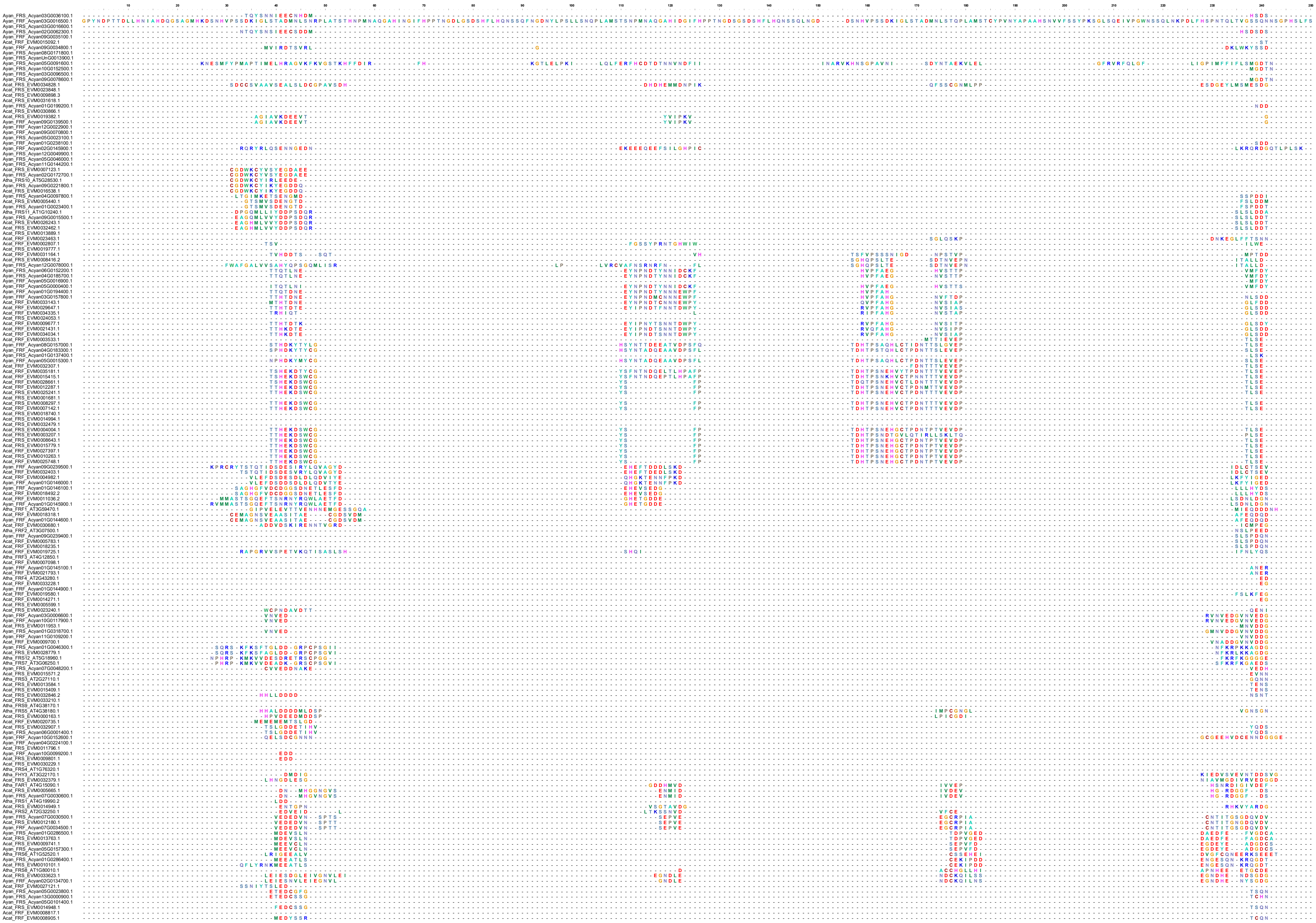

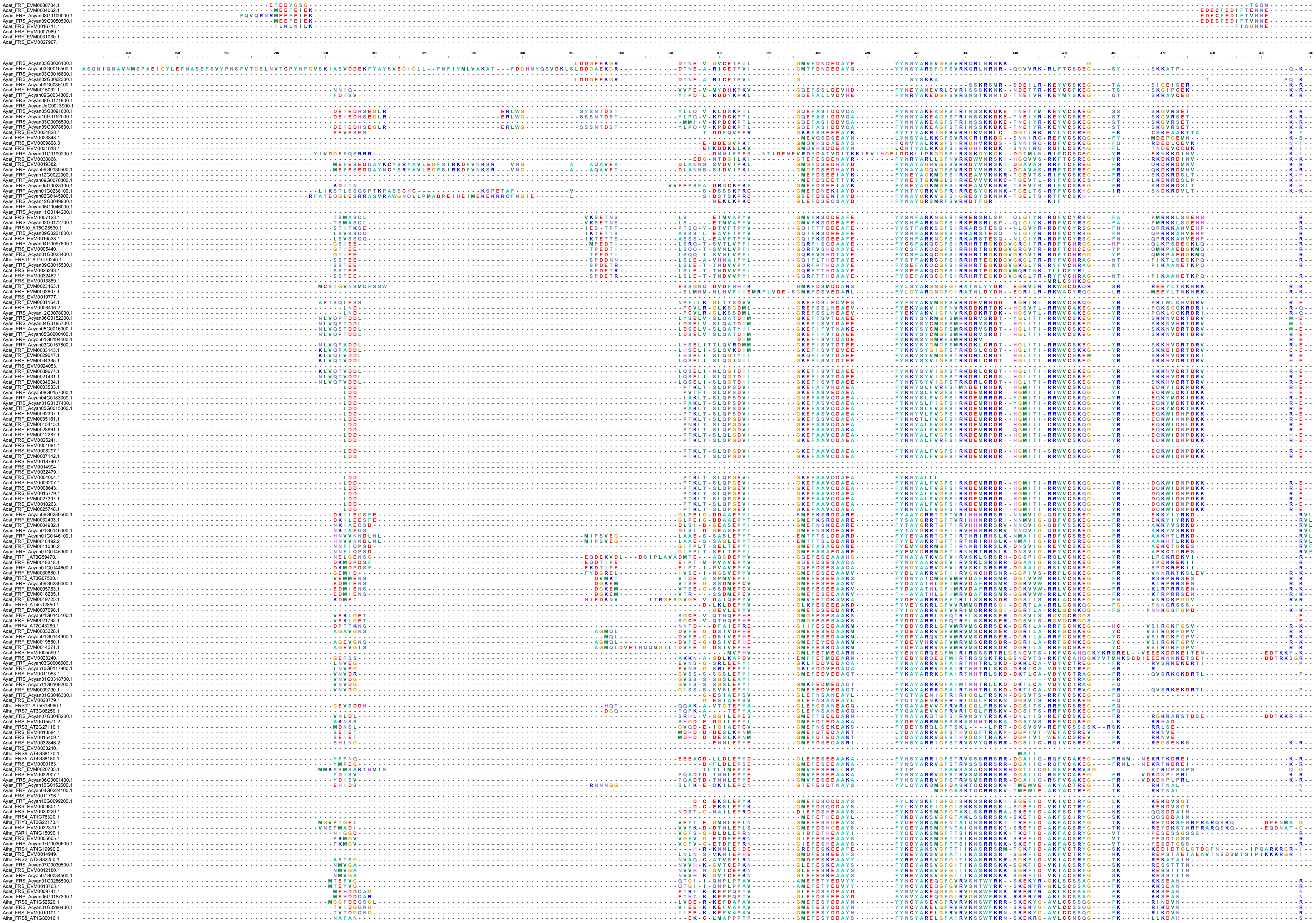

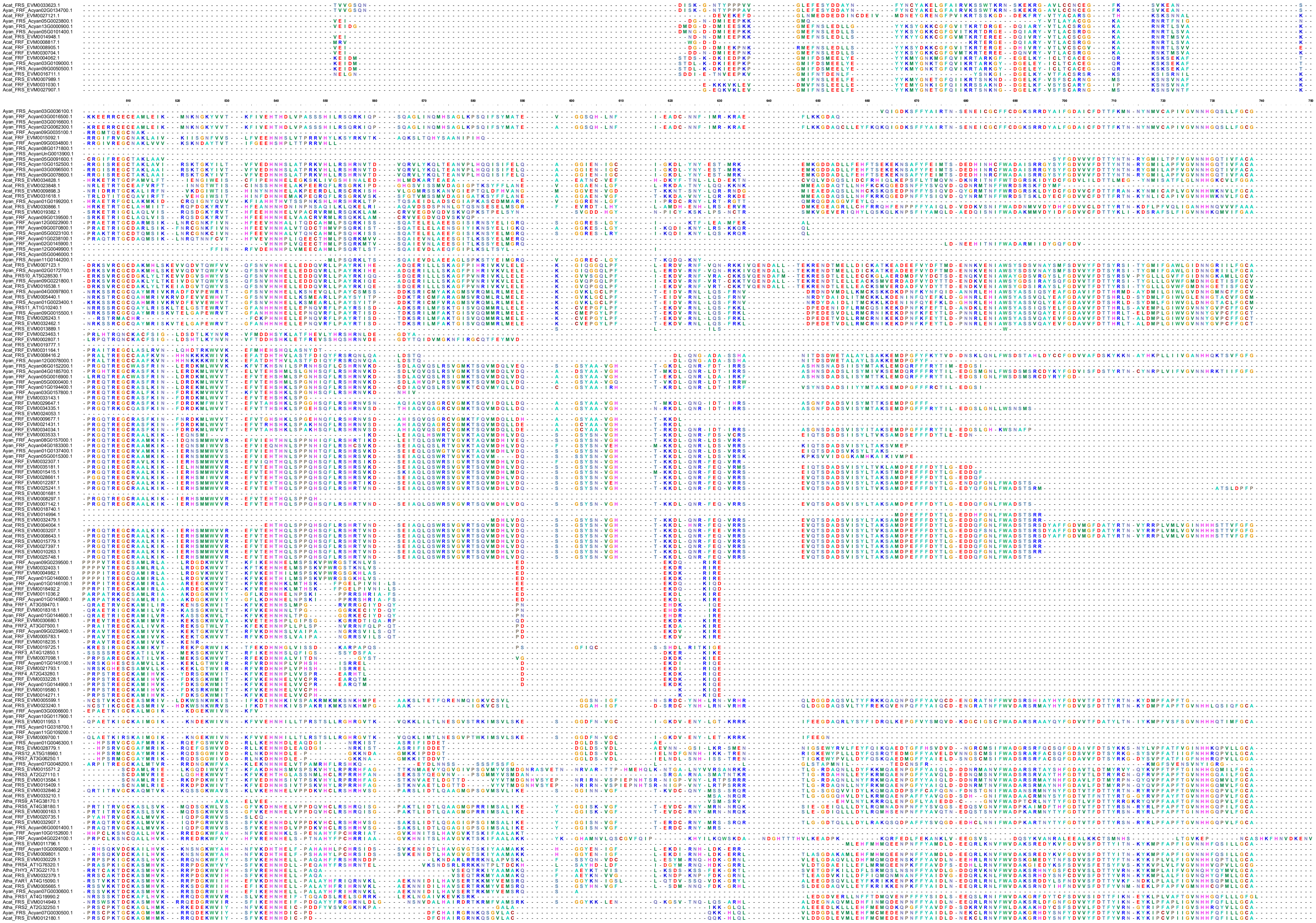

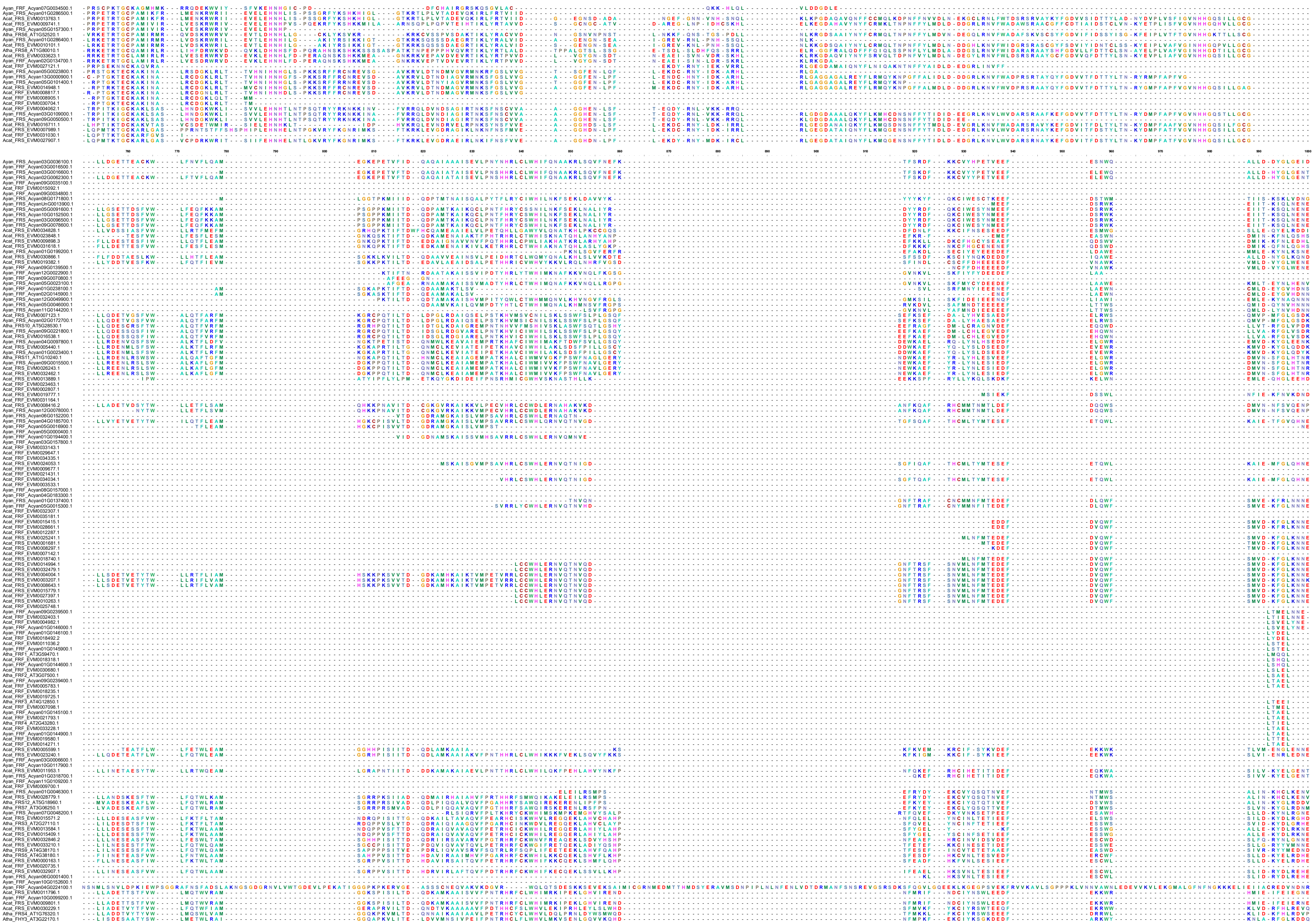

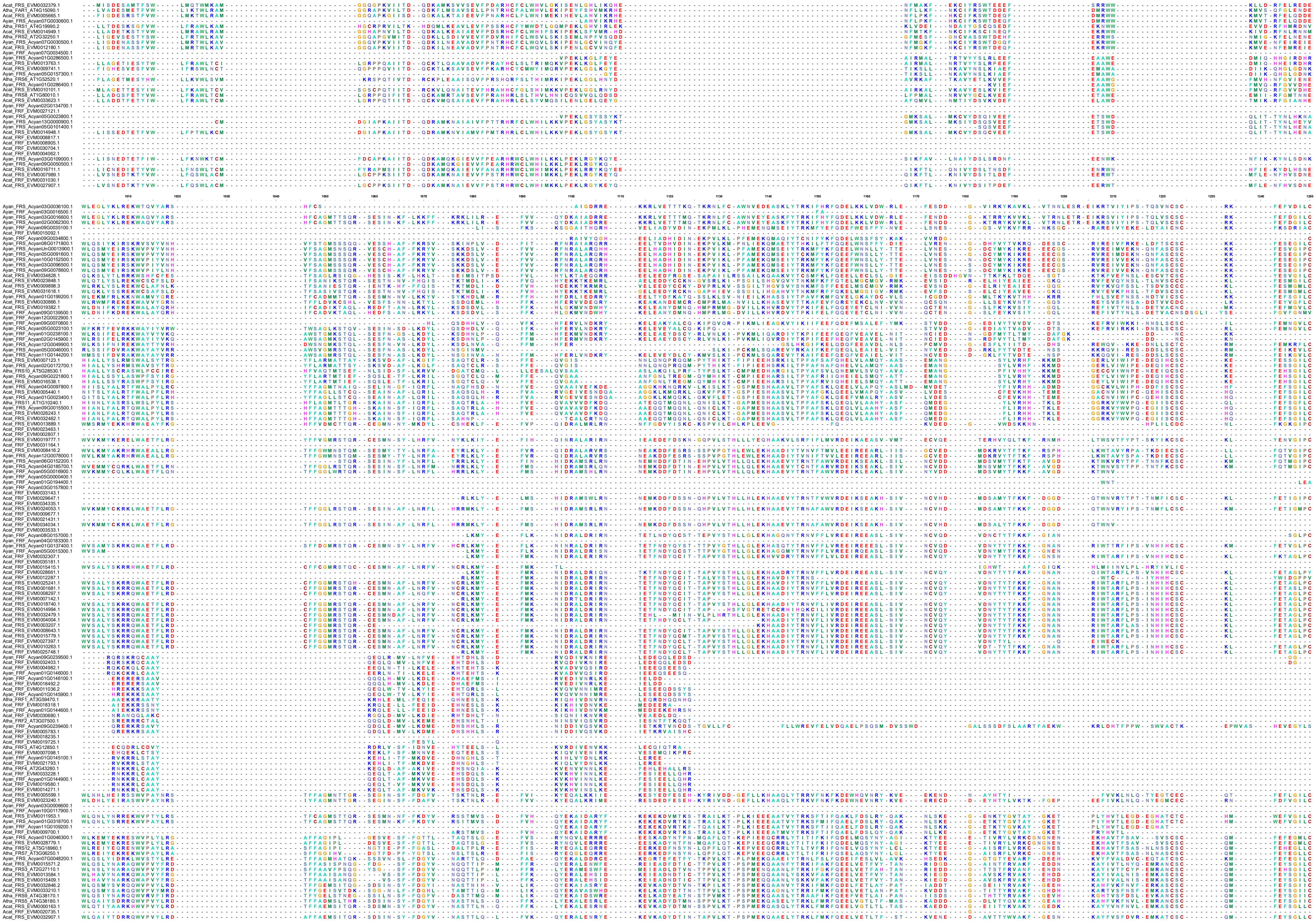

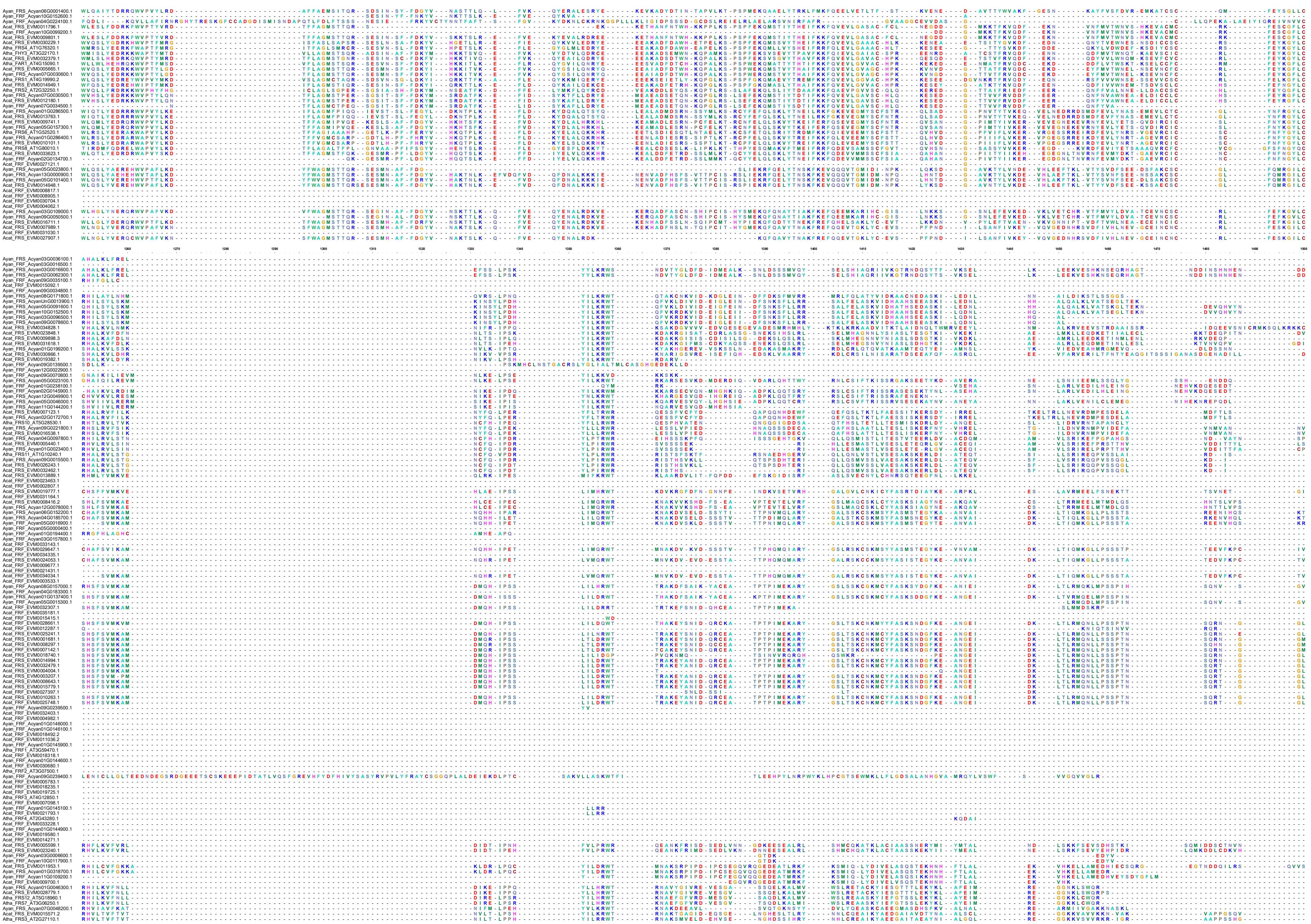

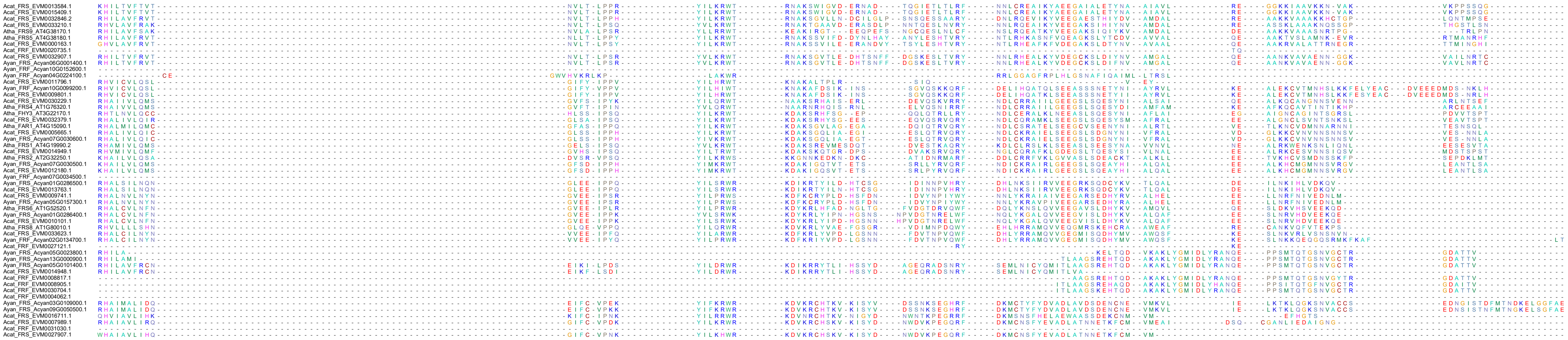

Figure S13. Visualization of multiple sequence alignments of the 179 FRS and FRF protein functional domains in three species (*Arabidopsis*, *A. catalpifolium* and *A. yangbiense*).

The multiple sequence alignments were printed in an order that was consistent with the phylogenetic tree. In the alignments, columns that contained protein functional domains were retained.

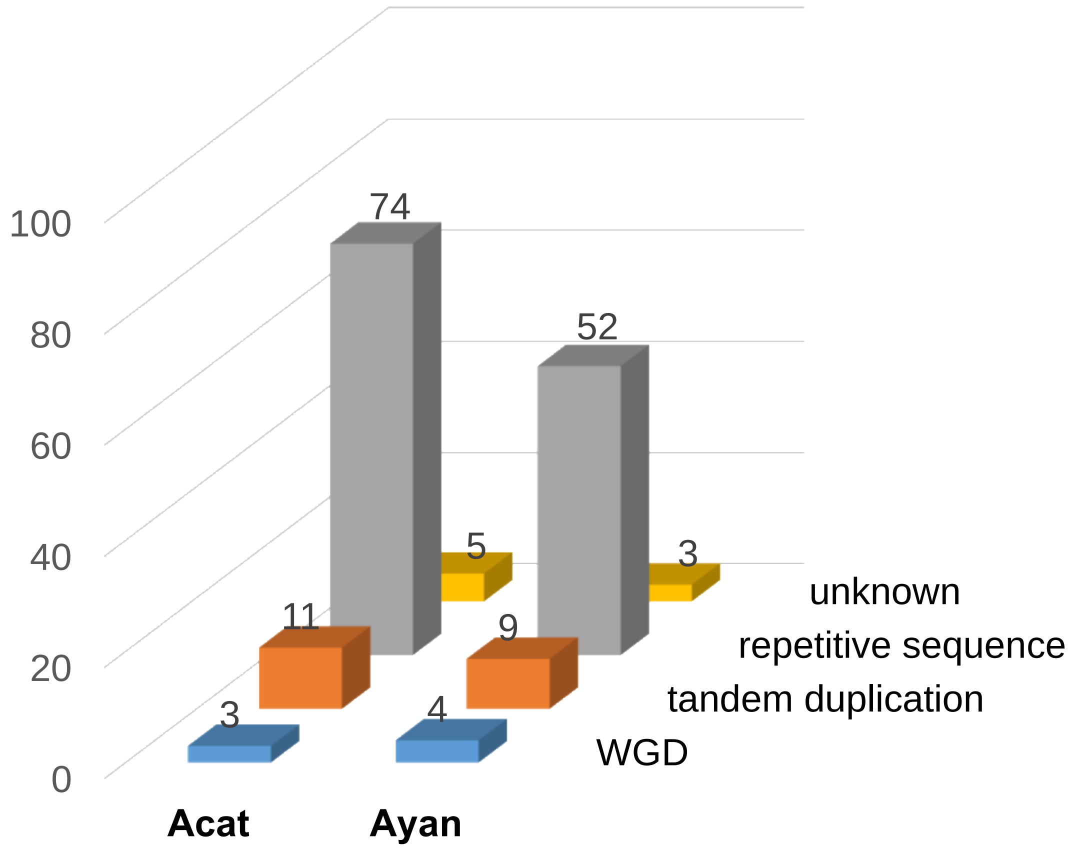

Figure S14. The number of genes in FRS-FRF gene family classified by four different types of gene duplication.

Acat represents *Acer catalpifolium*, and Ayan represents *Acer yangbiense*.

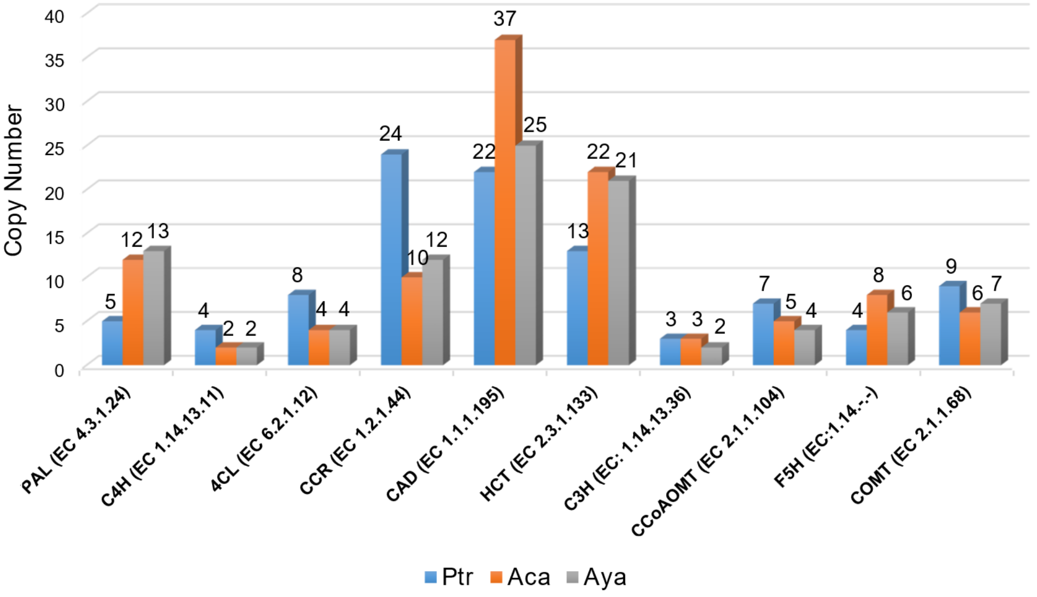

Figure S15. The detailed copy numbers of genes encoding 10 enzymes of the lignin biosynthesis pathway in three species (*Arabidopsis*, *A. catalpifolium* and *A. yangbiense*).

Ptr represents *Populus trichocarpa*, Aca represents *Acer catalpifolium*, and Aya represents *Acer yangbiense*.

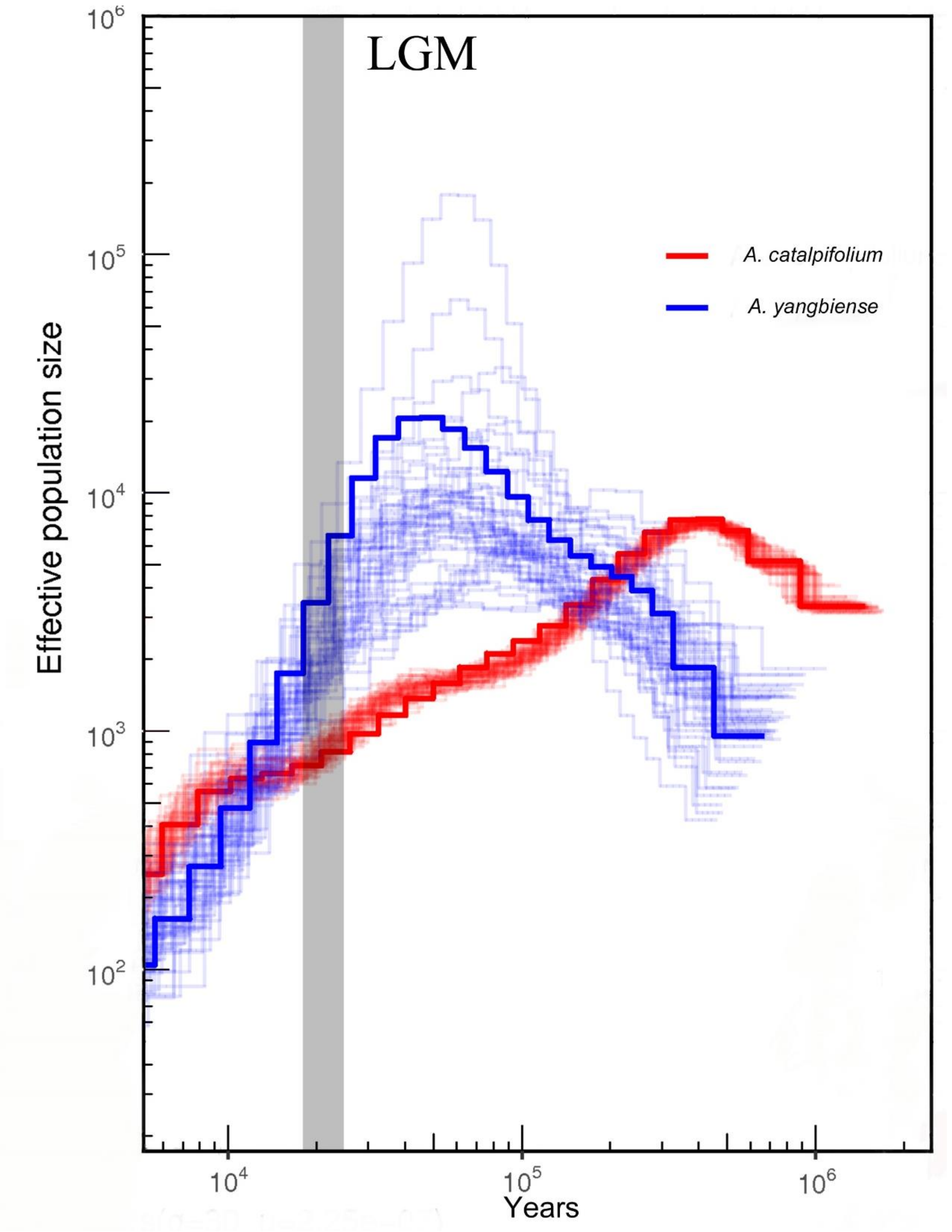

Figure S16. The inferred demographic history of *A. catalpifolium* and *A. yangbiense* using a Pairwise Sequentially Markovian Coalescence (PSMC) model.

Fifty bootstraps were performed and the results of the two *Acer* tree were colored differently. The grey area represents the last glacial maximum (LGM).

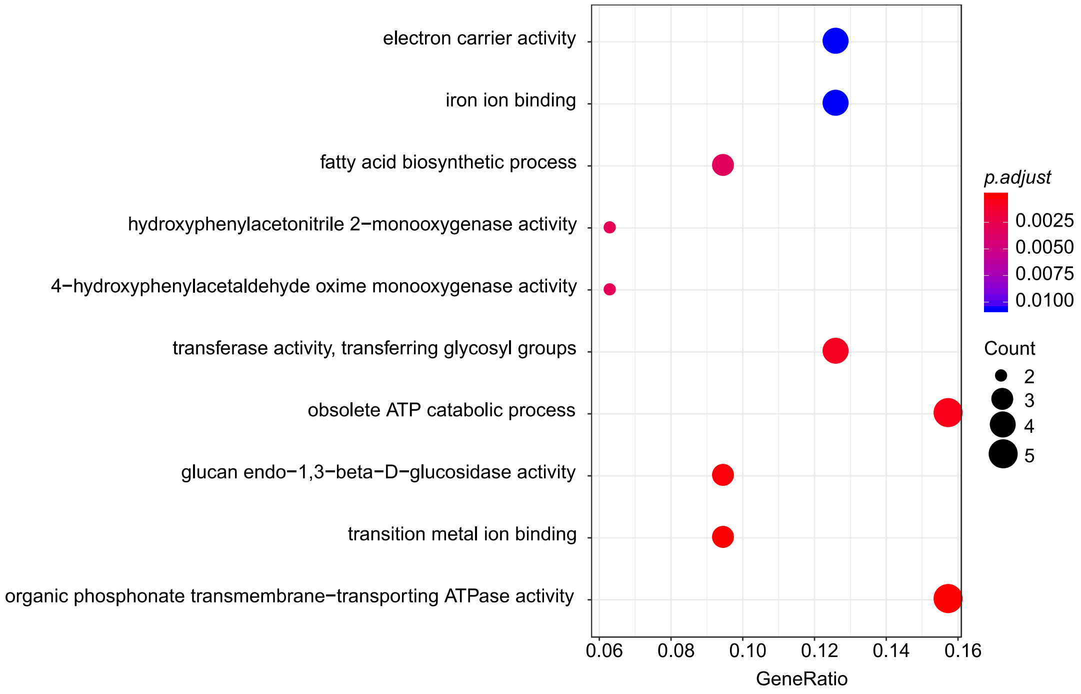

Figure S17. Visualization of results from GO enrichment analysis of 41 significantly contracted gene families in *A. catalpifolium*.

Top ten GO terms were selected for display after using Benjamini-Hochberg multiple test correction for *p*-value adjustment (adjusted *p-value* < 0.05).

Table S1. Statistical results of PacBio Sequel sequencing.

Platform	Type	Read Bases (bp)	Reads Num	Mean Reads Length(bp)
Sequel	Subreads	34,390,809,092	4,855,552	7083

Table S2. Statistical results of Illumina HiSeq sequencing.

Library	Data (Gb)	Depth (×)	Q20 (%)	Q30 (%)
350 bp	34.40	52.89	97.42	92.82
Total	34.40	52.89	-	-

Table S3. BUSCO evaluation of *A. catalpifolium* genome.

Complete BUSCOs	Complete and single-copy BUSCOs	Complete and duplicated BUSCOs	Fragmented BUSCOs	Missing BUSCOs
1344	1271	73	17	79

Table S4. Statistical results of predicted gene numbers using different methods and softwares.

Method	Software	Species	Gene number
Ab initio	Genscan	-	35,746
	Augustus	-	53,503
	GlimmerHMM	-	44,538
	GeneID	-	55,906
	SNAP	-	61,539
Homology-based	GeMoMa	*Arabidopsis_thaliana*	23,658
		Glycine_max	29,525
		Populus_trichocarpa	28,571
		Vitis_vinifera	23,719
		Prunus_persica	26,956
		Solanum_lycopersicum	27,774
RNA-seq	PASA	-	27,735
	GeneMarkS-T	-	39,906
	TransDecoder	-	66,677
Integration	EVM	-	35,132

Table S5. Number of annotated genes in *A. catalpifolium* genome using different functional annotation resources.

Annotation database	Annotated number	Percentage (%)
GO	16,985	48.08%
KEGG	10,348	29.29%
KOG	17,130	48.49%
Pfam	26,243	74.29%
Swissprot	22,406	63.43%
TrEMBL	32,128	90.95%
nr	32,115	90.91%
nt	30,178	85.43%
All	32,867	93.04%

Table S6. Statistical analyses of all repeat sequences in *A. catalpifolium* and *A. yangbiense* genomes. Information of *A. yangbiense* was from published data online (Yang et al., 2019).

Type	Number	Length（bp）	Percentage (%)	Percentage of *A. yangbiense* (%)
ClassI	604,776	335,872,896	51.32	-
ClassI/DIRS	29,488	21,545,631	3.29	-
ClassI/LINE	51,133	19,732,274	3.01	2.76
ClassI/LTR/Others	2776	1,053,418	0.16	-
ClassI/LTR/Copia	191,852	160,656,523	24.55	26.98
ClassI/LTR/Gypsy	111,269	91,812,477	14.03	9.94
ClassI/PLE|LARD	214,115	68,645,227	10.49	-
ClassI/SINE	3125	444,808	0.07	0.15
ClassI/TRIM	837	521,341	0.08	-
ClassI/Unknown	181	23,469	0	-
ClassII	123,940	37,375,835	5.71	-
ClassII/Crypton	8	425	0	0.6
ClassII/Helitron	28,369	7,530,621	1.15	0.38
ClassII/MITE	3268	657,652	0.1	-
ClassII/TIR	86,238	28,441,515	4.35	-
ClassII/Unknown	6057	1,186,483	0.18	-
PotentialHostGene	17,692	4,183,746	0.64	-
SSR	684	89,561	0.01	-
Unknown	83,296	22,570,060	3.45	17.32
Total	747,092	386,195,766	59	68

Table S7. GO enrichment of genes in rapidly expanding orthogroups of *A. catalpifolium* using all GO-annotated genes in *A. catalpifolium* genome as background.

**GO ID**	**GO terms**	**NO. of input genes overlap with specific GO**	**NO. of genes in specific GO**	***p value***	***adjusted p value***
GO:0042626	ATPase activity, coupled to transmembrane movement of substances	41	64	1.40E-37	4.34E-35
GO:0006200	obsolete ATP catabolic process	58	220	3.54E-26	5.51E-24
GO:0004674	protein serine/threonine kinase activity	86	488	8.19E-25	6.53E-23
GO:0070588	calcium ion transmembrane transport	21	24	8.40E-25	6.53E-23
GO:0005388	calcium-transporting ATPase activity	21	27	1.06E-22	6.61E-21
GO:0015079	potassium ion transmembrane transporter activity	20	25	4.05E-22	2.10E-20
GO:0071805	potassium ion transmembrane transport	20	30	1.80E-19	7.99E-18
GO:0015074	DNA integration	30	83	2.15E-18	8.35E-17
GO:0009922	fatty acid elongase activity	15	17	3.98E-18	1.38E-16
GO:0008422	beta-glucosidase activity	20	34	6.87E-18	2.14E-16
GO:0055085	transmembrane transport	55	365	3.00E-13	8.48E-12
GO:0008559	xenobiotic-transporting ATPase activity	15	28	6.49E-13	1.68E-11

GO ID	GO terms	NO. of input genes overlap with specific GO	NO. of genes in specific GO	*p value*	*adjusted p value*
GO:0010345	suberin biosynthetic process	11	16	1.80E-11	4.31E-10
GO:0004715	non-membrane spanning protein tyrosine kinase activity	22	91	5.96E-10	1.32E-08
GO:0008889	glycerophosphodiester phosphodiesterase activity	9	13	1.24E-09	2.56E-08
GO:0030246	carbohydrate binding	22	100	4.03E-09	7.83E-08
GO:0006071	glycerol metabolic process	9	15	7.90E-09	1.44E-07
GO:0016705	oxidoreductase activity, acting on paired donors, with incorporation or reduction of molecular oxygen	26	144	1.36E-08	2.35E-07
GO:0005516	calmodulin binding	19	81	1.51E-08	2.47E-07
GO:0004497	monooxygenase activity	26	147	2.11E-08	3.29E-07
GO:0016310	phosphorylation	48	426	1.68E-07	2.49E-06
GO:0032550	purine ribonucleoside binding	9	20	2.11E-07	2.85E-06
GO:0032559	adenyl ribonucleotide binding	9	20	2.11E-07	2.85E-06
GO:0045486	naringenin 3-dioxygenase activity	7	11	2.28E-07	2.95E-06
GO:0006970	response to osmotic stress	11	32	2.52E-07	3.13E-06

**GO ID**	**GO terms**	**NO. of input genes overlap with specific GO**	**NO. of genes in specific GO**	***p value***	***adjusted p value***
GO:0009416	response to light stimulus	16	70	3.02E-07	3.48E-06
GO:0016706	oxidoreductase activity, acting on paired donors, with incorporation or reduction of molecular oxygen, 2-oxoglutarate as one donor, and incorporation of one atom each of oxygen into both donors	16	70	3.02E-07	3.48E-06
GO:0004672	protein kinase activity	40	338	5.11E-07	5.68E-06
GO:0043168	anion binding	9	22	5.69E-07	6.10E-06
GO:0044710	single-organism metabolic process	15	67	9.40E-07	9.74E-06
GO:0005488	binding	48	453	1.03E-06	1.03E-05
GO:0004185	serine-type carboxypeptidase activity	12	44	1.22E-06	1.19E-05
GO:0009607	response to biotic stimulus	8	19	1.90E-06	1.77E-05
GO:0000413	protein peptidyl-prolyl isomerization	17	89	1.93E-06	1.77E-05
GO:0003755	peptidyl-prolyl cis-trans isomerase activity	17	90	2.27E-06	2.02E-05
GO:0006633	fatty acid biosynthetic process	17	94	4.23E-06	3.66E-05
GO:0045431	flavonol synthase activity	10	37	1.04E-05	8.73E-05

**GO ID**	**GO terms**	**NO. of input genes overlap with specific GO**	**NO. of genes in specific GO**	***p value***	***adjusted p value***
GO:0016758	transferase activity, transferring hexosyl groups	16	95	2.06E-05	1.68E-04
GO:0006606	protein import into nucleus	12	57	2.23E-05	1.74E-04
GO:0008565	protein transporter activity	12	57	2.23E-05	1.74E-04
GO:0016702	oxidoreductase activity, acting on single donors with incorporation of molecular oxygen, incorporation of two atoms of oxygen	13	67	2.59E-05	1.96E-04
GO:0000038	very long-chain fatty acid metabolic process	6	15	5.55E-05	4.11E-04
GO:0004180	carboxypeptidase activity	6	16	8.50E-05	6.15E-04
GO:0016746	transferase activity, transferring acyl groups	11	61	2.13E-04	1.50E-03
GO:0042335	cuticle development	6	20	3.46E-04	2.39E-03
GO:0005543	phospholipid binding	9	46	4.22E-04	2.85E-03
GO:0051555	flavonol biosynthetic process	10	58	5.94E-04	3.93E-03
GO:0031982	vesicle	4	10	1.07E-03	6.78E-03
GO:0043295	glutathione binding	4	10	1.07E-03	6.78E-03
GO:0009409	response to cold	17	148	1.33E-03	8.27E-03

GO ID	GO terms	NO. of input genes overlap with specific GO	NO. of genes in specific GO	*p value*	*adjusted p value*
GO:0009704	de-etiolation	4	11	1.61E-03	9.82E-03
GO:0005975	carbohydrate metabolic process	26	278	1.92E-03	1.15E-02
GO:0033179	proton-transporting V-type ATPase, V0 domain	4	12	2.32E-03	1.36E-02
GO:0016772	transferase activity, transferring phosphorus-containing groups	16	144	2.56E-03	1.47E-02
GO:0048038	quinone binding	7	38	2.63E-03	1.49E-02
GO:0048527	lateral root development	5	22	4.19E-03	2.33E-02
GO:0016491	oxidoreductase activity	37	474	5.96E-03	3.25E-02
GO:0032440	2-alkenal reductase [NAD(P)] activity	25	289	6.38E-03	3.42E-02
GO:0004714	transmembrane receptor protein tyrosine kinase activity	4	16	7.26E-03	3.83E-02
GO:0016709	oxidoreductase activity, acting on paired donors, with incorporation or reduction of molecular oxygen, NAD(P)H as one donor, and incorporation of one atom of oxygen	5	26	8.86E-03	4.59E-02

Table S8. GO enrichment of 159 *Acer* genus-specific rapidly evolving genes using all GO-annotated genes in *A. catalpifolium* genome as background.

GO ID	GO terms	NO. of input genes overlap with specific GO	NO. of genes in specific GO	*p value*	*adjusted p value*
GO:0000413	protein peptidyl-prolyl isomerization	17	89	3.29E-28	6.27E-27
GO:0003755	peptidyl-prolyl cis-trans isomerase activity	17	90	4.05E-28	6.27E-27
GO:0006457	protein folding	17	232	8.73E-21	9.02E-20
GO:0048046	apoplast	12	217	2.77E-13	2.14E-12
GO:0046686	response to cadmium ion	12	288	7.85E-12	4.87E-11
GO:0050662	coenzyme binding	7	85	2.38E-09	1.23E-08
GO:0044237	cellular metabolic process	7	140	7.77E-08	3.44E-07
GO:0006260	DNA replication	5	55	3.27E-07	1.27E-06
GO:0043414	macromolecule methylation	3	10	2.06E-06	7.09E-06
GO:0003824	catalytic activity	7	285	9.15E-06	2.68E-05
GO:0006783	heme biosynthetic process	3	16	9.50E-06	2.68E-05
GO:0010468	regulation of gene expression	3	17	1.15E-05	2.97E-05

**GO ID**	**GO terms**	**NO. of input genes overlap with specific GO**	**NO. of genes in specific GO**	***p value***	***adjusted p value***
GO:0050896	response to stimulus	5	122	1.71E-05	4.08E-05
GO:0007049	cell cycle	3	22	2.58E-05	5.72E-05
GO:0016740	transferase activity	6	289	1.07E-04	2.22E-04
GO:0050897	cobalt ion binding	3	41	1.73E-04	3.35E-04
GO:0060255	regulation of macromolecule metabolic process	2	15	7.05E-04	1.29E-03
GO:0050794	regulation of cellular process	3	68	7.72E-04	1.33E-03
GO:0043170	macromolecule metabolic process	2	18	1.02E-03	1.67E-03
GO:0044267	cellular protein metabolic process	2	38	4.54E-03	7.04E-03
GO:0016853	isomerase activity	2	48	7.17E-03	1.06E-02
GO:0005840	ribosome	3	165	9.48E-03	1.30E-02
GO:0044464	cell part	2	56	9.65E-03	1.30E-02
GO:0044238	primary metabolic process	2	102	2.99E-02	3.86E-02

Table S9. Detailed information about the 969 PSGs-related orthogroups.

**OGs ID**	**having site under significant positive selection**	**having site under highly significant positive selection**	***p value***
13Plants01460	✓	✓	0.00
13Plants01991	✓	✓	0.00
13Plants02565	✓	✓	0.00
13Plants02622	✓	✓	0.00
13Plants02708	✓	✓	0.00
13Plants02788	✓	✓	0.00
13Plants03005	✓	✓	0.00
13Plants03123	✓	✓	0.00
13Plants03172	✓	✓	0.00
13Plants03185	✓	✓	0.00
13Plants03375	✓	✓	0.00
13Plants03558	✓	✓	0.00
13Plants03690	✓	✓	0.00
13Plants03706	✓	✓	0.00
13Plants03753	✓	✓	0.00
13Plants03852	✓	✓	0.00
13Plants03879	✓	✓	0.00
13Plants03912	✓	✓	0.00
13Plants04052	✓	✓	0.00
13Plants04053	✓	✓	0.00
13Plants04223	✓	✓	0.00
13Plants04232	✓	✓	0.00
13Plants04262	✓	✓	0.00
13Plants04288	✓	✓	0.00
13Plants04326	✓	✓	0.00
13Plants04364	✓	✓	0.00
13Plants04435	✓	✓	0.00
13Plants04441	✓	✓	0.00
13Plants04572	✓	✓	0.00
13Plants04856	✓	✓	0.00
13Plants04893	✓	✓	0.00

OGs ID	having site under significant positive selection	having site under highly significant positive selection	P value
13Plants04910	✓	✓	0.00
13Plants04915	✓	✓	0.00
13Plants04920	✓	✓	0.00
13Plants04927	✓	✓	0.00
13Plants04941	✓	✓	0.00
13Plants05132	✓	✓	0.00
13Plants05148	✓	✓	0.00
13Plants05187	✓	✓	0.00
13Plants05209	✓	✓	0.00
13Plants05231	✓	✓	0.00
13Plants05251	✓	✓	0.00
13Plants05348	✓	✓	0.00
13Plants05407	✓	✓	0.00
13Plants05442	✓	✓	0.00
13Plants05681	✓	✓	0.00
13Plants05741	✓	✓	0.00
13Plants05767	✓	✓	0.00
13Plants05808	✓	✓	0.00
13Plants05821	✓	✓	0.00
13Plants05849	✓	✓	0.00
13Plants05871	✓	✓	0.00
13Plants05937	✓	✓	0.00
13Plants05977	✓	✓	0.00
13Plants05978	✓	✓	0.00
13Plants06002	✓	✓	0.00
13Plants06064	✓	✓	0.00
13Plants06136	✓	✓	0.00
13Plants06146	✓	✓	0.00
13Plants06234	✓	✓	0.00
13Plants06252	✓	✓	0.00
13Plants06316	✓	✓	0.00
13Plants06358	✓	✓	0.00
13Plants06370	✓	✓	0.00

OGs ID	having site under significant positive selection	having site under highly significant positive selection	P value
13Plants06413	✓	✓	0.00
13Plants06428	✓	✓	0.00
13Plants06433	✓	✓	0.00
13Plants06696	✓	✓	0.00
13Plants06742	✓	✓	0.00
13Plants06749	✓	✓	0.00
13Plants06751	✓	✓	0.00
13Plants06755	✓	✓	0.00
13Plants06759	✓	✓	0.00
13Plants06804	✓	✓	0.00
13Plants06871	✓	✓	0.00
13Plants06881	✓	✓	0.00
13Plants06904	✓	✓	0.00
13Plants06930	✓	✓	0.00
13Plants06935	✓	✓	0.00
13Plants06951	✓	✓	0.00
13Plants06954	✓	✓	0.00
13Plants06990	✓	✓	0.00
13Plants07011	✓	✓	0.00
13Plants07082	✓	✓	0.00
13Plants07195	✓	✓	0.00
13Plants07227	✓	✓	0.00
13Plants07261	✓	✓	0.00
13Plants07277	✓	✓	0.00
13Plants07286	✓	✓	0.00
13Plants07330	✓	✓	0.00
13Plants07338	✓	✓	0.00
13Plants07419	✓	✓	0.00
13Plants07433	✓	✓	0.00
13Plants07463	✓	✓	0.00
13Plants07499	✓	✓	0.00
13Plants07508	✓	✓	0.00
13Plants07512	✓	✓	0.00

OGs ID	having site under significant positive selection	having site under highly significant positive selection	P value
13Plants07530	✓	✓	0.00
13Plants07569	✓	✓	0.00
13Plants07588	✓	✓	0.00
13Plants07616	✓	✓	0.00
13Plants07617	✓	✓	0.00
13Plants07640	✓	✓	0.00
13Plants07661	✓	✓	0.00
13Plants07679	✓	✓	0.00
13Plants07693	✓	✓	0.00
13Plants07717	✓	✓	0.00
13Plants07866	✓	✓	0.00
13Plants07918	✓	✓	0.00
13Plants07946	✓	✓	0.00
13Plants07959	✓	✓	0.00
13Plants07968	✓	✓	0.00
13Plants07989	✓	✓	0.00
13Plants08199	✓	✓	0.00
13Plants08212	✓	✓	0.00
13Plants08256	✓	✓	0.00
13Plants08300	✓	✓	0.00
13Plants08341	✓	✓	0.00
13Plants08368	✓	✓	0.00
13Plants08386	✓	✓	0.00
13Plants08393	✓	✓	0.00
13Plants08436	✓	✓	0.00
13Plants08449	✓	✓	0.00
13Plants08473	✓	✓	0.00
13Plants08504	✓	✓	0.00
13Plants08513	✓	✓	0.00
13Plants08524	✓	✓	0.00
13Plants08543	✓	✓	0.00
13Plants08548	✓	✓	0.00
13Plants08577	✓	✓	0.00

OGs ID	having site under significant positive selection	having site under highly significant positive selection	P value
13Plants08605	✓	✓	0.00
13Plants08659	✓	✓	0.00
13Plants08661	✓	✓	0.00
13Plants08666	✓	✓	0.00
13Plants08691	✓	✓	0.00
13Plants08775	✓	✓	0.00
13Plants08795	✓	✓	0.00
13Plants08832	✓	✓	0.00
13Plants08864	✓	✓	0.00
13Plants08905	✓	✓	0.00
13Plants08954	✓	✓	0.00
13Plants09038	✓	✓	0.00
13Plants09299	✓	✓	0.00
13Plants09404	✓	✓	0.00
13Plants09414	✓	✓	0.00
13Plants09652	✓	✓	0.00
13Plants09707	✓	✓	0.00
13Plants09721	✓	✓	0.00
13Plants09837	✓	✓	0.00
13Plants09843	✓	✓	0.00
13Plants09856	✓	✓	0.00
13Plants09872	✓	✓	0.00
13Plants09902	✓	✓	0.00
13Plants09935	✓	✓	0.00
13Plants09939	✓	✓	0.00
13Plants09962	✓	✓	0.00
13Plants09989	✓	✓	0.00
13Plants09995	✓	✓	0.00
13Plants10057	✓	✓	0.00
13Plants10066	✓	✓	0.00
13Plants10084	✓	✓	0.00
13Plants10087	✓	✓	0.00
13Plants10136	✓	✓	0.00

OGs ID	having site under significant positive selection	having site under highly significant positive selection	P value
13Plants10162	✓	✓	0.00
13Plants10178	✓	✓	0.00
13Plants10199	✓	✓	0.00
13Plants10233	✓	✓	0.00
13Plants10285	✓	✓	0.00
13Plants10329	✓	✓	0.00
13Plants10368	✓	✓	0.00
13Plants10375	✓	✓	0.00
13Plants10408	✓	✓	0.00
13Plants10412	✓	✓	0.00
13Plants10459	✓	✓	0.00
13Plants10542	✓	✓	0.00
13Plants10558	✓	✓	0.00
13Plants10562	✓	✓	0.00
13Plants10576	✓	✓	0.00
13Plants10593	✓	✓	0.00
13Plants10605	✓	✓	0.00
13Plants10614	✓	✓	0.00
13Plants10647	✓	✓	0.00
13Plants10670	✓	✓	0.00
13Plants10676	✓	✓	0.00
13Plants10677	✓	✓	0.00
13Plants10810	✓	✓	0.00
13Plants10813	✓	✓	0.00
13Plants10924	✓	✓	0.00
13Plants10933	✓	✓	0.00
13Plants11052	✓	✓	0.00
13Plants11211	✓	✓	0.00
13Plants11242	✓	✓	0.00
13Plants11243	✓	✓	0.00
13Plants11290	✓	✓	0.00
13Plants11337	✓	✓	0.00
13Plants11375	✓	✓	0.00

OGs ID	having site under significant positive selection	having site under highly significant positive selection	P value
13Plants11383	✓	✓	0.00
13Plants11408	✓	✓	0.00
13Plants11536	✓	✓	0.00
13Plants11566	✓	✓	0.00
13Plants11810	✓	✓	0.00
13Plants12501	✓	✓	0.00
13Plants12633	✓	✓	0.00
13Plants12867	✓	✓	0.00
13Plants13369	✓	✓	0.00
13Plants13423	✓	✓	0.00
13Plants14631	✓	✓	0.00
13Plants14653	✓	✓	0.00
13Plants14655	✓	✓	0.00
13Plants15404	✓	✓	0.00
13Plants15451	✓	✓	0.00
13Plants03677	-	✓	0.00
13Plants04013	-	✓	0.00
13Plants04923	-	✓	0.00
13Plants05293	-	✓	0.00
13Plants06242	-	✓	0.00
13Plants06854	-	✓	0.00
13Plants09880	-	✓	0.00
13Plants10322	-	✓	0.00
13Plants11202	-	✓	0.00
13Plants12043	-	✓	0.00
13Plants03863	✓	-	0.00
13Plants08310	✓	-	0.00
13Plants08356	✓	-	0.00
13Plants10371	✓	-	0.00
13Plants06098	✓	-	1.00E-09
13Plants09598	✓	-	1.00E-09
13Plants03689	✓	✓	1.00E-09
13Plants03837	✓	✓	1.00E-09

OGs ID	having site under significant positive selection	having site under highly significant positive selection	P value
13Plants04204	✓	✓	1.00E-09
13Plants04394	✓	✓	1.00E-09
13Plants04662	✓	✓	1.00E-09
13Plants05405	✓	✓	1.00E-09
13Plants05921	✓	✓	1.00E-09
13Plants07548	✓	✓	1.00E-09
13Plants08656	✓	✓	1.00E-09
13Plants09235	✓	✓	1.00E-09
13Plants10430	✓	✓	1.00E-09
13Plants10461	✓	✓	1.00E-09
13Plants10657	✓	✓	1.00E-09
13Plants12234	✓	✓	1.00E-09
13Plants07447	-	✓	1.00E-09
13Plants09271	-	✓	1.00E-09
13Plants02609	✓	✓	2.00E-09
13Plants05238	✓	✓	2.00E-09
13Plants06879	✓	✓	2.00E-09
13Plants10053	✓	✓	2.00E-09
13Plants10163	✓	✓	2.00E-09
13Plants10685	✓	✓	2.00E-09
13Plants10947	✓	✓	2.00E-09
13Plants06898	✓	✓	3.00E-09
13Plants08108	✓	✓	3.00E-09
13Plants09884	✓	✓	3.00E-09
13Plants10108	✓	✓	3.00E-09
13Plants10549	✓	✓	3.00E-09
13Plants05884	✓	✓	4.00E-09
13Plants10344	✓	✓	4.00E-09
13Plants08208	✓	✓	5.00E-09
13Plants09531	✓	✓	5.00E-09
13Plants09281	✓	-	6.00E-09
13Plants04546	✓	✓	6.00E-09
13Plants08632	✓	✓	6.00E-09

OGs ID	having site under significant positive selection	having site under highly significant positive selection	P value
13Plants14035	✓	✓	6.00E-09
13Plants00013	-	✓	6.00E-09
13Plants06244	✓	✓	7.00E-09
13Plants07900	✓	✓	7.00E-09
13Plants10313	-	✓	7.00E-09
13Plants06285	✓	✓	8.00E-09
13Plants06983	✓	✓	8.00E-09
13Plants04282	✓	✓	9.00E-09
13Plants09915	✓	✓	9.00E-09
13Plants10636	✓	✓	9.00E-09
13Plants03171	✓	✓	1.00E-08
13Plants05746	✓	✓	1.00E-08
13Plants10122	✓	✓	1.10E-08
13Plants11419	✓	✓	1.10E-08
13Plants06122	✓	✓	1.20E-08
13Plants07385	✓	✓	1.20E-08
13Plants08871	✓	✓	1.20E-08
13Plants12922	✓	✓	1.30E-08
13Plants10688	✓	✓	1.40E-08
13Plants07851	✓	✓	1.50E-08
13Plants08026	✓	✓	1.50E-08
13Plants09783	✓	✓	1.50E-08
13Plants12345	✓	✓	1.60E-08
13Plants06185	✓	✓	2.10E-08
13Plants11266	✓	✓	2.10E-08
13Plants03840	✓	✓	2.20E-08
13Plants08897	✓	✓	2.30E-08
13Plants07593	-	✓	2.60E-08
13Plants02878	✓	✓	2.90E-08
13Plants06944	✓	✓	3.10E-08
13Plants13354	✓	✓	3.10E-08
13Plants01117	✓	✓	3.20E-08
13Plants07522	✓	✓	3.20E-08

OGs ID	having site under significant positive selection	having site under highly significant positive selection	P value
13Plants06201	✓	✓	3.50E-08
13Plants06843	✓	✓	3.60E-08
13Plants05197	✓	-	3.70E-08
13Plants04665	✓	✓	3.80E-08
13Plants07650	✓	✓	3.80E-08
13Plants05673	✓	✓	4.20E-08
13Plants08964	✓	-	4.40E-08
13Plants10479	✓	✓	4.40E-08
13Plants06114	✓	✓	4.50E-08
13Plants07435	✓	✓	4.60E-08
13Plants08299	✓	✓	5.20E-08
13Plants11456	✓	-	5.40E-08
13Plants02125	✓	✓	6.00E-08
13Plants06331	✓	✓	6.10E-08
13Plants09164	✓	✓	6.50E-08
13Plants05696	✓	✓	8.00E-08
13Plants03114	✓	✓	8.30E-08
13Plants04303	✓	✓	8.60E-08
13Plants07324	✓	✓	9.30E-08
13Plants02740	✓	✓	9.80E-08
13Plants08480	✓	✓	9.90E-08
13Plants03435	✓	✓	1.02E-07
13Plants08192	✓	✓	1.09E-07
13Plants05091	✓	✓	1.14E-07
13Plants03853	✓	✓	1.16E-07
13Plants03331	✓	✓	1.20E-07
13Plants05173	✓	✓	1.20E-07
13Plants06901	✓	✓	1.26E-07
13Plants03955	✓	✓	1.27E-07
13Plants05427	✓	✓	1.30E-07
13Plants08633	✓	✓	1.34E-07
13Plants04627	✓	✓	1.40E-07
13Plants14024	✓	✓	1.43E-07

OGs ID	having site under significant positive selection	having site under highly significant positive selection	P value
13Plants06267	✓	✓	1.45E-07
13Plants05281	✓	✓	1.51E-07
13Plants13662	✓	✓	1.63E-07
13Plants05068	-	✓	1.78E-07
13Plants07335	-	✓	1.88E-07
13Plants06287	✓	✓	2.04E-07
13Plants07285	✓	✓	2.30E-07
13Plants07020	✓	✓	2.34E-07
13Plants13928	✓	✓	2.37E-07
13Plants04413	✓	-	2.65E-07
13Plants05137	✓	✓	2.81E-07
13Plants08471	✓	✓	2.89E-07
13Plants09417	✓	-	2.97E-07
13Plants04297	✓	✓	3.09E-07
13Plants09268	✓	✓	3.28E-07
13Plants10302	✓	✓	3.61E-07
13Plants09879	✓	✓	3.73E-07
13Plants02801	-	✓	3.98E-07
13Plants04218	✓	-	4.03E-07
13Plants04455	✓	✓	4.03E-07
13Plants05105	✓	✓	4.10E-07
13Plants10609	✓	✓	4.25E-07
13Plants04324	✓	✓	4.30E-07
13Plants02828	✓	✓	4.46E-07
13Plants05418	✓	✓	4.64E-07
13Plants10646	✓	✓	5.03E-07
13Plants10121	✓	-	5.19E-07
13Plants07637	✓	✓	5.33E-07
13Plants09999	-	✓	5.41E-07
13Plants08005	✓	✓	5.62E-07
13Plants10064	✓	✓	5.80E-07
13Plants07090	✓	✓	5.90E-07
13Plants03430	-	✓	6.28E-07

OGs ID	having site under significant positive selection	having site under highly significant positive selection	P value
13Plants09948	✓	✓	6.41E-07
13Plants08280	✓	-	6.81E-07
13Plants08969	✓	✓	6.94E-07
13Plants03760	✓	✓	7.21E-07
13Plants07349	-	✓	7.33E-07
13Plants11398	✓	✓	7.40E-07
13Plants09148	✓	✓	7.46E-07
13Plants12732	✓	-	7.52E-07
13Plants06270	-	✓	7.78E-07
13Plants10204	✓	✓	8.17E-07
13Plants05337	✓	✓	8.65E-07
13Plants07109	✓	✓	8.90E-07
13Plants10498	✓	✓	8.90E-07
13Plants05713	✓	✓	8.97E-07
13Plants08989	✓	✓	9.48E-07
13Plants02478	✓	✓	9.65E-07
13Plants07497	✓	✓	1.00E-06
13Plants04270	✓	✓	1.01E-06
13Plants05205	✓	✓	1.01E-06
13Plants10478	✓	✓	1.01E-06
13Plants05353	✓	✓	1.08E-06
13Plants08383	✓	✓	1.21E-06
13Plants13363	✓	✓	1.25E-06
13Plants03357	✓	✓	1.30E-06
13Plants04188	✓	✓	1.32E-06
13Plants08739	✓	✓	1.33E-06
13Plants10398	✓	✓	1.33E-06
13Plants08554	✓	✓	1.41E-06
13Plants10403	✓	-	1.44E-06
13Plants11568	✓	✓	1.45E-06
13Plants05959	✓	✓	1.48E-06
13Plants07912	-	✓	1.49E-06
13Plants00836	✓	✓	1.61E-06

OGs ID	having site under significant positive selection	having site under highly significant positive selection	P value
13Plants08427	✓	✓	1.68E-06
13Plants04960	✓	✓	1.81E-06
13Plants07140	✓	✓	1.86E-06
13Plants08036	✓	✓	1.96E-06
13Plants07134	-	✓	2.03E-06
13Plants10649	✓	✓	2.05E-06
13Plants06063	✓	-	2.09E-06
13Plants05227	✓	✓	2.17E-06
13Plants11404	✓	✓	2.24E-06
13Plants03880	✓	-	2.57E-06
13Plants04586	✓	✓	2.69E-06
13Plants06382	✓	✓	2.70E-06
13Plants08237	✓	✓	2.75E-06
13Plants07034	-	✓	2.95E-06
13Plants08841	✓	✓	3.01E-06
13Plants10672	✓	-	3.02E-06
13Plants03925	✓	✓	3.06E-06
13Plants05711	✓	✓	3.10E-06
13Plants10656	✓	-	3.21E-06
13Plants10416	✓	✓	3.27E-06
13Plants04035	✓	✓	3.57E-06
13Plants08649	-	✓	4.16E-06
13Plants08544	✓	-	4.18E-06
13Plants04977	✓	✓	4.21E-06
13Plants03554	✓	-	4.31E-06
13Plants09788	✓	✓	4.47E-06
13Plants07494	✓	✓	4.48E-06
13Plants03877	✓	-	5.07E-06
13Plants07033	✓	✓	5.22E-06
13Plants06832	✓	-	5.45E-06
13Plants08156	✓	✓	5.56E-06
13Plants07421	✓	✓	5.57E-06
13Plants05072	✓	✓	5.64E-06

OGs ID	having site under significant positive selection	having site under highly significant positive selection	P value
13Plants06066	✓	✓	5.70E-06
13Plants06740	✓	✓	5.74E-06
13Plants02468	✓	✓	5.75E-06
13Plants09395	✓	✓	5.80E-06
13Plants05772	✓	✓	5.86E-06
13Plants08641	✓	-	5.96E-06
13Plants06892	✓	-	6.00E-06
13Plants11809	✓	-	6.04E-06
13Plants07539	✓	✓	6.43E-06
13Plants09483	-	✓	6.67E-06
13Plants10392	✓	-	6.80E-06
13Plants10679	✓	✓	7.46E-06
13Plants09797	✓	✓	7.48E-06
13Plants04601	✓	-	7.51E-06
13Plants06802	✓	-	7.61E-06
13Plants06193	✓	-	7.80E-06
13Plants10133	-	✓	8.56E-06
13Plants09850	✓	-	8.83E-06
13Plants10665	✓	✓	9.37E-06
13Plants01910	✓	✓	9.48E-06
13Plants03894	✓	✓	1.04E-05
13Plants04650	✓	-	1.05E-05
13Plants10238	✓	✓	1.08E-05
13Plants07226	✓	✓	1.10E-05
13Plants02568	✓	✓	1.11E-05
13Plants08796	✓	✓	1.14E-05
13Plants13881	✓	✓	1.15E-05
13Plants07944	-	✓	1.19E-05
13Plants11525	✓	✓	1.21E-05
13Plants02929	✓	-	1.21E-05
13Plants03407	-	✓	1.25E-05
13Plants08099	✓	✓	1.35E-05
13Plants04050	✓	✓	1.38E-05

OGs ID	having site under significant positive selection	having site under highly significant positive selection	P value
13Plants12560	✓	-	1.44E-05
13Plants05775	-	✓	1.47E-05
13Plants04839	✓	-	1.48E-05
13Plants09110	✓	✓	1.56E-05
13Plants12434	✓	✓	1.63E-05
13Plants03317	-	✓	1.63E-05
13Plants08272	✓	✓	1.70E-05
13Plants08435	✓	✓	1.71E-05
13Plants11913	✓	-	1.85E-05
13Plants10128	✓	-	1.91E-05
13Plants05789	✓	✓	1.92E-05
13Plants10071	✓	✓	2.08E-05
13Plants11123	✓	✓	2.10E-05
13Plants10557	✓	✓	2.21E-05
13Plants07087	✓	✓	2.22E-05
13Plants09955	✓	✓	2.26E-05
13Plants00607	✓	-	2.34E-05
13Plants08141	✓	✓	2.47E-05
13Plants06011	✓	✓	2.52E-05
13Plants05949	✓	✓	2.57E-05
13Plants04459	✓	✓	2.58E-05
13Plants03884	✓	✓	2.59E-05
13Plants05242	-	✓	2.71E-05
13Plants10083	✓	✓	2.83E-05
13Plants04456	✓	✓	2.92E-05
13Plants08676	✓	-	2.92E-05
13Plants08008	-	✓	2.93E-05
13Plants07080	✓	-	2.94E-05
13Plants03084	✓	✓	2.98E-05
13Plants06909	✓	✓	2.99E-05
13Plants05195	✓	✓	3.09E-05
13Plants08428	✓	-	3.14E-05
13Plants06677	✓	✓	3.19E-05

OGs ID	having site under significant positive selection	having site under highly significant positive selection	P value
13Plants04904	✓	✓	3.28E-05
13Plants10621	✓	✓	3.29E-05
13Plants09306	✓	-	3.32E-05
13Plants07973	✓	✓	3.33E-05
13Plants05894	✓	✓	3.33E-05
13Plants07459	✓	✓	3.43E-05
13Plants07099	✓	✓	3.65E-05
13Plants10078	-	✓	3.66E-05
13Plants04647	✓	-	3.72E-05
13Plants00421	-	✓	3.89E-05
13Plants03527	✓	✓	3.97E-05
13Plants04272	✓	✓	4.32E-05
13Plants05984	✓	✓	4.39E-05
13Plants11434	✓	✓	4.58E-05
13Plants09805	✓	✓	4.87E-05
13Plants08640	✓	-	5.00E-05
13Plants10050	✓	-	5.03E-05
13Plants09792	✓	✓	5.13E-05
13Plants11513	✓	✓	5.25E-05
13Plants04928	✓	✓	5.30E-05
13Plants03686	✓	-	5.30E-05
13Plants07615	✓	-	5.40E-05
13Plants10146	✓	✓	5.46E-05
13Plants09551	-	✓	5.46E-05
13Plants03352	✓	-	5.47E-05
13Plants08772	-	✓	5.76E-05
13Plants11567	✓	-	5.86E-05
13Plants07278	✓	✓	5.90E-05
13Plants07300	✓	✓	6.30E-05
13Plants09410	✓	✓	6.37E-05
13Plants10466	✓	-	6.39E-05
13Plants08120	✓	✓	6.50E-05
13Plants08224	-	✓	6.81E-05

OGs ID	having site under significant positive selection	having site under highly significant positive selection	P value
13Plants06830	✓	✓	6.82E-05
13Plants04905	-	✓	6.89E-05
13Plants05284	✓	✓	7.09E-05
13Plants06029	✓	✓	7.18E-05
13Plants08934	✓	-	7.52E-05
13Plants07881	✓	-	7.68E-05
13Plants10324	-	✓	7.75E-05
13Plants09557	✓	✓	7.79E-05
13Plants10433	✓	✓	7.83E-05
13Plants03954	✓	✓	7.85E-05
13Plants02326	✓	-	7.88E-05
13Plants06449	-	✓	8.22E-05
13Plants06699	-	✓	9.08E-05
13Plants01545	✓	✓	9.58E-05
13Plants04871	✓	-	9.69E-05
13Plants14003	✓	✓	9.86E-05
13Plants12636	✓	-	1.01E-04
13Plants09800	-	✓	1.02E-04
13Plants05357	✓	✓	1.04E-04
13Plants04949	✓	✓	1.05E-04
13Plants13087	✓	✓	1.09E-04
13Plants06994	✓	✓	1.10E-04
13Plants08881	✓	-	1.10E-04
13Plants10684	-	✓	1.12E-04
13Plants08183	✓	✓	1.13E-04
13Plants13413	-	✓	1.20E-04
13Plants10363	-	✓	1.21E-04
13Plants05782	✓	-	1.31E-04
13Plants10091	-	✓	1.32E-04
13Plants03483	✓	✓	1.35E-04
13Plants08327	✓	✓	1.36E-04
13Plants06472	✓	✓	1.39E-04
13Plants09649	✓	✓	1.43E-04

OGs ID	having site under significant positive selection	having site under highly significant positive selection	P value
13Plants06115	✓	-	1.43E-04
13Plants04363	✓	✓	1.66E-04
13Plants01871	✓	-	1.69E-04
13Plants02454	✓	✓	1.69E-04
13Plants01607	✓	✓	1.71E-04
13Plants04926	✓	✓	1.72E-04
13Plants07027	✓	✓	1.73E-04
13Plants11417	✓	-	1.89E-04
13Plants05292	✓	-	2.02E-04
13Plants11409	-	✓	2.05E-04
13Plants12120	✓	✓	2.29E-04
13Plants09823	✓	✓	2.36E-04
13Plants07919	✓	✓	2.36E-04
13Plants03095	✓	✓	2.38E-04
13Plants09267	-	✓	2.41E-04
13Plants10671	✓	-	2.51E-04
13Plants10550	✓	✓	2.63E-04
13Plants08852	✓	✓	2.64E-04
13Plants06144	✓	✓	2.73E-04
13Plants07169	-	✓	2.74E-04
13Plants04180	✓	✓	2.78E-04
13Plants06310	✓	✓	2.83E-04
13Plants05034	✓	✓	2.83E-04
13Plants05927	✓	✓	3.05E-04
13Plants08443	✓	✓	3.14E-04
13Plants07883	✓	✓	3.17E-04
13Plants07115	✓	✓	3.21E-04
13Plants11461	✓	-	3.24E-04
13Plants10286	✓	✓	3.25E-04
13Plants03184	✓	-	3.42E-04
13Plants08590	✓	✓	3.49E-04
13Plants03293	✓	-	3.60E-04
13Plants03555	-	✓	3.62E-04

OGs ID	having site under significant positive selection	having site under highly significant positive selection	P value
13Plants03836	✓	✓	3.65E-04
13Plants06178	✓	✓	3.86E-04
13Plants07276	-	✓	3.96E-04
13Plants08802	-	✓	4.07E-04
13Plants02767	✓	✓	4.15E-04
13Plants06713	✓	✓	4.16E-04
13Plants12496	✓	-	4.20E-04
13Plants08588	✓	✓	4.32E-04
13Plants09875	✓	-	4.34E-04
13Plants10432	✓	-	4.40E-04
13Plants06913	✓	✓	4.45E-04
13Plants08808	-	✓	4.61E-04
13Plants11093	-	✓	4.65E-04
13Plants08241	✓	-	4.79E-04
13Plants10420	✓	-	4.93E-04
13Plants10289	✓	✓	4.96E-04
13Plants08735	✓	-	4.97E-04
13Plants09904	-	✓	5.06E-04
13Plants01796	✓	✓	5.12E-04
13Plants05305	✓	✓	5.21E-04
13Plants11173	✓	-	5.38E-04
13Plants11523	-	✓	5.39E-04
13Plants10056	✓	✓	5.57E-04
13Plants05873	✓	-	5.57E-04
13Plants02859	✓	-	5.59E-04
13Plants06009	✓	✓	5.75E-04
13Plants07189	-	✓	5.93E-04
13Plants09986	✓	-	6.09E-04
13Plants08665	✓	✓	6.16E-04
13Plants03032	✓	-	6.26E-04
13Plants07892	✓	✓	6.44E-04
13Plants02347	✓	✓	6.64E-04
13Plants03411	-	✓	6.64E-04

OGs ID	having site under significant positive selection	having site under highly significant positive selection	P value
13Plants05780	-	✓	6.66E-04
13Plants10316	✓	-	6.67E-04
13Plants09530	-	✓	6.96E-04
13Plants10348	✓	✓	7.30E-04
13Plants10065	✓	-	7.45E-04
13Plants05162	✓	✓	7.48E-04
13Plants10613	✓	-	7.65E-04
13Plants08511	✓	-	7.68E-04
13Plants05244	✓	✓	7.72E-04
13Plants01611	✓	-	7.87E-04
13Plants08056	✓	-	7.88E-04
13Plants11631	✓	✓	7.97E-04
13Plants11874	✓	✓	8.11E-04
13Plants01451	-	✓	8.25E-04
13Plants13830	✓	✓	8.27E-04
13Plants11275	✓	✓	8.36E-04
13Plants09791	✓	-	8.49E-04
13Plants02997	✓	✓	8.79E-04
13Plants10293	✓	-	8.81E-04
13Plants05794	✓	-	8.86E-04
13Plants04634	✓	✓	8.88E-04
13Plants07269	✓	✓	9.00E-04
13Plants06246	✓	✓	9.24E-04
13Plants13929	✓	-	9.25E-04
13Plants11063	✓	✓	9.59E-04
13Plants10144	✓	-	9.81E-04
13Plants06147	✓	✓	9.92E-04
13Plants08848	✓	✓	1.03E-03
13Plants10616	-	✓	1.03E-03
13Plants02753	✓	-	1.07E-03
13Plants13210	✓	-	1.09E-03
13Plants05950	✓	✓	1.10E-03
13Plants02480	✓	✓	1.10E-03

OGs ID	having site under significant positive selection	having site under highly significant positive selection	P value
13Plants06451	✓	-	1.14E-03
13Plants03800	✓	-	1.14E-03
13Plants05256	✓	✓	1.16E-03
13Plants08274	✓	✓	1.16E-03
13Plants08910	✓	✓	1.16E-03
13Plants04418	✓	-	1.18E-03
13Plants06167	✓	-	1.18E-03
13Plants04899	✓	✓	1.21E-03
13Plants10568	✓	✓	1.26E-03
13Plants10090	✓	-	1.31E-03
13Plants04465	✓	-	1.35E-03
13Plants08669	✓	✓	1.36E-03
13Plants08567	✓	-	1.38E-03
13Plants13965	-	✓	1.44E-03
13Plants12811	✓	-	1.48E-03
13Plants11047	-	✓	1.49E-03
13Plants06237	✓	✓	1.49E-03
13Plants09671	✓	-	1.60E-03
13Plants06705	✓	-	1.62E-03
13Plants12207	✓	-	1.65E-03
13Plants06372	✓	✓	1.68E-03
13Plants06829	✓	-	1.71E-03
13Plants14005	✓	✓	1.71E-03
13Plants11095	✓	-	1.74E-03
13Plants09771	✓	-	1.76E-03
13Plants09937	✓	✓	1.77E-03
13Plants11320	✓	-	1.80E-03
13Plants09413	✓	-	1.80E-03
13Plants09668	✓	-	1.81E-03
13Plants08143	✓	✓	1.87E-03
13Plants03183	✓	-	1.89E-03
13Plants04969	✓	✓	1.95E-03
13Plants06255	-	✓	1.96E-03

OGs ID	having site under significant positive selection	having site under highly significant positive selection	P value
13Plants09294	✓	-	1.98E-03
13Plants03294	✓	-	1.98E-03
13Plants12758	✓	✓	1.99E-03
13Plants11478	✓	✓	2.02E-03
13Plants06730	✓	-	2.03E-03
13Plants05739	✓	-	2.04E-03
13Plants05972	✓	-	2.06E-03
13Plants07030	✓	✓	2.06E-03
13Plants10210	✓	✓	2.11E-03
13Plants05947	✓	-	2.17E-03
13Plants15485	✓	-	2.19E-03
13Plants08476	-	✓	2.21E-03
13Plants14377	✓	-	2.29E-03
13Plants07506	✓	-	2.35E-03
13Plants10044	✓	✓	2.38E-03
13Plants07245	-	✓	2.43E-03
13Plants06847	✓	-	2.44E-03
13Plants07017	✓	-	2.49E-03
13Plants06241	✓	✓	2.50E-03
13Plants10575	✓	-	2.51E-03
13Plants08144	✓	✓	2.52E-03
13Plants10449	✓	-	2.63E-03
13Plants09026	✓	✓	2.65E-03
13Plants06809	-	✓	2.69E-03
13Plants09603	✓	-	2.73E-03
13Plants05996	✓	-	2.79E-03
13Plants12004	✓	✓	2.85E-03
13Plants13088	✓	✓	2.86E-03
13Plants08770	✓	✓	2.87E-03
13Plants11447	✓	✓	2.94E-03
13Plants11082	-	✓	3.18E-03
13Plants06097	✓	✓	3.18E-03
13Plants07931	✓	-	3.21E-03

OGs ID	having site under significant positive selection	having site under highly significant positive selection	P value
13Plants04219	✓	✓	3.23E-03
13Plants09589	✓	-	3.23E-03
13Plants03523	✓	✓	3.29E-03
13Plants06820	-	✓	3.42E-03
13Plants13061	✓	-	3.45E-03
13Plants09573	✓	-	3.45E-03
13Plants06176	✓	-	3.50E-03
13Plants11564	✓	✓	3.51E-03
13Plants08145	✓	-	3.61E-03
13Plants08404	✓	✓	3.74E-03
13Plants02068	✓	✓	3.74E-03
13Plants05923	-	✓	3.75E-03
13Plants03120	✓	-	3.76E-03
13Plants03761	✓	✓	3.93E-03
13Plants03218	✓	-	4.33E-03
13Plants06770	✓	-	4.36E-03
13Plants04211	-	✓	4.37E-03
13Plants10194	✓	-	4.37E-03
13Plants05717	✓	✓	4.38E-03
13Plants04690	✓	-	4.47E-03
13Plants07518	✓	-	4.62E-03
13Plants03549	✓	-	4.73E-03
13Plants10362	✓	-	4.73E-03
13Plants10284	-	✓	4.81E-03
13Plants08948	✓	-	4.81E-03
13Plants01347	✓	✓	4.82E-03
13Plants07263	✓	✓	5.02E-03
13Plants02482	-	✓	5.02E-03
13Plants08331	-	✓	5.07E-03
13Plants08913	✓	-	5.08E-03
13Plants03113	✓	✓	5.09E-03
13Plants06347	✓	✓	5.14E-03
13Plants04604	✓	✓	5.15E-03

OGs ID	having site under significant positive selection	having site under highly significant positive selection	P value
13Plants10072	✓	✓	5.22E-03
13Plants10476	✓	-	5.35E-03
13Plants10538	✓	-	5.56E-03
13Plants04631	✓	-	5.61E-03
13Plants03355	✓	-	5.63E-03
13Plants10172	✓	-	5.70E-03
13Plants04431	✓	-	5.72E-03
13Plants07479	✓	✓	6.10E-03
13Plants07488	✓	-	6.15E-03
13Plants05028	-	✓	6.17E-03
13Plants05327	✓	-	6.19E-03
13Plants09160	✓	-	6.23E-03
13Plants14447	✓	✓	6.25E-03
13Plants03468	✓	-	6.37E-03
13Plants06906	✓	✓	6.59E-03
13Plants05953	✓	-	6.59E-03
13Plants08416	✓	✓	6.65E-03
13Plants07074	✓	-	6.72E-03
13Plants02563	✓	-	6.87E-03
13Plants01175	✓	✓	7.00E-03
13Plants02128	✓	-	7.06E-03
13Plants01683	-	✓	7.14E-03
13Plants03460	✓	-	7.25E-03
13Plants05298	✓	-	7.28E-03
13Plants10642	✓	-	7.29E-03
13Plants06746	✓	-	7.36E-03
13Plants08614	✓	-	7.40E-03
13Plants10641	✓	-	7.51E-03
13Plants10405	✓	✓	7.61E-03
13Plants07627	✓	-	7.71E-03
13Plants08645	✓	✓	7.75E-03
13Plants06380	✓	-	7.79E-03
13Plants05844	✓	-	7.86E-03

OGs ID	having site under significant positive selection	having site under highly significant positive selection	P value
13Plants07366	✓	✓	7.97E-03
13Plants03697	-	✓	8.03E-03
13Plants12105	✓	-	8.13E-03
13Plants13997	✓	-	8.13E-03
13Plants12762	✓	-	8.42E-03
13Plants05834	✓	✓	8.54E-03
13Plants07623	✓	-	8.60E-03
13Plants10506	✓	-	8.72E-03
13Plants06692	-	✓	8.73E-03
13Plants10156	✓	-	8.74E-03
13Plants07678	✓	-	8.83E-03
13Plants09279	✓	-	8.86E-03
13Plants10710	✓	-	8.88E-03
13Plants08247	✓	✓	8.91E-03
13Plants00280	✓	-	9.18E-03
13Plants11565	✓	✓	9.25E-03
13Plants14033	✓	✓	9.42E-03
13Plants10148	✓	-	9.49E-03
13Plants04242	-	✓	9.66E-03
13Plants10509	-	✓	9.81E-03
13Plants12478	✓	✓	9.93E-03
13Plants10015	✓	-	1.01E-02
13Plants02397	✓	-	1.01E-02
13Plants05705	✓	-	1.01E-02
13Plants05799	✓	-	1.03E-02
13Plants11405	✓	✓	1.03E-02
13Plants07490	✓	-	1.03E-02
13Plants07177	✓	-	1.04E-02
13Plants10777	-	✓	1.05E-02
13Plants06941	✓	-	1.06E-02
13Plants06860	-	✓	1.07E-02
13Plants02239	✓	-	1.07E-02
13Plants10395	✓	-	1.07E-02

OGs ID	having site under significant positive selection	having site under highly significant positive selection	P value
13Plants05167	✓	✓	1.08E-02
13Plants11976	✓	-	1.09E-02
13Plants01577	-	✓	1.09E-02
13Plants11026	✓	-	1.11E-02
13Plants12575	✓	✓	1.11E-02
13Plants10107	✓	✓	1.12E-02
13Plants08377	-	✓	1.12E-02
13Plants05012	✓	-	1.13E-02
13Plants09456	✓	-	1.15E-02
13Plants10141	✓	-	1.15E-02
13Plants08856	-	✓	1.25E-02
13Plants02307	✓	✓	1.30E-02
13Plants09303	✓	-	1.30E-02
13Plants07121	✓	✓	1.31E-02
13Plants03576	✓	-	1.31E-02
13Plants08686	✓	-	1.34E-02
13Plants09009	✓	✓	1.34E-02
13Plants07535	✓	-	1.35E-02
13Plants10074	✓	-	1.35E-02
13Plants12618	✓	-	1.38E-02
13Plants06173	✓	-	1.39E-02
13Plants08270	-	✓	1.39E-02
13Plants07029	✓	✓	1.39E-02
13Plants04277	✓	-	1.42E-02
13Plants02829	✓	-	1.42E-02
13Plants02489	✓	-	1.44E-02
13Plants03163	✓	-	1.46E-02
13Plants10999	✓	-	1.49E-02
13Plants08177	✓	✓	1.49E-02
13Plants02532	✓	✓	1.50E-02
13Plants13202	✓	-	1.51E-02
13Plants05689	✓	-	1.55E-02
13Plants06195	✓	-	1.63E-02

OGs ID	having site under significant positive selection	having site under highly significant positive selection	P value
13Plants05774	✓	-	1.65E-02
13Plants12666	✓	✓	1.66E-02
13Plants03756	✓	-	1.66E-02
13Plants07621	✓	-	1.70E-02
13Plants05833	-	✓	1.75E-02
13Plants05249	✓	-	1.79E-02
13Plants05424	✓	✓	1.79E-02
13Plants02073	✓	-	1.82E-02
13Plants14019	✓	-	1.82E-02
13Plants06753	✓	-	1.84E-02
13Plants03938	-	✓	1.86E-02
13Plants10571	-	✓	1.91E-02
13Plants12580	✓	-	1.92E-02
13Plants08643	✓	-	1.92E-02
13Plants10410	✓	✓	1.99E-02
13Plants06673	✓	-	2.00E-02
13Plants06411	-	✓	2.01E-02
13Plants04457	✓	✓	2.03E-02
13Plants06400	✓	✓	2.03E-02
13Plants03798	✓	✓	2.04E-02
13Plants07303	✓	-	2.04E-02
13Plants09780	✓	-	2.10E-02
13Plants05698	✓	-	2.15E-02
13Plants04421	✓	-	2.16E-02
13Plants02879	✓	-	2.24E-02
13Plants08514	-	✓	2.25E-02
13Plants04373	✓	✓	2.27E-02
13Plants02869	✓	-	2.28E-02
13Plants10502	✓	-	2.32E-02
13Plants01912	-	✓	2.34E-02
13Plants03347	✓	-	2.34E-02
13Plants04258	✓	-	2.34E-02
13Plants07579	✓	-	2.34E-02

OGs ID	having site under significant positive selection	having site under highly significant positive selection	P value
13Plants11270	✓	-	2.35E-02
13Plants05969	✓	-	2.37E-02
13Plants03334	✓	-	2.39E-02
13Plants11440	✓	-	2.41E-02
13Plants11928	✓	-	2.45E-02
13Plants04212	✓	-	2.47E-02
13Plants12002	✓	-	2.47E-02
13Plants03748	✓	-	2.49E-02
13Plants10319	✓	-	2.49E-02
13Plants14450	✓	-	2.51E-02
13Plants08474	✓	✓	2.54E-02
13Plants13419	✓	-	2.57E-02
13Plants05858	✓	-	2.58E-02
13Plants05735	✓	✓	2.58E-02
13Plants08533	✓	-	2.61E-02
13Plants05758	✓	-	2.62E-02
13Plants05190	✓	-	2.66E-02
13Plants05468	✓	✓	2.66E-02
13Plants06939	✓	-	2.68E-02
13Plants06697	✓	✓	2.70E-02
13Plants08754	✓	-	2.72E-02
13Plants09672	✓	-	2.76E-02
13Plants11350	✓	-	2.80E-02
13Plants04028	✓	-	2.83E-02
13Plants04651	-	✓	2.87E-02
13Plants15420	✓	-	2.88E-02
13Plants11495	✓	-	2.90E-02
13Plants10596	✓	-	2.95E-02
13Plants03498	✓	-	2.95E-02
13Plants05018	✓	-	2.96E-02
13Plants06454	-	✓	2.98E-02
13Plants13152	✓	-	3.00E-02
13Plants03899	✓	-	3.00E-02

OGs ID	having site under significant positive selection	having site under highly significant positive selection	P value
13Plants08085	✓	-	3.01E-02
13Plants04462	✓	-	3.03E-02
13Plants06649	✓	-	3.13E-02
13Plants06470	✓	-	3.17E-02
13Plants09883	-	✓	3.19E-02
13Plants10578	✓	-	3.22E-02
13Plants07636	✓	-	3.25E-02
13Plants12594	✓	-	3.35E-02
13Plants11362	✓	✓	3.35E-02
13Plants10206	✓	-	3.38E-02
13Plants04411	✓	-	3.44E-02
13Plants06184	✓	-	3.44E-02
13Plants08939	-	✓	3.52E-02
13Plants07282	✓	-	3.57E-02
13Plants06946	✓	-	3.58E-02
13Plants08642	✓	-	3.63E-02
13Plants08886	✓	-	3.64E-02
13Plants05976	✓	-	3.70E-02
13Plants13416	✓	-	3.73E-02
13Plants03284	✓	✓	3.76E-02
13Plants09772	✓	-	3.78E-02
13Plants01909	✓	-	3.81E-02
13Plants03031	✓	-	3.84E-02
13Plants09909	-	✓	3.84E-02
13Plants12500	✓	-	3.87E-02
13Plants08570	✓	-	3.87E-02
13Plants02722	✓	-	4.05E-02
13Plants07104	-	✓	4.22E-02
13Plants08444	✓	-	4.25E-02
13Plants02007	✓	-	4.27E-02
13Plants01335	-	✓	4.29E-02
13Plants05027	✓	-	4.32E-02
13Plants04589	✓	✓	4.39E-02

OGs ID	having site under significant positive selection	having site under highly significant positive selection	P value
13Plants02792	✓	-	4.39E-02
13Plants05286	✓	✓	4.44E-02
13Plants08276	✓	-	4.58E-02
13Plants10522	✓	-	4.61E-02
13Plants00766	✓	-	4.64E-02
13Plants05886	✓	-	4.66E-02
13Plants03742	✓	-	4.66E-02
13Plants15388	✓	-	4.72E-02
13Plants05401	✓	-	4.81E-02
13Plants05819	✓	-	4.84E-02
13Plants03552	✓	✓	4.88E-02
13Plants02836	✓	-	4.88E-02
13Plants13214	✓	-	4.91E-02
13Plants04850	-	✓	4.93E-02

Note: In the table, "having site under significant positive selection" means p-value < 0.05 using the Bayes Empirical Bayes (BEB) method, and "having site under highly significant positive selection" means p-value < 0.01 using the BEB method.

Table S10. 181 PSGs specifically identified in *A. catalpifolium*.

Gene IDs				
EVM0012930.1	*EVM0031507.1*	*EVM0021576.2*	*EVM0010754.1*	*EVM0009703.1*
EVM0008653.1	*EVM0019210.1*	*EVM0014280.1*	*EVM0007459.1*	*EVM0020673.1*
EVM0021364.1	*EVM0002740.1*	*EVM0032996.1*	*EVM0016838.1*	*EVM0026867.1*
EVM0011209.1	*EVM0010645.1*	*EVM0034384.1*	*EVM0012148.2*	*EVM0030108.1*
EVM0020264.1	*EVM0025380.1*	*EVM0014748.1*	*EVM0034894.1*	*EVM0032251.1*
EVM0030982.1	*EVM0013769.1*	*EVM0025240.1*	*EVM0027547.1*	*EVM0001945.1*
EVM0023792.1	*EVM0019076.1*	*EVM0032840.1*	*EVM0000752.1*	*EVM0014076.1*
EVM0012856.1	*EVM0027459.1*	*EVM0010018.1*	*EVM0031260.1*	*EVM0005367.1*
EVM0031875.1	*EVM0020196.1*	*EVM0000433.1*	*EVM0025988.1*	*EVM0008503.1*
EVM0033854.1	*EVM0030082.1*	*EVM0012189.1*	*EVM0001197.1*	*EVM0028806.1*
EVM0024223.1	*EVM0007201.1*	*EVM0031452.3*	*EVM0031821.1*	*EVM0013030.1*
EVM0005784.1	*EVM0027710.1*	*EVM0033639.1*	*EVM0012352.1*	*EVM0023131.1*
EVM0006903.1	*EVM0028112.1*	*EVM0015722.1*	*EVM0018728.1*	*EVM0033033.1*
EVM0021587.1	*EVM0019469.1*	*EVM0024710.3*	*EVM0023868.1*	*EVM0024339.1*
EVM0026574.1	*EVM0005045.1*	*EVM0022732.1*	*EVM0015819.1*	*EVM0027528.2*
EVM0020742.1	*EVM0009137.1*	*EVM0022048.1*	*EVM0027943.1*	*EVM0020238.1*
EVM0019368.1	*EVM0024427.1*	*EVM0022703.1*	*EVM0002294.1*	*EVM0020085.1*
EVM0020912.1	*EVM0003085.1*	*EVM0001837.1*	*EVM0030735.4*	*EVM0008137.1*
EVM0027504.1	*EVM0024445.1*	*EVM0007850.1*	*EVM0025250.1*	*EVM0024450.1*
EVM0017477.1	*EVM0031137.1*	*EVM0029636.1*	*EVM0003459.2*	*EVM0028683.3*
EVM0009518.1	*EVM0027978.1*	*EVM0021971.1*	*EVM0006780.1*	*EVM0014193.1*
EVM0025599.1	*EVM0002340.1*	*EVM0002118.1*	*EVM0029527.1*	*EVM0002773.1*
EVM0020448.1	*EVM0005671.1*	*EVM0002828.1*	*EVM0014112.1*	*EVM0021321.1*
EVM0013074.1	*EVM0011571.1*	*EVM0019353.1*	*EVM0030338.1*	*EVM0031799.1*
EVM0020485.1	*EVM0008183.1*	*EVM0024963.1*	*EVM0025934.1*	*EVM0005492.1*
EVM0018350.1	*EVM0014390.2*	*EVM0002380.1*	*EVM0020457.1*	*EVM0025839.1*
EVM0028058.1	*EVM0007117.1*	*EVM0010467.1*	*EVM0022691.1*	*EVM0005772.1*
EVM0019806.1	*EVM0010057.1*	*EVM0022718.1*	*EVM0025125.1*	*EVM0031330.1*
EVM0014130.1	*EVM0018138.1*	*EVM0007281.2*	*EVM0032895.2*	*EVM0006135.1*
EVM0033698.1	*EVM0017544.1*	*EVM0031892.1*	*EVM0005364.2*	*EVM0019482.1*
EVM0025989.1	*EVM0024995.1*	*EVM0023460.1*	*EVM0021485.2*	*EVM0028239.1*
EVM0014154.1	*EVM0032945.1*	*EVM0024178.1*	*EVM0035172.1*	*EVM0033830.1*
EVM0009012.1	*EVM0005138.1*	*EVM0028870.1*	*EVM0011679.1*	*EVM0023802.1*
EVM0025571.1	*EVM0011267.1*	*EVM0004226.1*	*EVM0005547.1*	
EVM0018272.1	*EVM0031973.1*	*EVM0032047.1*	*EVM0034818.1*	
EVM0027988.1	*EVM0023442.1*	*EVM0003525.3*	*EVM0020431.1*	
EVM0035001.2	*EVM0024334.1*	*EVM0009058.1*	*EVM0016421.1*	

Table S11. 548 PSGs were specifically identified in *A. yangbiense*.

Gene IDs			
Acyan06G0109600.1	*Acyan01G0186100.1*	*Acyan01G0067500.1*	*Acyan08G0120700.1*
Acyan09G0061100.1	*Acyan01G0220600.1*	*Acyan02G0098100.1*	*Acyan01G0236200.1*
Acyan03G0166400.1	*Acyan07G0081400.1*	*Acyan01G0014800.1*	*Acyan08G0039200.1*
Acyan08G0008200.1	*Acyan12G0132500.1*	*Acyan04G0131500.1*	*Acyan07G0169600.1*
Acyan07G0099100.1	*Acyan12G0076600.1*	*Acyan01G0005500.1*	*Acyan12G0072100.1*
Acyan07G0109300.1	*Acyan06G0133900.1*	*Acyan08G0165000.1*	*Acyan01G0240000.1*
Acyan09G0070200.1	*Acyan13G0124000.1*	*Acyan04G0253300.1*	*Acyan05G0062100.1*
Acyan10G0013400.1	*Acyan12G0156300.1*	*Acyan07G0084600.1*	*Acyan07G0016800.1*
Acyan07G0010300.1	*Acyan09G0109700.1*	*Acyan04G0121000.1*	*Acyan01G0171100.1*
Acyan01G0222400.1	*Acyan05G0010000.1*	*Acyan07G0087200.1*	*Acyan11G0059500.1*
Acyan12G0084600.1	*Acyan13G0058400.1*	*Acyan13G0051100.1*	*Acyan12G0145800.1*
Acyan06G0042700.1	*Acyan08G0063900.1*	*Acyan11G0032700.1*	*Acyan08G0003000.1*
Acyan06G0024100.1	*Acyan08G0115100.1*	*Acyan03G0217500.1*	*Acyan13G0126000.1*
Acyan09G0202700.1	*Acyan10G0132900.1*	*Acyan04G0212300.1*	*Acyan10G0141100.1*
Acyan04G0248200.1	*Acyan05G0133500.1*	*Acyan03G0220300.1*	*Acyan03G0091300.1*
Acyan02G0282700.1	*Acyan08G0105600.1*	*Acyan13G0178500.1*	*Acyan03G0117000.1*
Acyan01G0168900.1	*Acyan12G0066600.1*	*Acyan06G0094600.1*	*Acyan05G0082800.1*
Acyan11G0060300.1	*Acyan12G0158200.1*	*Acyan05G0139500.1*	*Acyan09G0117900.1*
Acyan09G0097400.1	*Acyan03G0176400.1*	*Acyan09G0103300.1*	*Acyan07G0012200.1*
Acyan05G0180100.1	*Acyan03G0318800.1*	*Acyan02G0126100.1*	*Acyan05G0150900.1*
Acyan08G0033900.1	*Acyan04G0035000.1*	*Acyan03G0093200.1*	*Acyan13G0084400.1*
Acyan02G0004900.1	*Acyan04G0129300.1*	*Acyan10G0160300.1*	*Acyan05G0095600.1*
Acyan13G0083700.1	*Acyan04G0138200.1*	*Acyan10G0023400.1*	*Acyan08G0067800.1*
Acyan02G0126600.1	*Acyan04G0176000.1*	*Acyan08G0009800.1*	*Acyan10G0110300.1*
Acyan04G0106100.1	*Acyan04G0193300.1*	*Acyan02G0194800.1*	*Acyan01G0145400.1*
Acyan04G0141100.1	*Acyan07G0086300.1*	*Acyan02G0327300.1*	*Acyan04G0077500.1*
Acyan13G0136000.1	*Acyan09G0066700.1*	*Acyan06G0012700.1*	*Acyan03G0166500.1*
Acyan10G0149700.1	*Acyan03G0268500.1*	*Acyan04G0281300.1*	*Acyan01G0147300.1*
Acyan09G0144400.1	*Acyan06G0044600.1*	*Acyan03G0305000.1*	*Acyan02G0149900.1*
Acyan04G0173900.1	*Acyan06G0017500.1*	*Acyan01G0007900.1*	*Acyan12G0082100.1*
Acyan03G0125300.1	*Acyan08G0167100.1*	*Acyan12G0099300.1*	*Acyan12G0082400.1*
Acyan03G0182400.1	*Acyan08G0023900.1*	*Acyan05G0091800.1*	*Acyan01G0244400.1*
Acyan07G0004600.1	*Acyan01G0031300.1*	*Acyan08G0156700.1*	*Acyan09G0027100.1*
Acyan08G0159200.1	*Acyan01G0175100.1*	*Acyan05G0175000.1*	*Acyan01G0184500.1*
Acyan05G0069500.1	*Acyan01G0255600.1*	*Acyan01G0032400.1*	*Acyan07G0049500.1*
Acyan13G0131600.1	*Acyan13G0124100.1*	*Acyan12G0064400.1*	*Acyan11G0061700.1*
Acyan02G0131000.1	*Acyan02G0189400.1*	*Acyan02G0197800.1*	*Acyan07G0155800.1*
Acyan04G0072200.1	*Acyan02G0186300.1*	*Acyan12G0006900.1*	*Acyan09G0120600.1*
Acyan09G0234500.1	*Acyan02G0155900.1*	*Acyan08G0007200.1*	*Acyan01G0243800.1*
Acyan01G0148000.1	*Acyan12G0039700.1*	*Acyan04G0142200.1*	*Acyan02G0266500.1*
Acyan03G0280300.1	*Acyan05G0144700.1*	*Acyan01G0016900.1*	*Acyan11G0152600.1*
Acyan02G0010900.1	*Acyan12G0108900.1*	*Acyan03G0103600.1*	*Acyan02G0135000.1*
Acyan10G0079100.1	*Acyan04G0151800.1*	*Acyan08G0151100.1*	*Acyan13G0167700.1*
Acyan07G0115900.1	*Acyan11G0164100.1*	*Acyan10G0058500.1*	*Acyan08G0051900.1*
Acyan09G0204600.1	*Acyan08G0099600.1*	*Acyan12G0163300.1*	*Acyan07G0021000.1*
Acyan04G0169800.1	*Acyan01G0306300.1*	*Acyan03G0318000.1*	*Acyan01G0071800.1*
Acyan09G0226100.1	*Acyan13G0173000.1*	*Acyan04G0152000.1*	*Acyan11G0042400.1*
Acyan08G0067400.1	*Acyan07G0152900.1*	*Acyan10G0046700.1*	*Acyan09G0161800.1*
Acyan01G0070500.1	*Acyan06G0070600.1*	*Acyan05G0028200.1*	*Acyan03G0132300.1*
Acyan05G0071000.1	*Acyan01G0112700.1*	*Acyan11G0041200.1*	*Acyan08G0179000.1*
Acyan10G0076000.1	*Acyan05G0004500.1*	*Acyan03G0325700.1*	*Acyan01G0046100.1*
Acyan01G0115000.1	*Acyan05G0147500.1*	*Acyan04G0142000.1*	*Acyan08G0127300.1*
Acyan11G0140400.1	*Acyan01G0037500.1*	*Acyan02G0008600.1*	*Acyan11G0044000.1*

Gene IDs			
Acyan12G0035000.1	*Acyan07G0043300.1*	*Acyan05G0041200.1*	*Acyan12G0054600.1*
Acyan01G0096600.1	*Acyan08G0005500.1*	*Acyan03G0180200.1*	*Acyan04G0017800.1*
Acyan01G0032700.1	*Acyan01G0180900.1*	*Acyan05G0165700.1*	*Acyan03G0156300.1*
Acyan02G0171800.1	*Acyan02G0070100.1*	*Acyan05G0032200.1*	*Acyan08G0018600.1*
Acyan02G0309700.1	*Acyan01G0208300.1*	*Acyan01G0069000.1*	*Acyan10G0131800.1*
Acyan09G0140700.1	*Acyan05G0181000.1*	*Acyan02G0109900.1*	*Acyan07G0075300.1*
Acyan12G0055700.1	*Acyan08G0135400.1*	*Acyan01G0264600.1*	*Acyan03G0129700.1*
Acyan10G0124500.1	*Acyan06G0015100.1*	*Acyan02G0177400.1*	*Acyan10G0126500.1*
Acyan02G0177500.1	*Acyan11G0147700.1*	*Acyan09G0127100.1*	*Acyan10G0109200.1*
Acyan11G0010600.1	*Acyan06G0125100.1*	*Acyan03G0316200.1*	*Acyan05G0149400.1*
Acyan06G0103600.1	*Acyan13G0037600.1*	*Acyan11G0053700.1*	*Acyan05G0140300.1*
Acyan10G0090200.1	*Acyan05G0031500.1*	*Acyan11G0044200.1*	*Acyan05G0117400.1*
Acyan08G0065800.1	*Acyan12G0001300.1*	*Acyan11G0096700.1*	*Acyan05G0090600.1*
Acyan01G0072100.1	*Acyan12G0027700.1*	*Acyan07G0087100.1*	*Acyan05G0081000.1*
Acyan01G0068400.1	*Acyan12G0156700.1*	*Acyan03G0262100.1*	*Acyan05G0070700.1*
Acyan08G0006800.1	*Acyan05G0068900.1*	*Acyan04G0169300.1*	*Acyan05G0034000.1*
Acyan10G0023200.1	*Acyan11G0124200.1*	*Acyan06G0152500.1*	*Acyan11G0083600.1*
Acyan02G0244200.1	*Acyan10G0092500.1*	*Acyan10G0025400.1*	*Acyan11G0094200.1*
Acyan09G0120100.1	*Acyan05G0074700.1*	*Acyan10G0026800.1*	*Acyan11G0099900.1*
Acyan09G0200700.1	*Acyan03G0172600.1*	*Acyan10G0027900.1*	*Acyan11G0116500.1*
Acyan01G0217400.1	*Acyan01G0022700.1*	*Acyan04G0143600.1*	*Acyan11G0145100.1*
Acyan03G0216100.1	*Acyan04G0002400.1*	*Acyan04G0028200.1*	*Acyan12G0093800.1*
Acyan06G0117000.1	*Acyan07G0164100.1*	*Acyan04G0082800.1*	*Acyan03G0160400.1*
Acyan04G0047200.1	*Acyan10G0009900.1*	*Acyan09G0068500.1*	*Acyan03G0187900.1*
Acyan04G0142500.1	*Acyan10G0063100.1*	*Acyan07G0042600.1*	*Acyan06G0056100.1*
Acyan01G0249500.1	*Acyan09G0208400.1*	*Acyan07G0051400.1*	*Acyan03G0314400.1*
Acyan05G0117200.1	*Acyan07G0174400.1*	*Acyan04G0211100.1*	*Acyan04G0025200.1*
Acyan03G0019700.1	*Acyan13G0176500.1*	*Acyan03G0054900.1*	*Acyan04G0026400.1*
Acyan08G0034200.1	*Acyan09G0125900.1*	*Acyan06G0059800.1*	*Acyan04G0087100.1*
Acyan03G0011800.1	*Acyan03G0177100.1*	*Acyan02G0236000.1*	*Acyan04G0091900.1*
Acyan02G0160200.1	*Acyan03G0146200.1*	*Acyan05G0024000.1*	*Acyan04G0130300.1*
Acyan04G0077800.1	*Acyan03G0023500.1*	*Acyan05G0024600.1*	*Acyan04G0161000.1*
Acyan08G0033700.1	*Acyan11G0060400.1*	*Acyan03G0131700.1*	*Acyan06G0018400.1*
Acyan09G0159600.1	*Acyan05G0177600.1*	*Acyan06G0128200.1*	*Acyan07G0123100.1*
Acyan12G0159500.1	*Acyan10G0121000.1*	*Acyan01G0150200.1*	*Acyan07G0082000.1*
Acyan07G0142900.1	*Acyan04G0099800.1*	*Acyan01G0143600.1*	*Acyan07G0065800.1*
Acyan11G0043100.1	*Acyan04G0224900.1*	*Acyan02G0150400.1*	*Acyan09G0217000.1*
Acyan03G0075200.1	*Acyan01G0221100.1*	*Acyan12G0097700.1*	*Acyan09G0212500.1*
Acyan02G0329000.1	*Acyan05G0120300.1*	*Acyan03G0113300.1*	*Acyan09G0083900.1*
Acyan01G0202300.1	*Acyan02G0005300.1*	*Acyan06G0141300.1*	*Acyan09G0082500.1*
Acyan02G0118700.1	*Acyan09G0108100.1*	*Acyan12G0029000.1*	*Acyan09G0076600.1*
Acyan02G0183900.1	*Acyan05G0063500.1*	*Acyan03G0237100.1*	*Acyan09G0032400.1*
Acyan08G0131700.1	*Acyan04G0247000.1*	*Acyan11G0096900.1*	*Acyan06G0143400.1*
Acyan04G0146000.1	*Acyan03G0307900.1*	*Acyan09G0047100.1*	*Acyan06G0092400.1*
Acyan04G0099500.1	*Acyan04G0079200.1*	*Acyan05G0175600.1*	*Acyan06G0010400.1*
Acyan09G0175600.1	*Acyan03G0216200.1*	*Acyan11G0148000.1*	*Acyan08G0141800.1*
Acyan08G0070400.1	*Acyan01G0195700.1*	*Acyan04G0026200.1*	*Acyan08G0130300.1*
Acyan10G0161700.1	*Acyan03G0087100.1*	*Acyan10G0118100.1*	*Acyan08G0110600.1*
Acyan12G0139300.1	*Acyan03G0194900.1*	*Acyan01G0127500.1*	*Acyan08G0104400.1*
Acyan04G0145700.1	*Acyan06G0053900.1*	*Acyan02G0204000.1*	*Acyan08G0097300.1*
Acyan05G0027800.1	*Acyan11G0043300.1*	*Acyan10G0137700.1*	*Acyan08G0072000.1*
Acyan01G0262900.1	*Acyan13G0087500.1*	*Acyan04G0181400.1*	*Acyan08G0061800.1*
Acyan07G0060600.1	*Acyan12G0025900.1*	*Acyan02G0094400.1*	*Acyan01G0018700.1*
Acyan06G0156600.1	*Acyan12G0053400.1*	*Acyan02G0302400.1*	*Acyan01G0073300.1*
Acyan05G0123800.1	*Acyan03G0141700.1*	*Acyan04G0162300.1*	*Acyan01G0077900.1*

Gene IDs			
Acyan01G0262200.1	*Acyan09G0121100.1*	*Acyan04G0090800.1*	*Acyan06G0154300.1*
Acyan01G0293100.1	*Acyan07G0047800.1*	*Acyan09G0152000.1*	*Acyan04G0139400.1*
Acyan01G0295400.1	*Acyan06G0146500.1*	*Acyan03G0142800.1*	*Acyan03G0078700.1*
Acyan01G0335700.1	*Acyan06G0142800.1*	*Acyan04G0038700.1*	*Acyan09G0160600.1*
Acyan01G0336300.1	*Acyan06G0112900.1*	*Acyan01G0275500.1*	*Acyan12G0091500.1*
Acyan13G0171900.1	*Acyan06G0068200.1*	*Acyan11G0121800.1*	*Acyan09G0089400.1*
Acyan02G0288400.1	*Acyan06G0000400.1*	*Acyan01G0291900.1*	*Acyan09G0225900.1*
Acyan02G0251500.1	*Acyan08G0153500.1*	*Acyan11G0089500.1*	*Acyan12G0027300.1*
Acyan02G0208600.1	*Acyan13G0076000.1*	*Acyan04G0151600.1*	*Acyan10G0031300.1*
Acyan02G0183500.1	*Acyan02G0291700.1*	*Acyan08G0008800.1*	*Acyan04G0087400.1*
Acyan02G0178800.1	*Acyan01G0020600.1*	*Acyan02G0213500.1*	*Acyan05G0165300.1*
Acyan02G0150600.1	*Acyan13G0158800.1*	*Acyan10G0125500.1*	*Acyan12G0162000.1*
Acyan02G0053500.1	*Acyan02G0298400.1*	*Acyan07G0046900.1*	*Acyan11G0167300.1*
Acyan10G0147100.1	*Acyan09G0110900.1*	*Acyan05G0126300.1*	*Acyan05G0086500.1*
Acyan10G0091000.1	*Acyan04G0077200.1*	*Acyan01G0037600.1*	*Acyan08G0027100.1*
Acyan01G0005100.1	*Acyan07G0142700.1*	*Acyan01G0149500.1*	*Acyan04G0280200.1*
Acyan05G0131100.1	*Acyan04G0073600.1*	*Acyan07G0051500.1*	*Acyan01G0014400.1*
Acyan02G0324900.1	*Acyan13G0037400.1*	*Acyan01G0200400.1*	*Acyan10G0076200.1*
Acyan10G0159900.1	*Acyan05G0019300.1*	*Acyan05G0046300.1*	*Acyan05G0031000.1*
Acyan01G0099900.1	*Acyan08G0014600.1*	*Acyan01G0122800.1*	*Acyan05G0018200.1*
Acyan04G0070100.1	*Acyan05G0176700.1*	*Acyan01G0034600.1*	*Acyan04G0037100.1*
Acyan04G0164500.1	*Acyan04G0033200.1*	*Acyan02G0077800.1*	*Acyan04G0156200.1*
Acyan05G0145200.1	*Acyan08G0117900.1*	*Acyan05G0127800.1*	*Acyan09G0089300.1*
Acyan11G0077600.1	*Acyan01G0302500.1*	*Acyan05G0034800.1*	*Acyan07G0186300.1*
Acyan12G0061300.1	*Acyan01G0014500.1*	*Acyan05G0177900.1*	*Acyan06G0041500.1*
Acyan03G0188400.1	*Acyan10G0023700.1*	*Acyan04G0008200.1*	*Acyan07G0114900.1*
Acyan07G0136800.1	*Acyan11G0169000.1*	*Acyan05G0048900.1*	*Acyan06G0104300.1*
Acyan03G0045300.1	*Acyan02G0098300.1*	*AcyanUnG0035800.1*	*Acyan10G0101700.1*
Acyan09G0174300.1	*Acyan05G0028900.1*	*Acyan07G0106400.1*	*Acyan10G0072900.1*

Table S12. The 65 orthogroups that have PSGs in *A. catalpifolium* or *A. yangbiense*, and their corresponding annotated TFs family name.

Orthogroup ID	TFs family name
13Plants05148	AP2
13Plants08605	AP2
13Plants02468	bHLH
13Plants02740	bHLH
13Plants02869	bHLH
13Plants04977	bHLH
13Plants05978	bHLH
13Plants06847	bHLH
13Plants06941	bHLH
13Plants08444	bHLH
13Plants08570	bHLH
13Plants02347	bZIP
13Plants04028	bZIP
13Plants04856	bZIP
13Plants05427	bZIP
13Plants08183	bZIP
13Plants14631	bZIP
13Plants02722	C2H2
13Plants03430	C2H2
13Plants06881	C2H2
13Plants06946	C2H2
13Plants07034	C2H2
13Plants08735	C2H2
13Plants05227	C3H
13Plants02568	CAMTA
13Plants12666	Dof
13Plants05717	ERF
13Plants08143	ERF
13Plants06002	G2-like

Orthogroup ID	TFs family name
13Plants15485	GATA
13Plants04272	GRAS
13Plants06829	GRAS
13Plants03879	GRF
13Plants07349	LSD
13Plants03742	MIKC_MADS
13Plants04212	MIKC_MADS
13Plants06909	MIKC_MADS
13Plants01175	M-type_MADS
13Plants03435	MYB
13Plants05735	MYB
13Plants07463	MYB
13Plants03836	MYB_related
13Plants02482	NAC
13Plants05794	NAC
13Plants05833	NAC
13Plants05972	NAC
13Plants05984	NAC
13Plants13830	NAC
13Plants02801	NF-YA
13Plants08795	NF-YB
13Plants09404	SBP
13Plants02239	TALE
13Plants04270	TALE
13Plants05673	TALE
13Plants01912	TCP
13Plants03095	TCP
13Plants09603	TCP
13Plants11976	TCP
13Plants04303	Trihelix
13Plants04871	Trihelix
13Plants08588	WOX

Orthogroup ID	TFs family name
13Plants02326	WRKY
13Plants12594	WRKY
13Plants12636	WRKY
13Plants04258	ZF-HD

Table S13. Detailed information on the 109 genes encoding for key enzymes in lignin biosynthesis in *A. catalpifolium*. Significantly expanded genes are marked with superscript asterisks.

**Enzymes name (EC number)**	**Gene ID**
PAL (EC 4.3.1.24)	*EVM0015705.1*
	EVM0009331.1
	EVM0008576.1
	EVM0025958.1
	EVM0029592.1
	EVM0001964.1
	EVM0007836.1
	EVM0034557.1
	EVM0033269.1
	EVM0032337.1
	EVM0011567.1
	EVM0034110.1
C4H (EC 1.14.13.11)	*EVM0022411.1*
	EVM0033614.1
4CL (EC 6.2.1.12)	*EVM0020536.1*
	EVM0021765.1
	EVM0031874.1
	EVM0020225.1
CCR (EC 1.2.1.44)	*EVM0030228.1*
	EVM0032951.1
	EVM0016374.1
	EVM0018124.1
	EVM0015938.1
	EVM0002952.1
	EVM0027854.1
	EVM0017455.1
	EVM0014843.1
	EVM0005620.1

CAD (EC 1.1.1.195)	*EVM0014777.1*
	EVM0031573.1
	EVM0021768.1
	EVM0030914.1
	EVM0020255.1
	EVM0015357.4[*]
	EVM0011199.1
	EVM0002926.1[*]
	EVM0017562.1
	EVM0031977.1
	EVM0023767.1
	EVM0015729.1
	EVM0034736.1[*]
	EVM0004701.1
	EVM0015715.1
	EVM0016851.1
	EVM0014146.1
	EVM0005473.1
	EVM0032121.1
	EVM0028717.1
	EVM0019286.1
	EVM0004977.1
	EVM0016518.1
	EVM0016200.1
	EVM0030453.1
	EVM0029084.1[*]
	EVM0005700.1
	EVM0033367.1
	EVM0025232.1[*]
	EVM0010655.1
	EVM0009986.1
	EVM0033187.1[*]

CAD (EC 1.1.1.195) *continued*	*EVM0018275.1*
	EVM0020813.1[*]
	EVM0022102.1
	EVM0018630.1
	EVM0000981.1[*]
HCT (EC 2.3.1.133)	*EVM0004579.1*
	EVM0001363.1
	EVM0008965.1
	EVM0008149.1
	EVM0010807.1
	EVM0029023.1
	EVM0000744.1
	EVM0028997.1
	EVM0032850.1
	EVM0005833.1
	EVM0009078.1
	EVM0032819.1
	EVM0021098.1
	EVM0013757.1
	EVM0005635.1
	EVM0031142.1
	EVM0025316.1
	EVM0008337.1
	EVM0021351.1
	EVM0001231.1
	EVM0031176.1
	EVM0021330.1
C3H (EC: 1.14.13.36)	*EVM0008838.1*
	EVM0021276.1
	EVM0001582.1

CCoAOMT (EC 2.1.1.104)	*EVM0010444.1*
	EVM0011640.1
	EVM0012450.2
	EVM0017409.1
	EVM0029897.1
F5H (EC:1.14.-.-)	*EVM0026174.1*
	EVM0028050.1
	EVM0029634.1
	EVM0028881.1
	EVM0002270.1
	EVM0010679.1
	EVM0002821.1
	EVM0015236.1
COMT (EC 2.1.1.68)	*EVM0011911.1*
	EVM0015811.1
	EVM0022914.1
	EVM0034657.1
	EVM0018788.1
	EVM0013202.1

Table S14. Detailed information on the 96 genes encoding for key enzymes in lignin biosynthesis in *A. yangbiense*.

Enzymes name (EC number)	Gene ID
PAL (EC 4.3.1.24)	*Acyan12G0110700.1*
	Acyan12G0110200.1
	Acyan12G0110900.1
	Acyan07G0110500.1
	Acyan07G0076400.1
	Acyan12G0110500.1
	Acyan12G0110600.1
	Acyan07G0110400.1
	Acyan07G0076300.1
	Acyan12G0116900.1
	Acyan01G0268400.1
	Acyan05G0098100.1
	Acyan12G0110300.1
C4H (EC 1.14.13.11)	*Acyan13G0018300.1*
	Acyan05G0045800.1
4CL (EC 6.2.1.12)	*Acyan05G0032000.1*
	Acyan02G0143400.1
	Acyan12G0026200.1
	Acyan02G0182500.1
CCR (EC 1.2.1.44)	*Acyan05G0018400.1*
	Acyan10G0003800.1
	Acyan12G0027600.1
	Acyan10G0004300.1
	Acyan13G0001100.1
	Acyan10G0004200.1
	Acyan10G0004500.1
	Acyan09G0127900.1
	Acyan13G0001200.1
	Acyan10G0004700.1

CCR (EC 1.2.1.44) *continued*	*Acyan13G0001800.1*
	Acyan01G0331800.1
CAD (EC 1.1.1.195)	*Acyan06G0089100.1*
	Acyan03G0036700.1
	Acyan08G0011500.1
	Acyan06G0089500.1
	Acyan06G0088900.1
	Acyan04G0194400.1
	Acyan08G0012800.1
	Acyan06G0089300.1
	Acyan08G0012500.1
	Acyan06G0087800.1
	Acyan03G0036600.1
	Acyan02G0053300.1
	Acyan08G0012000.1
	Acyan06G0089400.1
	Acyan06G0087700.1
	Acyan08G0012200.1
	Acyan03G0036800.1
	Acyan06G0015200.1
	Acyan04G0194500.1
	Acyan01G0209100.1
	Acyan03G0036900.1
	Acyan04G0194600.1
	Acyan08G0011700.1
	Acyan06G0089200.1
	Acyan04G0194700.1
HCT (EC 2.3.1.133)	*Acyan01G0240800.1*
	Acyan06G0019100.1
	Acyan06G0019500.1
	Acyan11G0112000.1
	Acyan10G0066300.1

HCT (EC 2.3.1.133) *continued*	*Acyan10G0066200.1*
	Acyan12G0152200.1
	Acyan03G0048800.1
	Acyan03G0088400.1
	Acyan11G0112100.1
	Acyan09G0159700.1
	Acyan06G0019000.1
	Acyan06G0018300.1
	Acyan06G0018700.1
	Acyan07G0140500.1
	Acyan06G0019300.1
	Acyan02G0141900.1
	Acyan06G0018500.1
	Acyan06G0019400.1
	Acyan06G0018800.1
	Acyan06G0019200.1
C3H (EC: 1.14.13.36)	*Acyan11G0032300.1*
	Acyan11G0032200.1
CCoAOMT (EC 2.1.1.104)	*Acyan05G0088300.1*
	Acyan05G0088000.1
	Acyan04G0200900.1
	Acyan05G0088200.1
F5H (EC:1.14.-.-)	*Acyan06G0056600.1*
	Acyan04G0239500.1
	Acyan04G0239400.1
	Acyan02G0001800.1
	Acyan04G0239600.1
	Acyan06G0140900.1
COMT (EC 2.1.1.68)	*Acyan12G0118400.1*
	Acyan11G0066700.1
	Acyan01G0312400.1
	Acyan12G0003300.1

COMT (EC 2.1.1.68) *continued*	*Acyan03G0138600.1*
	Acyan12G0118200.1
	Acyan12G0003200.1

Table S15. Detailed information on the 99 genes encoding for key enzymes in lignin biosynthesis in *P. trichocarpa*. Significantly expanded genes are marked with superscript asterisks.

Enzymes name (EC number)	Gene ID
PAL (EC 4.3.1.24)	*Potri.016G091100.1*
	Potri.010G224200.1
	Potri.010G224100.1
	Potri.006G126800.1
	Potri.008G038200.1
C4H (EC 1.14.13.11)	*Potri.018G146100.1*
	Potri.019G130700.1
	Potri.013G157900.1
	Potri.006G078100.1
4CL (EC 6.2.1.12)	*Potri.017G138400.1**
	Potri.006G169700.1
	Potri.001G036900.1
	Potri.018G094200.1
	Potri.006G169600.1
	Potri.T116500.1*
	Potri.003G188500.1
	Potri.019G049500.1
CCR (EC 1.2.1.44)	*Potri.001G046100.1*
	Potri.001G045500.1
	Potri.001G045900.1
	Potri.018G100500.1
	Potri.006G178700.1
	Potri.004G230900.1
	Potri.004G105000.1
	Potri.001G140700.1
	Potri.001G045300.1
	Potri.T134100.1
	Potri.001G046400.1

CCR (EC 1.2.1.44) *continued*	*Potri.001G349600.1*
	Potri.013G079500.1
	Potri.001G045800.1
	Potri.003G181400.1
	Potri.001G045100.1
	Potri.001G045600.1
	Potri.017G110500.1
	Potri.001G045700.1
	Potri.002G004100.1
	Potri.003G093700.1
	Potri.001G045400.1
	Potri.001G045000.1
	Potri.009G076300.1
CAD (EC 1.1.1.195)	*Potri.001G256400.1*
	Potri.T149300.1
	Potri.009G063300.1
	Potri.016G078300.1
	Potri.009G063400.1
	Potri.009G057500.1
	Potri.009G052000.1
	Potri.001G268600.1
	Potri.009G057700.1
	Potri.T161300.1
	Potri.001G300000.1
	Potri.009G057600.1
	Potri.006G199100.1
	Potri.009G095800.1
	Potri.016G023300.1
	Potri.016G065300.1
	Potri.009G062900.1
	Potri.009G062800.1
	Potri.009G057800.1

CAD (EC 1.1.1.195) *continued*	*Potri.007G049200.1*
	Potri.005G243700.1
	Potri.009G063100.1
HCT (EC 2.3.1.133)	*Potri.014G025500.1*
	Potri.016G112400.1
	Potri.010G192400.1
	Potri.T178300.1
	Potri.012G144500.1
	Potri.008G065000.1
	Potri.001G042900.1
	Potri.003G057100.1
	Potri.014G025600.1
	Potri.006G097500.1
	Potri.003G057000.1
	Potri.005G028400.1
	Potri.003G183900.1
C3H (EC: 1.14.13.36)	*Potri.002G060900.1*
	Potri.005G200400.1
	Potri.016G031000.1
CCoAOMT (EC 2.1.1.104)	*Potri.008G136600.1*
	Potri.018G070300.1
	Potri.009G099800.1
	Potri.010G104400.1
	Potri.008G136700.1
	Potri.002G183600.1
	Potri.001G304800.1
F5H (EC:1.14.-.-)	*Potri.009G123600.1*
	Potri.004G161600.1
	Potri.007G016400.1
	Potri.005G117500.1
COMT (EC 2.1.1.68)	*Potri.001G451100.1*
	Potri.015G003100.1

COMT (EC 2.1.1.68) *continued*	*Potri.012G006400.1*
	Potri.002G180500.1
	Potri.019G084300.1
	Potri.014G106500.1
	Potri.011G150500.1
	Potri.002G180600.1
	Potri.016G101600.1

References

Yang, J., Wariss, H.M., Tao, L., Zhang, R., Yun, Q., Hollingsworth, P., Dao, Z., Luo, G., Guo, H., Ma, Y., and Sun, W. (2019). De novo genome assembly of the endangered *Acer yangbiense*, a plant species with extremely small populations endemic to Yunnan Province, China. ***Gigascience*** 8: giz085.
